# COVID-19 virtual patient cohort suggests immune mechanisms driving disease outcomes

**DOI:** 10.1371/journal.ppat.1009753

**Published:** 2021-07-14

**Authors:** Adrianne L. Jenner, Rosemary A. Aogo, Sofia Alfonso, Vivienne Crowe, Xiaoyan Deng, Amanda P. Smith, Penelope A. Morel, Courtney L. Davis, Amber M. Smith, Morgan Craig

**Affiliations:** 1 Sainte-Justine University Hospital Research Centre, Montréal, Québec, Canada; 2 Department of Mathematics and Statistics, Université de Montréal, Montréal, Québec, Canada; 3 Department of Pediatrics, University of Tennessee Health Science Center, Memphis, Tennessee, United States of America; 4 Department of Physiology, McGill University, Montréal, Québec, Canada; 5 Department of Mathematics and Statistics, Concordia University, Montréal, Québec, Canada; 6 Department of Immunology, University of Pittsburgh, Pittsburgh, Pennsylvania, United States of America; 7 Natural Science Division, Pepperdine University, Malibu, California, United States of America; Columbia University Medical Center, UNITED STATES

## Abstract

To understand the diversity of immune responses to SARS-CoV-2 and distinguish features that predispose individuals to severe COVID-19, we developed a mechanistic, within-host mathematical model and virtual patient cohort. Our results suggest that virtual patients with low production rates of infected cell derived IFN subsequently experienced highly inflammatory disease phenotypes, compared to those with early and robust IFN responses. In these *in silico* patients, the maximum concentration of IL-6 was also a major predictor of CD8^+^ T cell depletion. Our analyses predicted that individuals with severe COVID-19 also have accelerated monocyte-to-macrophage differentiation mediated by increased IL-6 and reduced type I IFN signalling. Together, these findings suggest biomarkers driving the development of severe COVID-19 and support early interventions aimed at reducing inflammation.

## Author summary

Understanding of the diversity of immune responses to SARS-CoV-2 infections is critical for improving diagnostic and treatment approaches. Identifying which immune mechanisms lead to divergent outcomes can be clinically difficult, and experimental models and longitudinal data are only beginning to emerge. In response, we developed a mechanistic, mathematical and computational model of the immunopathology of COVID-19 calibrated to and independently validated against a broad set of experimental and clinical immunological data. To study the drivers of severe COVID-19, we used our model to expand a cohort of virtual patients, each with realistic disease dynamics. Our results suggest key processes that regulate the immune response to SARS-CoV-2 infection in virtual patients and suggest viable therapeutic targets, underlining the importance of a rational, multifaceted approach to studying novel pathogens using intra-host models.

## Introduction

Clinical manifestations of SARS-CoV-2 infection are heterogeneous, with a significant proportion of people experiencing asymptomatic or mild infections that do not require hospitalization. In severe cases, patients develop coronavirus disease (COVID-19) that may progress to acute respiratory distress syndrome (ARDS), which is frequently accompanied by myriad inflammatory indicators [1]. Mounting evidence points to a hyper-reactive and dysregulated inflammatory response characterized by overexpression of pro-inflammatory cytokines (cytokine storm) and severe immunopathology as specific presentations in severe COVID-19 [2–6]. An over-exuberant innate immune response with larger numbers of infiltrating neutrophils [7,8] arrests the adaptive immune response through the excessive release of reactive oxygen species that leads to extensive tissue damage and depletion of epithelial cells [9]. In addition, T cell lymphopenia, in particular, is one of the most prominent markers of COVID-19 and has been observed in over 80% of patients with severe disease [6,10–12]. However, the immune mechanisms that lead to disparate outcomes during SARS-CoV-2 infection remain to be delineated.

Cytokines are critically important for controlling virus infections [13,14] and are central to the pathophysiology of COVID-19, sometimes playing a detrimental role in the context of a cytokine storm [10]. For example, interleukin-6 (IL-6) can stimulate CD8^+^ T cell expansion under inflammatory conditions [15]; however, in hospitalized SARS-CoV-2 patients with lymphopenia, IL-6 has been shown to be elevated [16] without an increase in CD8^+^ T cell counts [17]. Type I interferons (such as IFNs- α, β [18]) also play a major role in limiting viral replication by inducing a refractory state in susceptible and infected cells [19–21]. Due to this, it has been suggested that a delay in mounting an effective IFN response may be responsible for COVID-19 severity [10] as it is for other highly pathogenic coronavirus (i.e. SARS-CoV and MERS) infections [13]. Interferon delays/dysregulation contributing to COVID-19 severity are a known feature of coronavirus infections in humans [22] and type-I interferons are known to downregulate viral replication in infected and neighbouring cells [22]. The emerging experimental and clinical pictures of the impact of IFN-I dynamics on SARS-CoV-2 infections is consistent with both SARS-CoV and MERS [23–26]. Further, retrospective and clinical trials have shown that earlier administrations of IFN was associated with or significantly improved survival rates [27,28]. Overall, patients with severe COVID-19 present with lymphopenia [14,29], and are likely to have increased inflammatory cytokines such as IL-6, granulocyte-macrophage colony-stimulating factor (GM-CSF), and granulocyte colony-stimulating factor (G-CSF) [7,17,30].

Because the identification of immune mechanisms responsible for divergent disease outcomes can be difficult clinically, and experimental models and longitudinal data are only beginning to emerge, theoretical explorations are ideal [31]. Quantitative approaches combining mechanistic disease modelling and computational strategies are being increasingly leveraged to investigate inter- and intra-patient variability by, for example, developing virtual clinical trials [32–34]. Such *in silico* trials enable the theoretical exploration of how multiple key underlying mechanisms simultaneously impact disease severity [34]. More recently, viral dynamics models [35,36] have been applied to understand SARS-CoV-2 within-host dynamics and their implications for therapy [37–42]. However, there are few comprehensive models that integrate detailed immune mechanisms and allow interrogation of the dynamics controlling divergent outcomes, and none have attempted to quantify the high degree of variability in patient responses to SARS-CoV-2 through modelling.

In this study, we developed a mechanistic mathematical model to describe the within-host immune response to SARS-CoV-2. We explicitly modelled the interactions between epithelial cells, innate and adaptive immune cells, and cytokines. The model was fit to various *in vitro*, *in vivo*, and clinical data, analyzed to predict how early infection kinetics facilitate downstream disease dynamics, and used to create a virtual patient cohort with realistic disease courses. Our results suggest that mild and severe disease are distinguished by the rates of monocyte differentiation into macrophages and of IFN production by infected cells. In our virtual cohort, we found that severe COVID-19 responses were tightly correlated with a delay in the peak IFN concentration and that a large increase in IL-6 was the dominant predictor of CD8^+^ T cell depletion. These results provide insight into differential presentations of COVID-19 by suggesting key regulators of severe disease manifestation particularly related to monocyte differentiation and IL-6 concentrations. Importantly, these key mechanisms suggest promising avenues of experimental research into the mechanisms of immunopathology in COVID-19.

## Results

### Modelling the immune response to SARS-CoV-2 and the impact of delayed IFN on infection dynamics

**Fig 1:**
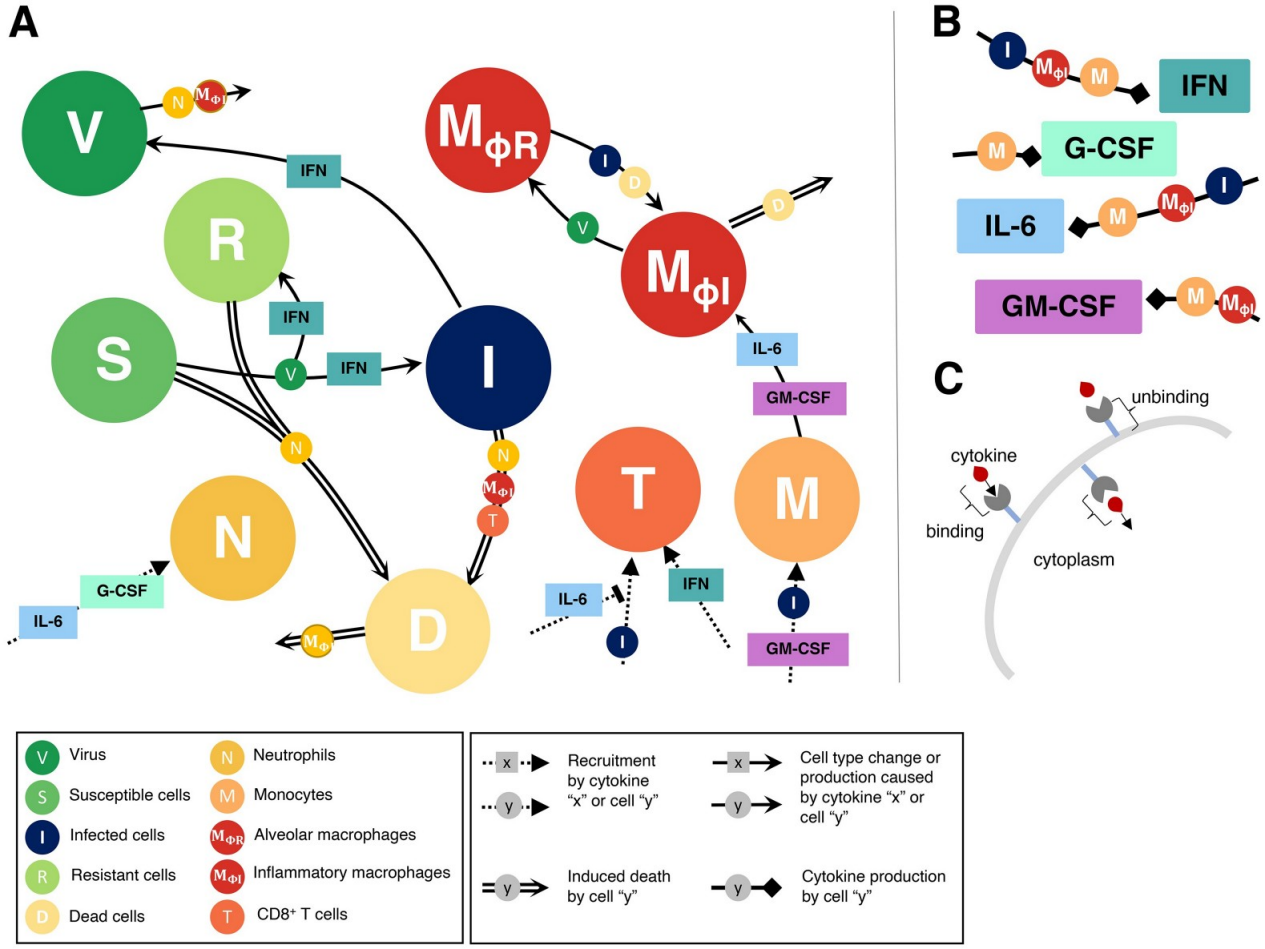
Immune response to SARS-CoV-2 infection model schematic. The model in **Eqs. S1-S22** reduced to **A)** cell dynamics **B)** cytokine production dynamics and **C)** cytokine binding kinetics. Unique lines represent induced cell death (double line), recruitment (dashed line), cell type change or production (solid line), and cytokine production (square arrow). Cell and/or cytokines along joining lines denote a causal interaction. **A)** Virus (*V*) infects susceptible lung epithelial cells and creates either infected (*I*) or resistant (*R*) cells depending on the concentration of type I IFN. Infected cells then either die and produce new virus or are removed via inflammatory macrophages (*M*_*ΦI*_) or CD8^+^ T cells (*T*) that induce apoptosis to create dead cells (*D*). Neutrophils (N) cause bystander damage (death) in all epithelial cells and are recruited by individually G-CSF and IL-6 concentrations. CD8^+^ T cells are recruited by infected cells and their population expands from IFN signalling. T cell recruitment is inhibited by IL-6 concentrations. Monocytes (*M*) are recruited by infected cells and GM-CSF and differentiate into inflammatory macrophages based on the individual concentrations of GM-CSF and IL-6. Tissue-resident macrophages (*M*_*ΦR*_) also become inflammatory macrophages through interaction with dead and infected cells. Dead cells are cleared up by inflammatory macrophages and also cause their death. **B)** Type I IFN is produced by infected cells, inflammatory macrophages and monocytes. G-CSF is produced solely by monocytes and GM-CSF is produced by monocytes and macrophages. IL-6 is produced by monocytes, inflammatory macrophages and infected cells. **C)** Cytokine receptor binding, internalization and unbinding kinetics considered for each cell-cytokine interaction.

To study the dynamics of SARS-CoV-2 infection and the development of COVID-19, we constructed a computational biology model of host-pathogen interactions (**Eqs. S1-S22**, with variables and parameters summarized in S1 Table and schematic in Fig 1). The model includes susceptible lung epithelial cells (*S*) that encounter virus (*V*) and become infected (*I*) before turning into damaged or dead cells (*D*) due to viral infection or immune involvement. The immune response is orchestrated by cytokines that act to stimulate the immune cell subsets present in the tissues and recruit cells from the bone marrow and circulation (Fig 1A). We have chosen to model type I IFN, IL-6, G-CSF, and GM-CSF because of their roles in viral resistance, inflammation, recruitment and differentiation, respectively. Upon infection, lung epithelial cells secrete type I IFNs (*F*) that cause adjacent cells to become resistant to infection (*R*) and decrease the production of newly infected cells [43]. Through stimulation by infected and dead cells, alveolar (lung tissue-resident) macrophages (*M*_*ΦR*_) become inflammatory macrophages (*M*_Φ*I*_), which also arise through monocyte (*M*) differentiation following stimulation by GM-CSF (*G*) or IL-6 (*L*) [44]. Neutrophils (*N*) are recruited to the infection site by G-CSF and release reactive oxygen species (ROS) causing bystander damage to infected and susceptible cells [45,46]. CD8^+^ T cells (*T*) are subsequently recruited to the infection site following a delay to account for antigen presentation, with expansion modulated by type I IFN and IL-6 concentrations. See Materials and methods for a complete description.

**Fig 2:**
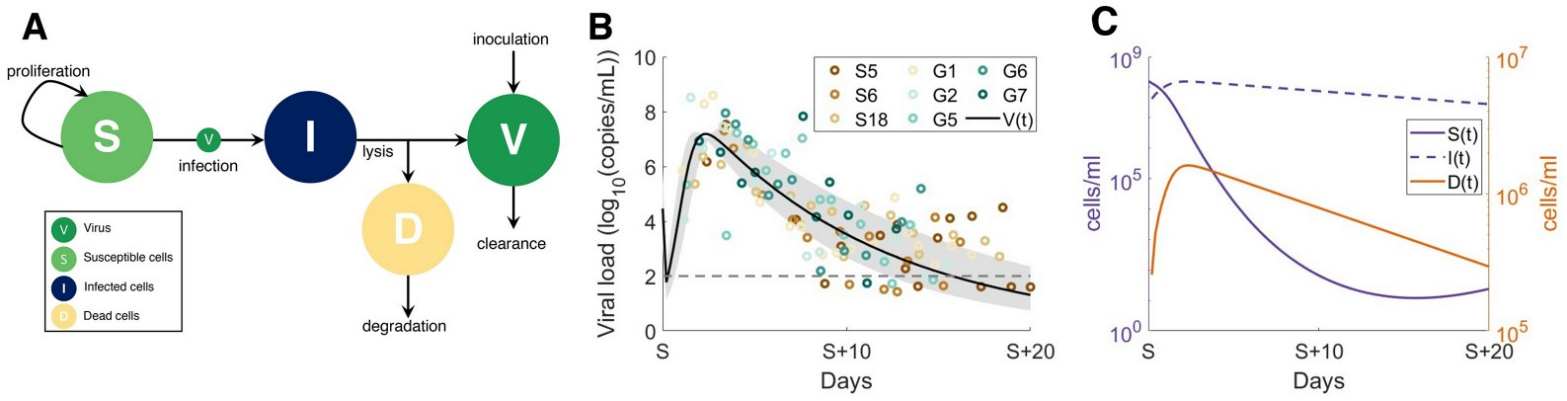
Viral dynamics model fit to human viral data from hospitalized patients in Singapore and Germany. A reduced version of the full model (all cytokine and immune cells set to 0, Eqs 6–9) was fit to data from hospitalized patients, after initial estimation from viral loads in macaques [47] (S6 Fig) to estimate preliminary viral kinetic parameters. **A)** Virus (*V*) infects susceptible cells (*S*) making infected epithelial cells (*I*) which then die to produce dead cells (*D*) and new virus. **B)** Viral load data (log_10_(copies/mL) from eight human patients (three from Singapore S5, S6 and S18, and five from Germany, G1, G2, G5, G6, G7) were digitized from previous results [50], and parameters from the viral dynamics submodel were estimated using a non-linear squares optimization routine. *β*, *d*_*I*_, *V*_0_ and *d*_*V*_ were estimated from the reduced viral dynamics model in A) (see Methods and S1 Table). Individual patient measurements are depicted by coloured circles. Solid black line: average model prediction; grey shaded region: predicted standard deviation from average. S (time axis) indicates the day of symptom onset.

Because the model has several parameters that are undetermined biologically and insufficient data exists to confidently estimate their values, we used a stepwise approach to parameter estimation (see Materials and methods and S1–S5 Figs). We chose this approach to ensure that individual aspects of a given biological interaction could be better understood in isolation and to help reduce the degrees of freedom at each stage of parameter fitting. We first confirmed that we could recapitulate early infection viral kinetics with a reduced version of the full model (‘viral model’). For this, we excluded immunological variables (i.e. only including Eqs 6–9) and estimated parameters relating to viral kinetics by fitting to viral load data from macaques [47] and then viral load in humans hospitalized in Singapore [48] and Germany [49] (see Materials and methods). The resulting model dynamics were in good agreement to these early infection data (see S6 Fig for the macaque and Fig 2 for the human SARS-CoV-2 viral dynamics) and demonstrate a rebound in epithelial lung tissue as the viral load and infected cells decrease.

**Fig 3:**
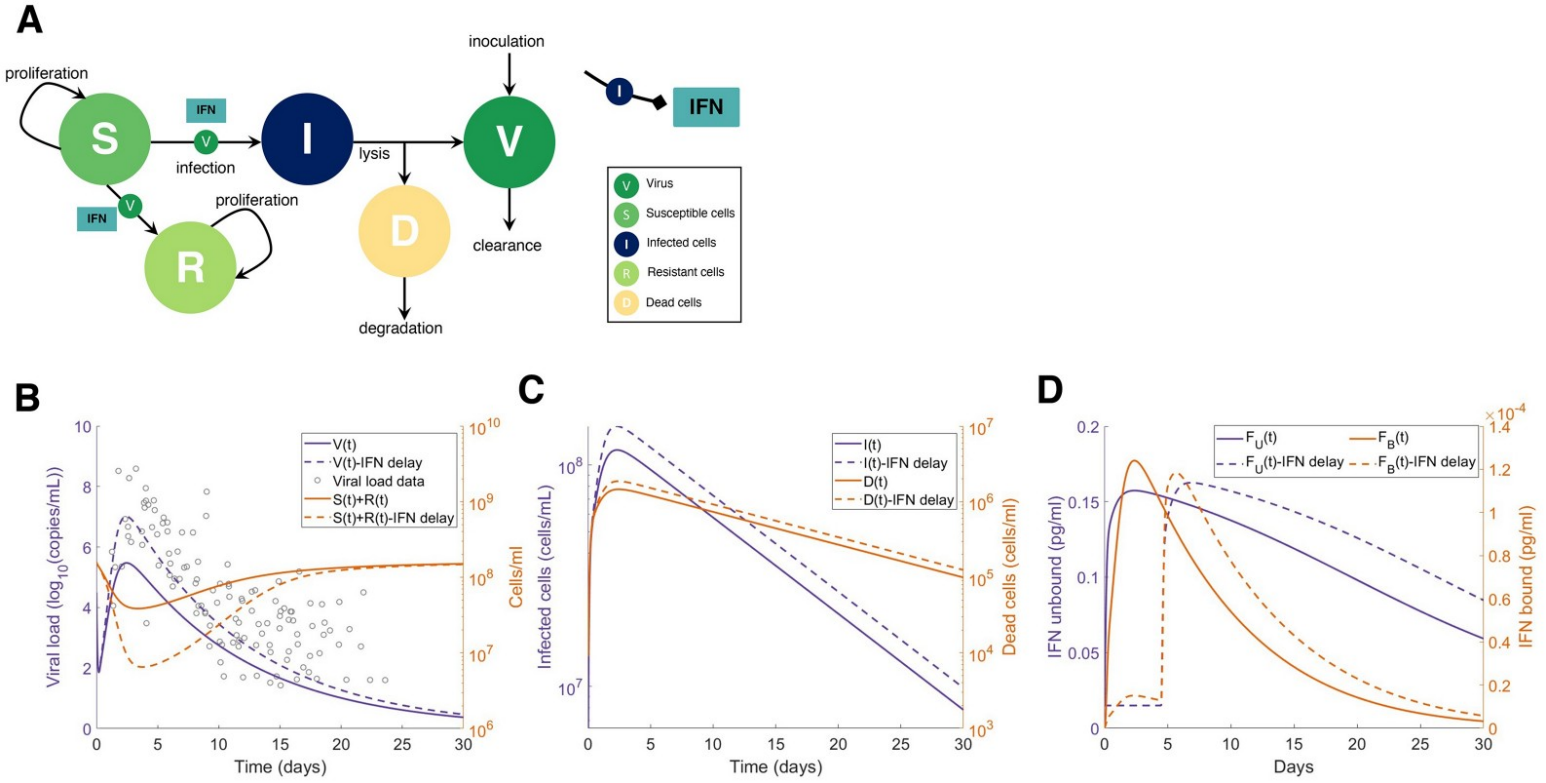
Delayed type I IFN response impacts heavily on tissue survival in reduced model. **A)** Submodel (Eqs 10–16) with all non-IFN cytokines and immune cell interactions set to zero and only considering interactions between virus (*V*), type I IFN, and susceptible (*S*), infected (*I*), resistant (*R*), and dead (*D*) epithelial cells. **B)** Predictions from the simplified model without delayed IFN production (solid lines) versus with a constant delay (*τ*_*F*_ = 5 days) (dotted lines). Grey circles (left panel): viral loads from SARS-CoV-2 infection in humans in Singapore [48] and Germany [49], digitized from Goyal et al. [50] overlayed with predicted viral dynamics. **C-D)** Predicted dynamics of infected and dead cells, and unbound and bound IFN concentrations from the simplified model without delayed IFN production (solid lines) versus with a constant delay (*τ*_*F*_ = 5 days) (dotted lines).

We then isolated the IFN dynamics to assess clinical and experimental findings suggesting that delaying IFN results in more severe presentations in highly pathogenic coronavirus infections including SARS-CoV-2 [10,13,14]. Using the parameters obtained from the ‘viral model’ (Eqs 6–9; S1 Table), we then simulated the impact of IFN with the ‘IFN model’ (Eqs 10–16 and Fig 3). We examined the predicted dynamics in response to delayed IFN by simulating with and without a fixed delay for IFN production from infected cells. Our results suggest that delaying type I IFN production by 5 days yields a roughly 10-fold increase in tissue damage with tissue remaining on day 3 decreasing from 3.9 × 10^7^ cells/ml to 6.5 × 10^6^ cells/ml (Fig 3B), caused by the increase in infected cells and subsequent lack of resistant cells. IFN dynamics were within the observed ranges of systemic IFN- α concentrations from clinical cohorts of hospitalized COVID-19 patients [51] (S7A Fig). As patient IFN-α measurements are only taken after hospitalization and in plasma, there are currently no known sources of early SARS-CoV-2 infection IFN dynamics in humans.

### Immunologic determinants of mild and severe disease

**Fig 4:**
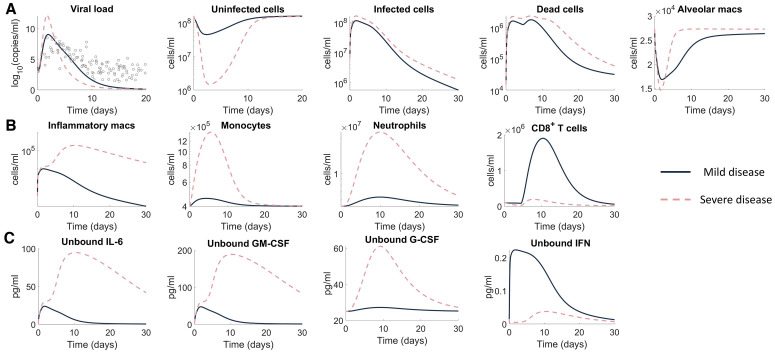
Predicting mild and severe COVID-19 dynamics. Mild disease (solid lines) dynamics obtained by using baseline parameter estimates (S1 Table) while severe disease dynamics (dashed lines) were obtained by decreasing the production rate of type I IFN (*P*_*F*,*I*_) and increasing the production of monocytes (*p*_*M*,*I*_) and their differentiation to macrophages ($\eta_{F,M_{\Phi I}}$). **A)** Viral load and lung cells concentrations (susceptible, resistant, infected, and dead cells). Solid black line with error bars indicates human data [50] (see Fig 2). **B)** Immune cell concentrations (inflammatory macrophages, monocytes, neutrophils, and CD8^+^ T cells). **C)** Unbound cytokine concentrations (free IL-6, GM-CSF, G-CSF, and type I IFN). Time evolution of all model variables is shown in S8 Fig (including bound cytokine and alveolar macrophages).

Next, to investigate the mechanisms that differentiate mild versus severe disease, we simulated the full model (**Eqs. S1-S22**) using two different parameter sets. Mild disease dynamics were recreated using estimated parameter values (S1 Table) such that the virus decay rate (*d*_*V*_) and the infected cell death rate (*d*_*I*_) were recalculated to ensure that the maximum death rate of the virus and infected cells did not exceed the value obtained from the reduced viral dynamics model fit (Fig 2). Simulating mild disease, we predicted that all cell populations and cytokines rapidly return to homeostasis, with the immune response effectively clearing virus within 10 days (Fig 4 and S8 Fig).

Because severe SARS-Cov-2 infection results in lower levels of IFN [51] and increased monocytes [52], we recapitulated severe disease by modulating model parameters relating to these processes, i.e., the rates of IFN production from infected cells, *p*_*F*,*I*_, and macrophages, $\eta_{F,M_{\Phi I}}$, were decreased, and the rate of monocyte recruitment from the bone marrow by infected cells, *p*_*M*,*I*_, was increased. With these changes, the model predicted a dramatic shift in disease response characterized by a cytokine storm (elevated IL-6, GM-CSF and G-CSF), high ratios of innate to adaptive immune cells, and a marked reduction in healthy viable lung tissue (Fig 4A), whereas changes in viral load remained relatively consistent with mild disease.

In addition, there was a notable increase in the number of inflammatory macrophages (Fig 4B), IL-6, GM-CSF and, importantly, a delayed and reduced IFN peak (Fig 4C). In comparison to mild disease, inflammatory macrophages and neutrophils remained elevated for at least 30 days after initial infection (Fig 4B). Comparing mild and severe disease highlighted differences in the area under the curve (AUC) of macrophages (6 × 10^4^ cells/ml versus 3 × 10^11^ cells/ml) and neutrophils (2 × 10^8^ cells/ml versus 3 × 10^13^ cells/ml) over 30 days. Interestingly, inflammation remained high in the severe disease scenario despite the virus being cleared slightly faster (~1 day) than in the case of mild disease (Fig 4A). Further, the peak of inflammatory macrophages increased from ~10^4^ cells/ml to ~10^6^ cells/ml in severe scenarios compared to mild scenarios. The model also accurately predicted that CD8^+^ T cell dynamics were lower in severe cases, which is indicative of lymphopenia and similar to clinical observations from patients with severe COVID-19 [14,23]. Despite varying only three parameters (*p*_*F*,*I*_, *p*_*M*,*I*_, and *η*_*F*,*M*Φ_) to generate disparate dynamics, the immune cell and cytokine dynamics were qualitatively in line with clinical observations for IFN-α [51], IL-6 [51,53], and G-CSF [30] (S7B–S7F Fig).

### Macrophages, CD8^+^ T cells, IFN and IL-6 regulate response to SARS-CoV-2 infection

**Fig 5:**
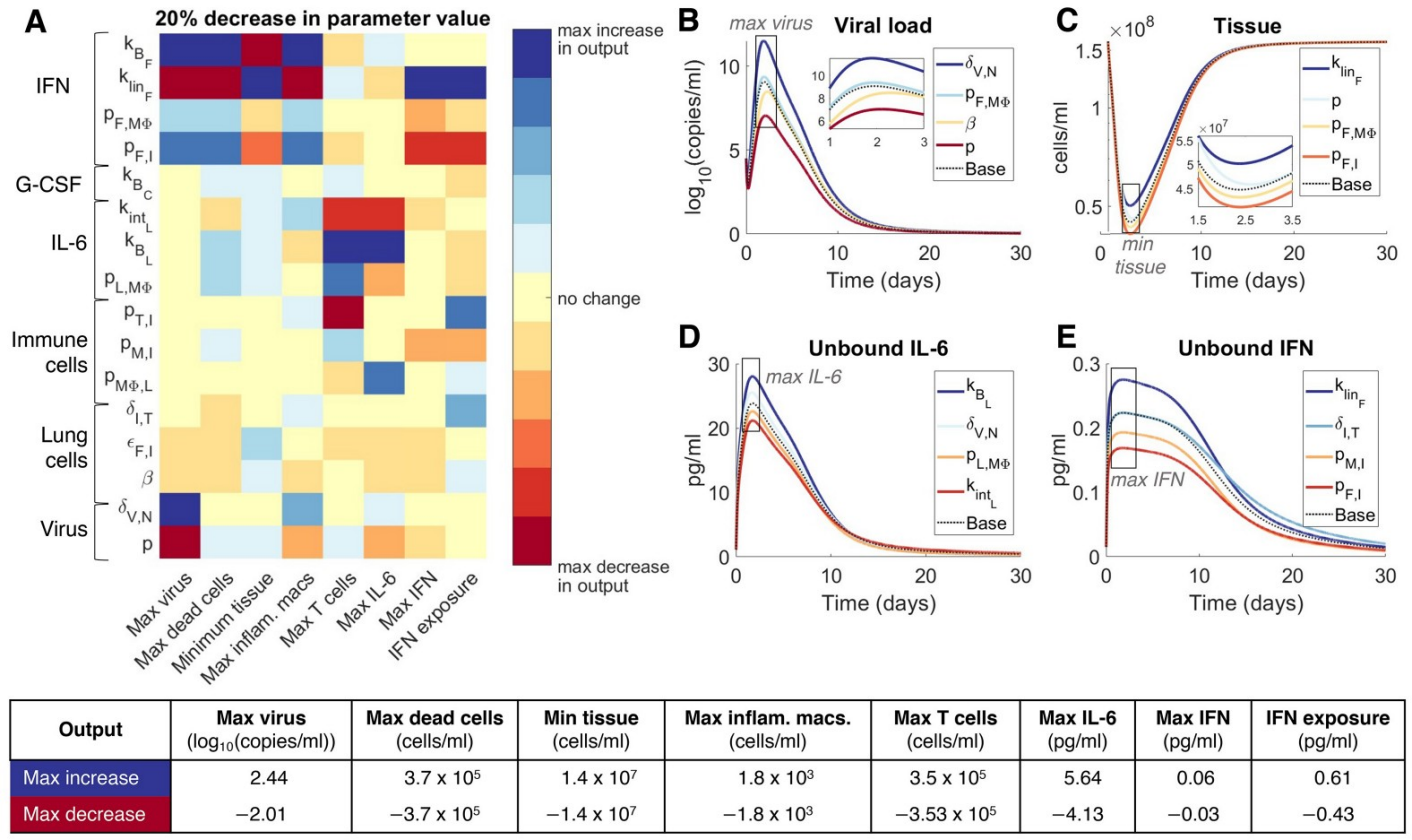
Parameters driving COVID-19 severity. A local sensitivity analysis was performed by varying each parameter ±20% from its originally estimated value (mild disease parameters in Fig 4) and simulating the model. Predictions were then compared to baseline considering: Maximum viral load (max(*V*)), maximum concentration of dead cells (max(*D*)), minimum uninfected live cells (min(*S+R*)), maximum concentration of inflammatory macrophages (max(*M*_*ΦI*_)), maximum number of CD8^+^ T cells (max(*T*)), maximum concentration of IL-6 (max(*L*_*U*_)), maximum concentration of type I IFN (max(*F*_*U*_)) and the total exposure to type I IFN (*F*_*U*_ exposure). **A)** Heat map shows the magnitude of the change of each metric from a 20% decrease in the parameter value compared to baseline (i.e. model simulation with no change in the parameter values), where blue signifies the maximum value observed in the output metric and red signifies the minimum value observed (i.e. maximum decrease in that metric). The most sensitive parameters are shown here, for the complete parameter sensitivity results, see S9 Fig. The explicit value of the maximum increase and maximum decrease of each metric is given in the table below. **B)**-**E)** time-series dynamics of viral load, tissue (uninfected cells), and unbound IL-6 and IFN given 20% decreases in the noted parameters. Colours of the curves correspond to the colouring of the heatmap in **A**. Maximal(minimal) concentrations, as in A, are noted in grey boxes. **E)** is coloured according to IFN exposure. Base: original mild parameters (Fig 4).

To further understand how the host immune system regulates the response to SARS-CoV-2 infection, we conducted a local sensitivity analysis by varying each parameter individually by ±20% and comparing a set of metrics (see Materials and methods) chosen to provide a comprehensive understanding of each parameter’s impact on the host-pathogen dynamics (S9 Fig). This analysis identified 17 sensitive parameters (Fig 5A) relating to virus productivity (*p*, *δ*_*V*,*N*_, *β*, *ϵ*_*F*,*I*_), CD8^+^ T cell induced epithelial cell apoptosis (*δ*_*I*,*T*_), macrophages, monocyte and CD8^+^ T cell production ($p_{M\Phi_{I},L}$, *p*_*M*,*I*_, *p*_*T*,*I*_), IL-6 ($p_{L,M\Phi },k_{B_{L}},k_{int_{L}}$), G-CSF ($k_{B_{C}}$), and IFN (*p*_*F*,*I*_, *p*_*F*,*M*Φ_, $k_{lin_{F}},k_{B_{F}},k_{int_{F}}$). Viral load, uninfected cells (tissue), unbound IL-6 and unbound IFN dynamics after 20% decreases in parameters values were found to be similar to those in the original mild disease simulation (Fig 5B–5E).

Decreasing the rate of IL-6-induced monocyte differentiation into inflammatory macrophages (*p*_*MΦ*,*L*_) increased the peak concentration of both IL-6 and IFN (Fig 5D and 5E). Notably, changes to parameters that increased the bound IFN concentration, i.e. increasing the binding and production rates ($k_{B_{F}}$ and *p*_*F*,*I*_) and decreasing the internalization and clearance rates ($k_{lin_{F}}$ and $k_{int_{F}}$) induced significant changes in most metrics (Fig 5A), in particular decreases in $k_{lin_{F}}$ caused an increase in the minimum tissue and IFN (Fig 5C and 5E). The duration of extensive tissue damage (>80% damaged) increased with IFN potency (*ϵ*_*F*,*I*_) (S9 Fig). Less significant changes in the maximum dead cells and minimum tissue can also be seen in the viral infectivity (*β*) and IL-6 binding kinetic ($k_{int_{L}}$ and $k_{B_{L}}$) parameters. See S9 Fig for complete sensitivity analysis results.

**Fig 6:**
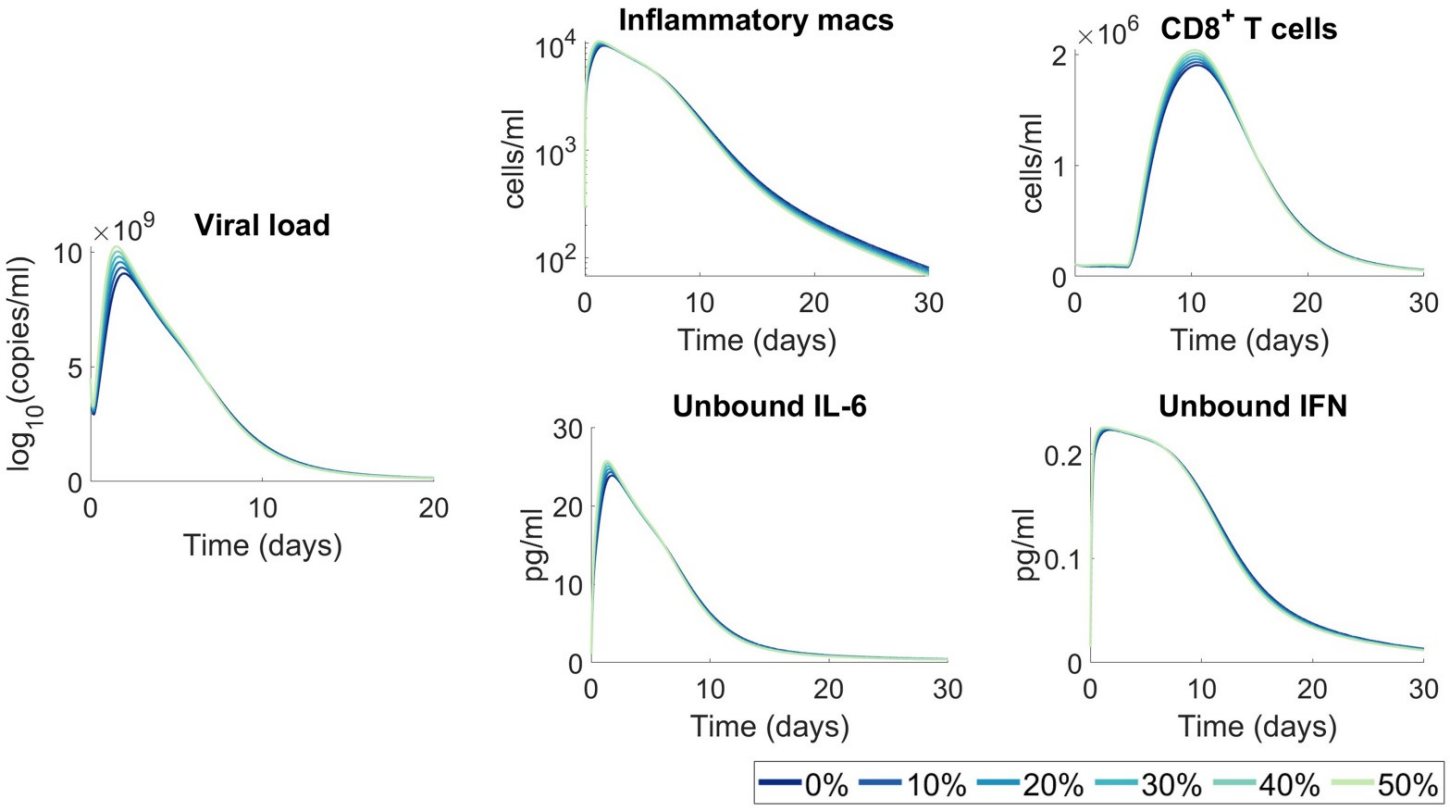
Moderate increases to viral infectivity are not predicted to significantly impact immunological outcomes. A range of viral infectivity rates (*β*) from 0% (base) to 50% increase were simulated. All other parameters were fixed to their value in S1 Table. The resulting model dynamics for viral load, inflammatory macrophages, CD8^+^ T cells, unbound IL-6 and unbound IFN were compared, and no significant changes in kinetics were predicted.

Changes to viral infectivity can be considered linked to two separate, but indistinguishable effects in our model, namely different densities of cellular ACE2 receptors between individuals and changes to viral infectivity through mutations. This would translate to a higher virus infectivity (*β*) in the model. To predict how increases to viral infectivity (*β*) values would impact disease trajectories, we varied (*β*) between 0% and 50% from its original estimated value and assessed predicted outcomes. Although we observed some heterogeneity in response, overall we found that increased infectivity alone is not sufficient to induce significant changes to disease severity (Fig 6), as tissue, cellular, and cytokine concentrations were not dramatically altered from the base case.

### *In silico* knockout investigations predict the impact of immune status on disease outcomes

**Fig 7:**
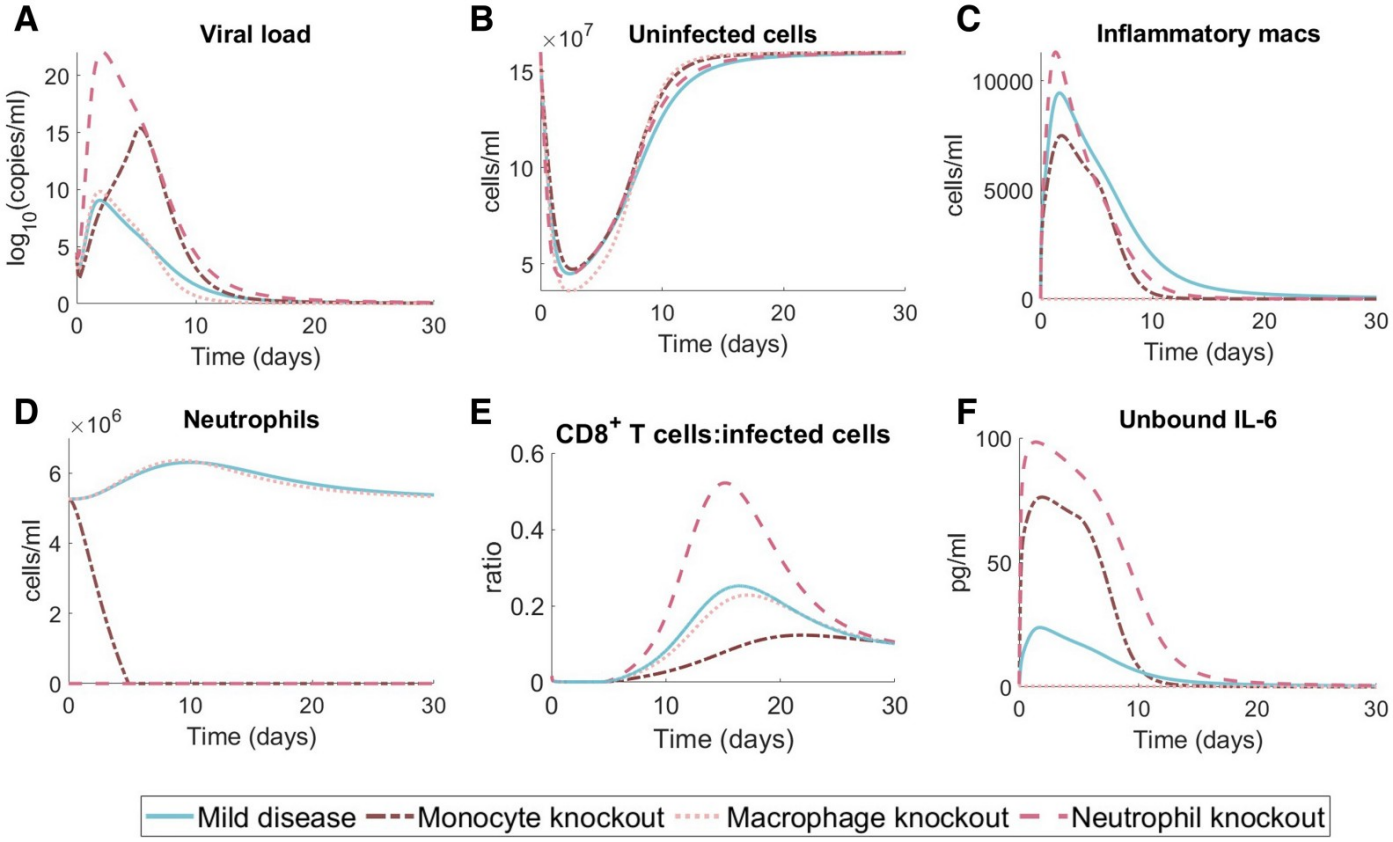
Effects of neutrophil, monocyte, and macrophage knockout on mild disease courses. We performed in silico knockout experiments in the mild disease scenario (Fig 4, blue solid lines) by considering complete monocyte knockout (i.e. no monocyte recruitment and *M*(0) = 0; dark pink dash-dot line), complete macrophage knockout (i.e. not inflammatory macrophage creation via antigen stimulation or monocyte differentiation; light pink dotted line) and complete neutrophil knockout (i.e. no neutrophil recruitment and N(0) = 0; pink dashed line). Dynamics of the in silico knockout are plotted for the **A)** viral load, **B)** uninfected cells, **C)** inflammatory macrophages, **D)** neutrophils, **E)** CD8^+^ T cells relative to uninfected cells and **F)** unbound IL-6.

To better understand the individual contributions of innate immune cell subsets to disease trajectories, we performed a set of *in silico* experiments in which neutrophil, monocyte, or macrophages were completely removed through knockout (Fig 7). For each scenario, we set the initial cell population to zero (i.e. either *N*_0_ = 0, *M*_*Φ*0_ = 0, or *M*_0_ = 0) and also fixed the relevant production rates to be zero (i.e. for the neutrophil knockout $N_{prod}^{*}=\psi_{N}^{max}=p_{N,L}=0$; for the macrophage knockout $a_{I,M\Phi }=p_{M_{\Phi I}}=p_{M_{\Phi I},L}=0$; and for the monocyte knockout $M_{prod}^{*}=\psi_{M}^{max}=p_{M,I}=0$). In the case of neutrophils, the model predicts that completely removing neutrophils leads to large increases in viral loads, IL-6 concentrations, while depressing macrophages. Similarly, monocyte knockout was predicted to result in significant increases to peak IL-6 concentrations compared to mild disease, whereas macrophage knockout resulted in the collapse of neutrophil numbers and extremely suppressed IL-6 concentrations. Overall, the previously found characteristics of disease severity, including low numbers of unaffected lung tissue cells and large populations of inflammatory macrophages, were not exhibited in any of the knockdown *in silico* simulations. Given that there is already systemic dysregulation in the severe case, we chose to focus on the mild disease for the knockout simulations to investigate the impact of isolated dysregulation (knockdown and knockout) on immunological trajectories. A comparison of the knockout experiment to severe disease simulations can be found in S10 Fig.

### Virtual patient cohort identifies heterogeneity in immune dynamics and severity

**Fig 8:**
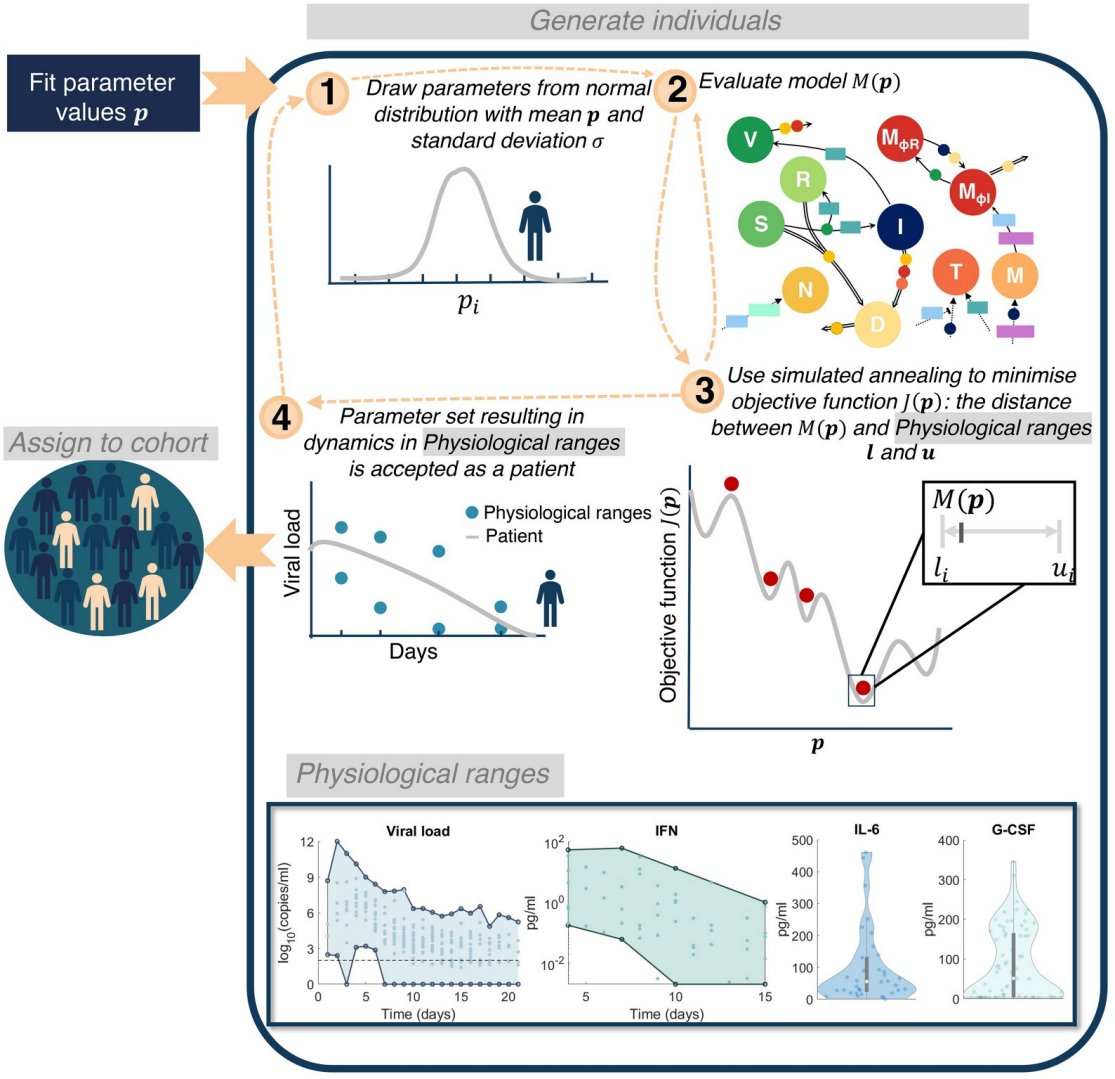
Algorithm for generating virtual patients. Parameters in the model were first obtained through fitting to data (S1 Table). **1)** Parameters relating to macrophage, IL-6 and IFN production ($p_{M\Phi_{I},L}$, *p*_*L*,*MΦ*_, *p*_*F*,*I*_, *p*_*M*,*I*_, *η*_*F*,*MΦ*_, *ϵ*_*F*,*I*_, and *p*_*F*,*M*_) were generated from normal distributions with mean equal to their original fitted values and standard deviation informed by experiment observations (see Section S6.1). **2)** The model evaluated is then simulated on this parameter set to obtain *y*(*t*, *p*). **3)** A simulated annealing algorithm is then used to determine a parameter set that optimises the objective function *J*(*p*) (Eq 17). **4)** Optimizing the objective function provides a parameter set for which the patient response to SARS-CoV-2 will be within the physiological ranges. This patient is then assigned to the cohort and this process is continued until 200 patients have been generated. Physiological ranges are noted in the bottom box for viral load [37], IFN [51], IL-6 [53] and G-CSF [30].

To better understand the clinical variability in SARS-CoV-2 infection severity [1], we next generated a cohort of 200 virtual patients (see Materials and methods and Fig 8). Here, we make the distinction between a “virtual twin” (i.e., a digital representation of a single clinical patient) and a virtual patient/cohort (multiple patients) generated as explained below. To create each *in silico* patient, seven patient-specific parameters were sampled from normal distributions with means corresponding to their respective fixed values and standard deviations inferred from clinical observations (Table 1). In doing this, we assumed intrinsic interindividual heterogeneity in monocyte to macrophage differentiation, production of IL-6 by macrophages, recruitment of monocytes by the presence of infected cells, and production of IFN by infected cells, macrophages and monocytes, respectively.

**Table 1:** Virtual patient-specific parameter values. Seven parameters in the model were deemed patient-specific and were drawn from a normal distribution with mean the parameter value obtained either through fitting or from the literature (S1 Table). The standard deviation (Std Dev) for each normal distribution was informed by values in the literature (see Materials and methods and Supplementary Information Sections S6.1). Initial parameter sampling and new parameters generated through the simulated annealing optimization, were bounded within the interval range noted. All other parameters in the model were fixed to their original value (S1 Table).

Param	Units	Description	Mean	Ref	Std Dev	Ref	Range
$p_{M_{\Phi I},L}$	1/day	Monocyte to macrophage differentiation by IL-6	1.7	[55]	2.2	[7]	[0, 9.9]
$p_{L,M_{\Phi I}}$	pg/ml/day	IL-6 production by inflammatory macrophages	1872	[56]	2.2	[7]	[1863, 1880]
*p*_*F,I*_	pg/ml/day	IFN production by infected cells	2.82	[57]	1.9	[53]	[0, 12.2]
*p*_*M,I*_	1/day	Monocyte recruitment rate by infected cells	0.22	[58]	0.08	[59]	[0, 0.63]
$\eta_{F,M_{\Phi I}}$	10^9^cells/ml	IFN by infected cells	0.0012	[57]	10^−5^	[60]	[0, 4.7 × 10^−4^]
*ϵ*_*F,I*_	pg/ml	IFN production of CD8^+^ T cells	0.004	[61]	10^−5^	[54]	[0, 5.34 × 10^−4^]
*p*_*F,M*_	pg/ml/day	IFN production by monocytes	3.56	[62,63]	0.013	[62]	[3.4, 3.6]

Parameters were chosen based on their impact on maximum IL-6 and IFN levels as well as tissue damage observed in the sensitivity analysis ($p_{M\Phi_{I},L}$, *p*_*L*,*M*Φ_, *p*_*F*,*I*_, *p*_*M*,*I*_, and *ϵ*_*F*,*I*_; Fig 5). In addition, we designated patient-specific parameters accounting for alternate pathways through which IFN is affected by innate immune cells (*η*_*F*,*M*Φ_ and *p*_*F*,*M*_). For the production of IL-6 by macrophages and monocyte to macrophage differentiation via IL-6 stimulation, standard deviations were inferred from IL-6 levels in non-mechanically ventilated patients (mild) and from mechanically ventilated patients (severe) [53] (S7D Fig). Standard deviations for the production of IFN by infected cells were determined from the 95% confidence interval for IFN-*α* from Trouillet-Assant et al. [51] (S7A and S7B Fig), and, lastly, the standard deviation for the production of IFN by macrophages was obtained from the 95% confidence interval in Sheahan et al. [54] (S7C Fig). The variation in virtual patient responses was then constrained by experimental and clinical viral loads, IFN, neutrophil, IL-6, and G-CSF (Fig 8). The resulting cohort dynamics were within ranges for IFN and IL-6 measurements in asymptomatic to severe COVID-19 patients in the literature [11,17] (S11 Fig).

**Fig 9:**
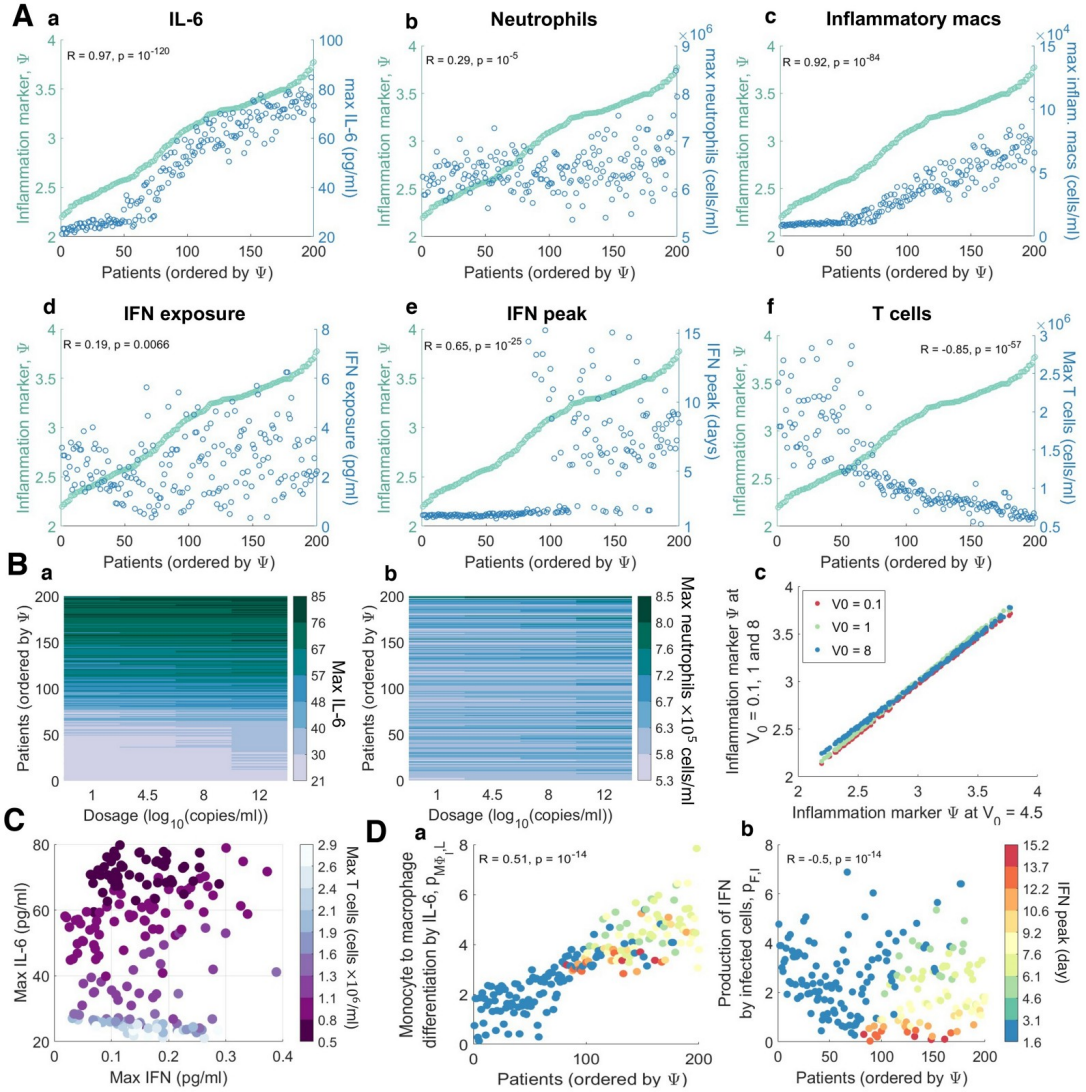
Virtual cohort of SARS-CoV-2 infected patients. 200 virtual patients were generated by sampling parameters related to macrophage, IL-6, and IFN production ($p_{M\Phi_{I},L}$, *p*_*L*,*MΦ*_, *p*_*F*,*I*_, *p*_*M*,*I*_, *η*_*F*,*MΦ*_, *ϵ*_*F*,*I*_, and *p*_*F*,*M*_) from normal distributions with mean equal to their original values and standard deviation inferred from clinical observations (Fig 8). Each virtual patient had a distinct parameter set optimized to that patient’s dynamics in response to SARS-CoV-2 infection which corresponded to physiological intervals reported in the literature (see Materials and methods). **A)** Infection and immune response metrics (blue) in individual patients were compared to inflammatory variable Ψ (green). Each point represents an individual patient, ordered according to Ψ. The correlation coefficient (R) and p-value are indicated for each, with α<0.05 denoting significant correlations. **B)** The effect of exposure dose *V*_0_ on maximum IL-6 (a), maximum neutrophil counts (b) and inflammation marker Ψ (c) for *V*_0_ = 0.1, 1, 4.5 and 8 log_10_(*copies*/*ml*). In a and b, rows are coloured according to each virtual patient’s inflammation marker value; virtual patients were ordered by the value of Ψ from the baseline scenario in **A** (*V*_0_ = 4.5 log_10_(*copies*/*ml*)). **C)** Correlations between maximal IFN, IL-6, and T cell concentrations for each patient (circles). Circle colours correspond to the maximal T cell concentration of each patient. **D)** Parameters most correlated to the IFN peak time were the rates of macrophage production via a) IL-6 ($p_{M\Phi_{I},L}$) and the b) IFN production by infected cells (*p*_*F*,*I*_). Individual patient values for these parameters are plotted as circles coloured by the patient’s corresponding day of IFN peak (see color bar). Patients were ordered by their inflammation marker Ψ.

To quantify disease severity, we introduced an inflammation variable, Ψ, that measured maximum IL-6, neutrophils and tissue damage (Eq 18) and then compared it to individual characteristics of each virtual patient’s disease. We evaluated each virtual patient’s maximum IL-6, CD8^+^ T cells, and neutrophils; minimum percentage of healthy lung tissue; the time to peak IFN; and total IFN exposure (area under the curve or AUC) within 21 days of infection. Ordering patients by their value of Ψ and plotting the corresponding values for different characteristics showed a clear separation between those with mild disease (Ψ ≤ 3) and those with severe disease (Ψ > 3) (Fig 9A).

In these virtual patients, we investigated predicted disease severity over a range of initial virus exposure doses (*V*_0_ = 0.1, 1, 4.5, 8). By comparing outcomes with respect to several immunological biomarkers (e.g. peak IL-6 concentrations, time to IFN peak etc.), we found that virtual patients who were predicted to experience mild COVID-19 in our base case (exposure dose of 4.5 log_10_ viral copies/mL) experienced mild disease irrespective of the exposure dose size (Fig 9B). In particular, those predisposed to experience severe disease were predicted to experience poor outcomes regardless of inoculation (Fig 9Bc). We observed that patients that had severe disease responses for an inoculation of *V*_0_ = 4.5 log_10_(copies/mL) (i.e. patients 150 to 200) had little to no change in their IFN, IL-6 or neutrophil dynamics under changing inoculation size (Fig 9Ba and 9Bb). However, patients who were prone to have less severe disease responses (i.e. patients 1 to 100) had larger variations in their immune dynamics, in particular IL-6 (Fig 9Ba).

Our model further predicted that patients with higher inflammation had higher IL-6, neutrophil, and inflammatory macrophage concentrations (Fig 9Aa–9Ac), which is somewhat to be expected given that IL-6 is a component of the inflammatory marker Ψ. While the IFN exposure was not significantly stratified by Ψ (Fig 9Ad), the peak of IFN (Fig 9Ae) and CD8^+^ T cell levels (Fig 9Af) were strongly negatively correlated with the inflammation marker (R = −0.85, p < 1 × 10^−9^, see Materials and methods). IL-6 was most noticeably correlated with Ψ (R = 0.91, p<1 × 10^−9^), with a distinct upper bound in the concentration (~80 pg/ml) achieved in 50% of the virtual cohort (Fig 9Aa). There appeared to be a transition phase in inflammation driven by inflammatory macrophage levels where patients with mild inflammation (Ψ < 2.5) had low counts (less than 4 × 10^4^ cells/ml) compared to patients with more severe inflammation (Ψ ≥ 2.5) who had higher levels (p = 1.46 × 10^−6^; Fig 9Ac). Despite this, patients with moderate inflammation exhibited increased disease markers including delayed IFN peaks and lower CD8^+^ T cells, compared to patients with mild inflammation (Ψ ≤ 2.5).

A distinct jump in the timing of the IFN peak in the virtual cohort (p <1 × 10^−5^) was found to be correlated with inflammation (Fig 9Ae), as patients with lower (mild) inflammation (Ψ ≤ 2.5) had peaks at day 2 compared to day 6 in patients with higher (severe) inflammation (Ψ>2.5). Grouping virtual individuals by their time to IFN peak suggests that those with IFN peaks before day 3 of infection also had fewer macrophages (p<1 × 10^−5^) and larger numbers of CD8^+^ T cells (p <1 × 10^−5^). Overall, delays in IFN peak did not cause significant changes to viral load but were sufficient to cause major tissue damage (100x reduction in viable tissue remaining) and over-heightened immune responses (4x increase in maximum IL-6 and GM-CSF concentrations).

Further, examining the relationship between each virtual patient’s maximum IL-6, IFN, and CD8^+^ T cell concentrations (Fig 9C) identified a weaker correlation between the maximum concentration of CD8^+^ T cells and IFN (R = 0.24, p = 0.0008) as opposed to with IL-6 (R = −0.86, p < 1 × 10^−9^). As expected, we also found a positive correlation (R = 0.67, p = 1.58 × 10^−8^) between the time to peak IFN concentration for each patient and the IFN production rate from infected cells (Fig 9Db). Interestingly, the time to peak IFN for each patient was also strongly related to their rate of IL-6-stimulated monocyte differentiation into macrophages (Fig 9Da). Low IFN production rates were predicted to be the major factor responsible for significantly delayed IFN peaks over 6 days after infection, whereas IFN peaks within 3 days of infection were largely caused by lower rates of monocyte to macrophage differentiation (Fig 9Da).

## Discussion

Serial immunological measurements from COVID-19 patients are only beginning to be collected, and the ability to assess initial infection kinetics and the drivers of the ensuing disease course remains limited. The data-driven mechanistic mathematical model and virtual patient cohort developed here is an important platform contributing to investigating immunological drivers of COVID-19. In particular, to recreate severe dynamics, it was sufficient to vary only two processes in the model: the rates of type I IFN production from infected cells and macrophages, and the rate of monocyte recruitment by infected cells. This suggests that the distinction between severe and mild disease may be driven by a limited set of causal regulators that warrant subsequent study. The effect on IFN production may be further exacerbated by autoimmunity against type I IFNs, which has been shown to correlate to life-threatening COVID-19 pneumonia in 2.6% of women and 12.5% of men [18].

Our results show that delaying type I IFN production is sufficient to cause major tissue damage and heightened immune responses, yet it has little impact on peak viral loads. In the severe disease simulation, the viral load was cleared marginally faster (~1 day) in comparison to the mild disease simulation. This finding is supported by recent clinical evidence suggesting that the rate of viral decline may be predictive of disease severity [6,64]. This therefore suggests that viral loads alone may not be a necessary attribute to obtain severe tissue damage. Instead, our model predicts that increases in tissue damage occur through heightened innate immune responses. Further, increases to the viral infectivity rate β, which replicate changes in ACE2 expression between individuals and/or viral mutations causing increases in infectivity, were not predicted to significantly change disease trajectories. This observation is consistent with early SARS-CoV-2 variants, particularly D614G, where a spike protein mutation increased infectivity but did not significantly alter disease outcomes [65] (similar conclusions are emerging for the alpha (B.1.1.7) variant [66,67]). As noted in Table 2, these results suggest future experiments using mutated viral variants, and a separate mechanistic modelling study examining a gradient of ACE2 expression along the respiratory tract and/or within organ systems to understand how within-host variation in ACE2 receptor expression impacts heterogeneity in the same virus. Future work will explore the potential immunopathological effects of newer variants of concern.

**Table 2:** Summary of model hypotheses, effects, possible experiments to test each hypothesis, and available experimental and/or clinical evidence in agreement with prediction.

Effect/metric	Predicted effect(s) on disease heterogeneity	Hypothesis generated	Possible experiments	Experimental and/or clinical evidence
Viral dose at exposure	COVID-19 severity largely dependent on an individual’s propensity for severe disease	Heterogeneity in viral loads and immunopathology in COVID-19 are determined by patient-intrinsic immune responses	Inoculation escalation studies in animal model measuring lung histology, weight loss, and longitudinal cytokine profiles pre- and post-infection	--
Viral infectivity rate (β)	Minimal	Small increases in viral infectivity alone are not drivers of COVID-19 severity	Measure disease outcomes in animal model after infection by viral variants with point mutations in spike protein (e.g. D614G)	D614G mutation increases infectivity but not COVID-19 severity [65,70]
IFN exposure (area under the IFN time-curve)	No association	IFN timing is more crucial to disease severity than exposure	Comparison of outcomes with and without IFN receptor inhibition; treatment with IFN initiated at varying points after infection (measure: lung histology, weight loss, longitudinal cytokine profiling, differential gene expression)	Known for SARS-CoV and MERS [13,14]; early IFN therapy associated with better responses (retrospective cohort study) [28]
Time to IFN peak concentrations	High correlation	Impaired/slow monocyte-to-macrophage differentiation rates in severe cases	Evaluate longitudinal gene expression and cell dynamics of monocyte and dendritic cell subsets after infection using RNAseq and flow cytometry	--
Time to IFN peak concentrations	High correlation	IFN production is dysregulated/impaired in severe cases	Kinetic experiments measuring IFN expression after infection	Anti-IFN mechanisms in SARS-CoV-2 [71,72]; weak induction of IFN signaling by SARS-CoV-2 [73]

Neutrophil and monocyte knockout simulations suggested that excessive suppression of neutrophils early in infection may lead to future hyperinflammation even in otherwise mild COVID-19. The peak ratio between CD8^+^ T cells and infected cells remained virtually unchanged across the neutrophil, monocyte, and macrophage knockouts we considered, which is indicative of the link between IFN, infected cells, and CD8^+^ T cells in our model.

Evaluating SARS-CoV-2 infection in a cohort of 200 virtual patients revealed several immunological responses potentially leading to differential disease presentation. Notably, a distinct, emergent switch in the type I IFN response corresponded with late IFN peaks and more severe disease (i.e., higher inflammation Ψ). This supports previous findings that connect a delay in type I IFN with more severe presentations of highly pathogenic coronaviruses infections including SARS-CoV, MERS-CoV, and SARS-CoV-2 [10,13,14], and provides a rational explanation for the finding from a retrospective cohort study that early IFN therapy is associated with better responses [28]. Of note, varying the initial viral inoculum did not impact disease outcomes in virtual patients predicted to have either mild or severe disease. These results seem to suggest that it is an individual’s intrinsic immunological response that dictates the severity of COVID-19. Further, the inflammation marker Ψ can also be applied to patient data, similar to other proposed metrics (e.g. CytoScore [68]).

In our cohort, virtual patients with mild disease tended to achieve peak IFN concentrations approximately 2 days after infection compared to those with severe disease who exhibited higher inflammation and later IFN responses peaking after 5 days. This switch in IFN timing was caused by a 3-fold increase in the rate of monocyte-to-inflammatory macrophage differentiation and decreased production rate of IFN by infected cells. The initial delay of IFN production was caused by increased monocyte-to-macrophage differentiation and this delay was exacerbated by reduced IFN production from infected cells, suggesting that the timing of the IFN peak in a patient may allow for improved stratification into treatment arms designed to target one or both of these responses. The finding that IFN binding was predictive of the duration of lung tissue damage suggests that virus-intrinsic properties and their ability to inhibit receptor mediated binding and endocytosis could delay IFN production and cause downstream increases in IL-6 and GM-CSF, resulting in severe disease. Our results further suggest that lymphopenia is tightly correlated with maximum IL-6 concentration and less dependent on the timing of IFN.

Models by design require simplifying complex dynamics to highlight critical underlying structures, which should then be experimentally or clinically verified. For instance, some unmodeled cytokines have overlapping function and cellular sources to those explicitly modelled here; thus, model predictions may be representative of broader synergistic effects [68] and other cytokines could also be targets of interest. Also, as this work is focused on acute, primary infections, our model does not account for antibody production and we chose not to explore questions related to vaccination efforts nor the potential for cross-reactivity given exposure to other seasonal coronaviruses. Interestingly, recent evidence suggests that antibodies to other seasonal coronaviruses do increase in SARS-CoV-2 but that this does not provide protection [69]. Extending this model in the future to incorporate antibody production and addressing persistent or secondary infection could help shed light on questions about vaccine escape. There are also certain limitations to our virtual cohort simulations. As the true variation of these parameters in humans is unknown and in some cases impossible to measure, we used variation in related data sets for their kinetics. As such, the virtual cohort represents a prognostic guide, and future experiments would be needed to validate the findings in human patients before formal conclusions can be drawn.

Importantly, our approach suggests future avenues of experimental studies through a hypothesis-generation and prediction paradigm, suggesting key mechanisms as promising avenues of investigation (Table 2).

Indeed, the ability of our model to recapitulate severe disease by, in part, regulating monocyte differentiation raises the possibility that patients with low monocyte levels [7] may benefit from treatments that better regulate monocyte differentiation. This is in line with recent studies identifying distinct transcriptional factors as regulators of differentiated monocyte fates in inflammatory conditions [74,75], and clinical observations that monocyte dysregulation is present in severe COVID-19 [76,77]. It also raises the possibility that modulation by exogenous cytokines, including macrophage colony-stimulating factor in combination with IL-4 and tumour necrosis factor-alpha (TNF-α), may be able to direct monocyte differentiation in favour of monocyte-derived dendritic cells and reduce this response [74]. Recently, the neutralization of both TNF-α and IFN-γ has been found to benefit patients with COVID-19 or other cytokine storm-drive syndromes by limiting inflammation and tissue damage [78]. Given that TNF-α also has a secondary benefit on monocyte differentiation, our results support the viability of this avenue of treatment. Caution should be noted, however, given that some previous attempts to regulate host responses by IL-6 blockade have proven unsuccessful [79]. Together, our findings support the idea that early interventions aimed at reducing inflammation are more likely to be beneficial for patients at risk of progressing to severe COVID-19 than attempts to inhibit cytokine storm later in the disease course, given that early IFN responses were found to provoke better controlled immune responses and outcomes in our virtual cohort. It will be essential to characterize both the timing and mechanisms of proposed therapeutic interventions to develop effective treatments to mitigate severe disease.

## Materials and methods

### Mathematical model of the immune response to SARS-CoV-2

Our model (**Eqs. S1-S22**) was developed to examine heterogeneous SARS-CoV-2 infection dynamics and explore the immunological drivers of disease severity. The complete model is provided in the Supplementary Information Section S1 along with all variable and parameter descriptions provided in S1 Table, Section S2. Throughout, cytokine and immune cell interactions and effects were described by Hill functions as

$$\frac{B^{h}}{B^{h}+\gamma^{h}},$$ (1)

where *B* is the interacting compound, *γ* its half-effect value, and *h* the Hill coefficient [80,81]. Further, for a given cytokine *X* and cell population *Y*, the production (recruitment/differentiation) rate of *X* by *Y* was denoted by *p*_*X*,*Y*_ and the rate of production of *Y* by *X* by *p*_*Y*,*X*_. The half-effect concentration (i.e. *γ* in Eq 1) of cytokine *X* on cell population *Y* was represented by *ϵ*_*X*,*Y*_ and the half-effect concentration of cell *Y* affecting cytokine *X* was given by *η*_*X*,*Y*_. The natural death rate of cell *Y* was denoted by *d*_*Y*_, and the rate of induced death of cell *Y* by cell *Z* by *δ*_*Y*,*Z*_. Lastly, the carrying capacity concentration of cell *Y* was denoted by *Y*_*max*_, and regeneration or proliferation rates by *λ*_*Y*_.

We modelled virus (*V*) as produced by infected cells at rate *p* and cleared via exponential clearance at rate *d*_*V*_, which accounts for all contributions to viral degradation except macrophage- and neutrophil-mediated clearance. Immune-mediated viral clearance via phagocytosis by inflammatory macrophages [82] and neutrophil extracellular traps (NETs—extracellular chromatin fibres produced by neutrophils to control infections) [45,46] was considered to occur at rates $\delta_{V,M_{\Phi I}}$ and *δ*_*V*,*N*_, respectively. Susceptible epithelial cells (*S*) grow logistically with per capita proliferation rate *λ*_*S*_ and carrying capacity *S*_*max*_, and become infected (*I*) at rate *β*. The damage inflicted on epithelial cells by neutrophils was modelled using a Hill function (Eq 1) [81], where neutrophils kill/damage epithelial cells at rate *δ*_*N*_ through the release of NETs and other antimicrobials proteins [45,46]. The constant *ρ* (0 < *ρ* < 1) was included to modulate bystander damage of uninfected cells (*S* and *R*).

For the purposes of our study, we only considered type I IFN dynamics (primarily IFN-α, β). Type I IFN (*F*_*U*_ and *F*_*B*_) reduces the infectivity and replication capability of viruses by stimulating cells to become resistant to infection [22]. These resistant cells (*R*) proliferate at a rate equivalent to susceptible cells (*λ*_*S*_). The concentration of bound IFN (*F*_*B*_) modulates the creation of infected and resistant cells [19,21,83,84], where increasing the concentration of IFN causes more cells to become resistant to infection and less to become productively infected (*I*). The potency of this effect is controlled by the half-effect parameter *ϵ*_*F*,*I*_. Following the eclipse phase (which lasts *τ*_*I*_ hours), productively infected cells (*I*) were modelled to produce virus before undergoing virus-mediated lysis at rate *d*_*I*_. Although various immune cell subsets contribute to infected cell clearance, we limited our investigation to macrophages and effector CD8^+^ T cells which induce apoptosis at rates *δ*_*I*,*M*Φ_ and *δ*_*I*,*T*_, respectively.

The accumulation of dead cells (*D*) was assumed to occur through infected cell lysis *d*_*I*_, neutrophil damage/killing of epithelial cells *δ*_*N*_, macrophage phagocytosis of infected cells *δ*_*I*,*M*Φ_, macrophage exhaustion *δ*_*M*Φ,*D*_, and CD8^+^ T cell killing of infected cells *δ*_*I*,*T*_. These dead cells disintegrate relatively quickly [85] at rate *d*_*D*_, and are cleared through phagocytosis by macrophages [86] at rate *δ*_*D*,*M*Φ_.

Resident alveolar macrophages (*δ*_Φ*R*_) are considered to be replenished at a logistic rate inversely proportion to viral load with maximal rate of *λ*_*M*Φ_ and half-effect *ϵ*_*V*,*M*Φ_ (i.e. as the virus is cleared, the inflammatory macrophage pool replenishes the alveolar macrophage population in the lung). We modelled the transition of alveolar macrophages to inflammatory macrophages (*M*_Φ*I*_) as dependent on infected and dead cells, with a maximal rate of *a*_*I*,*M*Φ_. Resident macrophages die naturally at a rate $d_{M\Phi_{R}}$ or due to the clearing of dead cells (exhaustion) [86] at rate *δ*_*M*Φ,*D*_.

Inflammatory macrophages were modelled as produced by three distinct pathways (acting individually or in concert): 1) stimulated tissue-resident macrophages *a*_*I*,*M*Φ_, (2) GM-CSF-dependent monocyte differentiation, with maximal production *p*_*M*,*G*_ and half effect *ϵ*_*G*,*M*_, and (3) IL-6-dependent monocyte differentiation, with maximal production rate $p_{M_{\Phi I,L}}$ and half-effect $\epsilon_{L,M_{\Phi I}}$. We assumed that inflammatory macrophages die naturally at rate $d_{M_{\Phi I}}$ or from clearing dead cells at a rate *δ*_*M*Φ,*D*_.

We have previously shown that endogenous cytokine concentrations are not at quasi-equilibrium at homeostasis [87]. Therefore, to describe the pharmacokinetics and pharmacodynamics of cytokine binding and unbinding, we leveraged the framework established in Craig et al. [87] (Fig 1C) for IFN (*F*_*B*_ and *F*_*U*_), IL-6 (*L*_*B*_ and *L*_*U*_), GM-CSF (*G*_*B*_ and *G*_*U*_), and G-CSF (*C*_*B*_ and *C*_*U*_). In its general form, this pharmacokinetic relationship is expressed as

$$\frac{dY_{U}}{dt}=Y_{prod}-k_{lin}Y_{U}-k_{B}\left(XA-Y_{B}\right){\left(Y_{U}\right)}^{POW}+k_{U}Y_{B},$$ (2)

$$\frac{dY_{B}}{dt}=\;-k_{int}Y_{B}+k_{B}\left(XA-Y_{B}\right){\left(Y_{U}\right)}^{POW}-k_{U}Y_{B}$$ (3)

where *Y*_*U*_ and *Y*_*B*_ are free and bound cytokines, *Y*_*prod*_ is the rate of endogenous cytokine production, *k*_*B*_ and *k*_*U*_ are the respective binding and unbinding rates, *k*_*int*_ is the internalization rate of bound cytokine, and *k*_*lin*_ is the elimination rate. Here, *POW* is a stoichiometric constant, *A* is a scaling factor and *X* is the sum of all cells modulated by the cytokine with

$$XA=\hat{p}Y_{MW}K10^{n}X.$$ (4)

where $\hat{p}$ is a constant relating the stoichiometry between cytokine molecules and their receptors, *K* is the number of receptors specific to each cytokine on a cell’s surface and 10^*n*^ is a factor correcting for cellular units (see **Eqs. S19-S22**). The molecular weight was calculated in the standard way by dividing the cytokine’s molar mass (*MM*) by Avogadro’s number (*Y*_*MW*_ = MM/6.02214 × 10^23^).

We considered unbound IL-6 (*L*_*U*_) to be produced from productively infected cells, inflammatory macrophages, and monocytes, with bound IL-6 (*L*_*B*_) resulting from binding to receptors on the surface of neutrophils, CD8^+^ T cells and monocytes. Unbound GM-CSF (*G*_*U*_) was assumed to be produced from inflammatory macrophages and monocytes and bind to receptors on monocytes to create bound GM-CSF (*G*_*B*_). GM-CSF can be produced by CD8^+^ T cells [88], but this was excluded because it was insignificant to the full system’s dynamics. Unbound G-CSF (*C*_*U*_) is secreted by monocytes, with bound G-CSF (*C*_*B*_) produced via binding to neutrophil receptors. Lastly, because unbound type I IFNs (*F*_*U*_) are known to be produced by multiple cell types in response to viral infection, including lymphocytes, macrophages, endothelial cells and fibroblasts [83], we modelled its unbound production from infected cells, infiltrating/inflammatory macrophages, and monocytes, and its binding to receptors on both CD8^+^ T cells and infected cells (Fig 1B).

The pharmacokinetics and pharmacodynamics of G-CSF on neutrophils (*N*) were taken directly from Craig et al. [87]:

$$\frac{dN}{dt}=\left(N_{prod}^{*}+\left(\psi_{N}^{max}-N_{prod}^{*}\right)\frac{C_{BF}-C_{BF}^{*}}{C_{BF}-C_{BF}^{*}+\epsilon_{C,N}}\right)N_{R}.$$ (5)

Neutrophil recruitment of bone marrow reservoir neutrophils (*N*_*R*_) was modelled to occur via the bound fraction of G-CSF [89] (*C*_*BF*_ = *C*_*B*_(*t*)/(*A*_*C*_*N*(*t*))) at rate $N_{prod}^{*}$ which increases towards its maximal value $\psi_{N}^{max}$ as a function of increasing G-CSF. During the acute phase of inflammation, endothelial cells produce IL-6 leading to the attraction of neutrophils [90]. This was modelled as recruitment with maximal rate *p*_*N*,*L*_ and half-effect parameter *ϵ*_*D*,*L*_. Neutrophils die at rate *d*_*N*_.

Monocytes (*M*) are recruited by bound GM-CSF [91], similar to neutrophils (Eq 5), with bone marrow monocytes (*M*_*R*_) recruited at a homeostatic rate $M_{prod}^{*}$. In the presence of GM-CSF, this rate increases towards $\psi_{M}^{max}$. Monocytes are also recruited by the presence of infected cells at a maximal rate of *p*_*M*,*I*_ with half-effect *ϵ*_*I*,*M*_, and subsequently disappear through differentiation into inflammatory macrophages (as above) or death at rate *d*_*M*_.

CD8^+^ T cells are recruited through antigen presentation on infected cells as a function of infected cell numbers at rate *p*_*T*,*I*_ The constant delay (*τ*_*T*_) accounts for the time taken for dendritic cells to activate, migrate to the lymph nodes, activate CD8^+^ T cells, and the arrival of effector CD8^+^ T cells at the infection site. CD8^+^ T cell expansion occurs in response to bound IFN at a maximal rate *p*_*T*,*F*_ with half-effect *ϵ*_*F*,*T*_, and CD8^+^ T-cell exhaustion occurs with high concentrations of IL-6 [16,17], with half-effect *ϵ*_*L*,*T*_, and apoptosis occurs at rate *d*_*T*_.

### Estimating early infection dynamics (‘viral model’)

In an attempt to reduce the degrees of freedom during parameter estimation, we deployed a step-wise approach by isolating subsections of the model. This approach helps mitigate potential issues with parameter identifiability, given that ours is a large, nonlinear model [92–94] and allows us to estimate parameters from multiple data sources [87,95,96]. Other methodologies, including Bayesian computation, are alternative approaches in this context. To begin estimating parameter values from data, we set all immune populations and cytokine concentrations in the full model (Supplementary Information **Eqs. S1-S22**) to zero (*M*_Φ*R*_ = *M*_Φ*I*_ = *M* = *N* = *T* = *L*_*U*_ = *L*_*B*_ = *G*_*U*_ = *G*_*B*_ = *C*_*U*_ = *C*_*B*_ = *F*_*U*_ = *F*_*B*_ = 0). This gives

$$\frac{dV}{dt}=pI-d_{V}V,$$ (6)

$$\frac{dS}{dt}=\lambda_{S}\left(1-\frac{S+I+D}{S_{max}}\right)S-\beta SV,$$ (7)

$$\frac{dI}{dt}=\beta S\left(t-\tau_{I}\right)V\left(t-\tau_{I}\right)-d_{I}I,$$ (8)

$$\frac{dD}{dt}=d_{I}I-d_{D}D.$$ (9)

We also assumed there were no resistant cells (*R* = 0) due to the absence of an IFN equation. This resulted in a simplified ‘viral model’ that considers only virus (*V*) infection of susceptible cells (*S*) which creates infected cells (*I*) after *τ*_*I*_ days, which the die through lysis, creating dead cells (*D*).

### Type I interferon dynamics during early infection (‘IFN model’)

To study infection dynamics driven uniquely by IFN, we extended Eqs 6–9 by introducing the IFN mechanisms from **Eqs. S1-S22**, i.e. setting other cytokine and immune cell populations to zero (*M*_Φ*R*_ = *M*_Φ*I*_ = *M* = *N* = *T* = *L*_*U*_ = *L*_*B*_ = *G*_*U*_ = *G*_*B*_ = *C*_*U*_ = *C*_*B*_ = 0), giving

$$\frac{dV}{dt}=pI-d_{V}V,$$ (10)

$$\frac{dS}{dt}=\lambda_{S}\left(1-\frac{S+I+R+D}{S_{max}}\right)S-\beta SV,$$ (11)

$$\frac{dI}{dt}=\frac{\beta S\left(t-\tau_{I}\right)V\left(t-\tau_{I}\right)\epsilon_{F,I}}{\epsilon_{F,I}+F_{B}}-d_{I}I,$$ (12)

$$\frac{dR}{dt}=\lambda_{S}\left(1-\frac{S+I+R+D}{S_{max}}\right)R+\frac{\beta S\left(t-\tau_{I}\right)V\left(t-\tau_{I}\right)F_{B}}{F_{B}+\epsilon_{F,I}},$$ (13)

$$\frac{dD}{dt}=d_{I}I-d_{D}D,$$ (14)

$$\frac{dF_{U}}{dt}=\psi_{F}^{prod}+\frac{p_{F,I}I}{I+\eta_{F,I}}-k_{lin_{F}}F_{U}-k_{B_{F}}\left(\left(T^{*}+I\right)A_{F}-F_{B}\right)F_{U}+k_{U_{F}}F_{B},$$ (15)

$$\frac{dF_{B}}{dt}=-k_{int_{F}}F_{B}+k_{B_{F}}\left(\left(T^{*}+I\right)A_{F}-F_{B}\right)F_{U}-k_{U_{F}}F_{B},$$ (16)

where cells become resistant (*R*) through IFN (*F*_*U*_ and *F*_*B*_). The parameter $\psi_{F}^{prod}$ was introduced to account for the production of IFN by macrophages and monocytes not explicitly modelled in this reduced system but included in the full system (i.e. *p*_*F*,*M*_ and *p*_*F*,*M*Φ_ in **Eq. S17**). Previously-fit parameters were then fixed to their estimated values (S1 Table) and the value of $\psi_{F}^{prod}$ was determined by solving *dF*_*U*_/*d*_*t*_ = 0 at homeostasis (i.e. *V* = *I* = 0), giving $\psi_{F}^{prod}=0.25$.

### Model calibration and parameter estimation

Model parameters (S1 Table) were obtained either directly from the literature, using the half-life formula (**Eq. S23**), through fitting effect curves (**Eqs. S24-S25**) or sub-models (**Eqs. S26-S56**) to *in vitro*, *in vivo*, and clinical data, or by calculating the value that ensured that homeostasis was maintained (**Eqs. S57-S70**) in the absence of infection. All fitting procedures were performed using MATLAB 2019b functions *fmincon* or *lsqnonlin* [97]. Full details are given in the Supplementary Information, and a brief summary is provided below.

Initial concentrations of all unbound cytokines (*L*_*U*,0_, *G*_*U*,0_, *C*_*U*,0_ and *F*_*U*,0_), susceptible cells, resident macrophages, monocytes, neutrophils, and CD8^+^ T cells (*S*_0_, *M*_Φ*R*,0_, *M*_0_, *N*_0_ and *T*_0_) were estimated from plasma and lung tissue concentrations in humans [87,98–104] (Section S3.1-S3.2). Parameters for cytokine binding and unbinding kinetics (Eqs 2–4), such as the molecular weight (*MM*), binding sites per cell (*K*), binding/unbinding rates (*k*_*B*_ and *k*_*U*_), internalization rates for GM-CSF, G-CSF and IFN (*k*_*int*_), and cytokine clearance rates (*k*_*lin*_), were estimated both from known values in the literature [84,87,105–115] and previous modelling work [87,116,117] (Section S3.3-S3.5). The stoichiometric constants *POW* and $\hat{p}$ were both equal to 1 for all cytokines, except for G-CSF for which *POW* = 1.4608 and $\hat{p}=2$ as previously estimated by Craig et al. [87]. Neutrophil and monocyte reservoir dynamics, monocyte differentiation, macrophage activation, and CD8^+^ T cell recruitment and expansion parameters were primarily estimated from previous mathematical modelling studies [87,118] as well as known values [55,58,81,119] (Section S3.6-S3.8). Immune cell death rates were taken directly from the literature [59,95,120] or estimated from recorded half-lives [60,85,86,121,122] using **Eq. S23** (Section S3.9).

To estimate the rates of virus production, decay, infectivity, and infected cell lysis (*p*, *d*_*V*_, *β* and *d*_*I*_ respectively) in early infections, we initially fit Eqs 6–9 to viral load measurements from SARS-CoV-2 infection in macaques [47] where eight adult rhesus macaques inoculated with 4 × 10^5^ TCID_50_/ml (3 × 10^8^ genome copies/ml) SARS-CoV-2 [47] (S6 Fig). These parameter values then informed our estimations from hospitalized individuals (Fig 2). We used two data sets of SARS-CoV-2 shedding in the absence of effective treatment from patients in Singapore [40] (n = 3) and patients in Germany [49] (n = 5). In Singapore, samples were obtained with nasopharyngeal swabs whereas viral loads were measured directly from sputum in Germany.

Given the heterogeneity in viral loads from human patients (since available human SARS-CoV-2 viral load data is generally measured from the day of onset of symptoms or after hospitalization without knowing the viral exposure size), Goyal et al. [37] estimated the lag between initial inoculation and first viral measurement for each patient. We used their estimates of the lag and viral production rate *p*, as well as the estimates for *d*_*V*_, *β*, and *d*_*I*_ from fitting the macaque date (S6 Fig) to estimate the viral parameters *d*_*V*_, *β* and *d*_*I*_ for the human SARS-CoV-2 viral load data. Viral loads below 2 log_10_(copy/ml) were assumed to be negligible [37]. Estimated parameters for viral decay (*d*_*V*_) and cell lysis (*d*_*I*_) were used as an upper bound for parameter values in the full model to account for additional viral clearance and cell killing of the immune system.

A subset of parameters was obtained through fitting sigmoidal effect curves (**Eqs. S24-S25**) curves to *in vitro* and *in vivo* experiments. These include the IFN inhibition of viral infection and replication [54] (*ϵ*_*F*,*I*_; Section S4.1.1), the half-effect neutrophil concentration for epithelial cell damage [123] (*IC*_50,*N*_; Section S4.1.2), and the half-effect concentrations for monocyte production and differentiation through GM-CSF signalling [124] (*ϵ*_*G*,*M*_ and $\epsilon_{G,M\Phi_{I}}$; Section S4.1.3) see S1 Fig. Other parameters obtained through effect curves were the half-effects for IL-6 production by monocytes [125] and the effect of IL-6 on monocyte differentiation [44] (*η*_*L*,*M*_ and *ϵ*_*L*,*M*_; Section S4.1.4), and the half-effect of IFN on CD8^+^ T cell [61] (*ϵ*_*F*,*T*_; Section S4.1.5) and IL-6 on CD8^+^ T cell expansion [126] (*ϵ*_*L*,*T*_; Section S4.1.6; S2 Fig).

These parameters were then fixed, and remaining parameters were estimated by fitting time-dependent sub-models of **Eqs. S1-S22** to relevant data. The proliferation rate of epithelial cells (*λ*_*S*_; Section S4.2.1), the internalization rate of IL-6 ($k_{int_{L}}$; Section S4.2.2), and the rate of neutrophil induced damage (*δ*_*N*_; Section S4.2.3) were fit to corresponding time-series measurements [127–129] using exponential rate terms (S2 Fig). Clearance and phagocytosis of infected cells and extracellular virus by inflammatory macrophages (*δ*_*I*,*M*Φ_ and *δ*_*V*,*M*Φ_; Section S4.2.4-S4.2.5) were fit to *in vitro* experiments [86,130] (S2 Fig). Production of IFN by macrophages (*p*_*F*,*M*Φ_; Section S4.2.6) was obtained by fitting to data measuring IFN-α production [62] (S3 Fig). The parameters regulating the rate of the resident macrophage pool replenishment (*λ*_*M*Φ_ and *ϵ*_*V*,*M*Φ_; Section S4.2.7) were estimated from our *in vivo* observations of resident macrophages during influenza virus infection (S3 Fig). GM-CSF production by monocytes (*p*_*G*,*M*_; S3 Fig, Section S4.2.8), IFN production by infected cells (*p*_*F*,*I*_; Section S4.2.9), and IL-6 production by infected cells and macrophages (*p*_*L*,*I*_ and *p*_*L*,*M*Φ_; Section S4.2.10-S4.2.11) were all obtained from fitting reduced versions of **Eqs. S1-S22** to *in vitro* experiments [56,57,131,132] (S4 Fig).

Lastly, any remaining parameters values were obtained by ensuring that homeostasis was maintained in absence of infection (S5 Fig; Section S5). Parameters calculated from homeostasis include the half-effect monocyte concentration for G-CSF production (*η*_*C*,*M*_), the production rate of IL-6 and GM-CSF by inflammatory macrophages (*p*_*L*,*M*Φ_ and *p*_*G*,*M*Φ_), the production rate of monocytes by GM-CSF (*p*_*M*,G_), and the half-effect inflammatory macrophage concentration for IFN production (*η*_*F*,*M*Φ_). For some parameters it was not possible to obtain an estimation from the literature, and for these we either set their value equal to an already estimated parameter ($\epsilon_{L,N},p_{C,M},p_{F,M\Phi_{I}},\eta_{G,M\Phi },$), or qualitatively estimated it (*ϵ*_*I*,*M*_, *ρ*, see S1 Table).

For the ‘IFN model’ (Eqs 10–16), parameters related to virus (*p*, *d*_*V*_, *β* and *d*_*I*_), epithelial cell proliferation (*λ*_*S*_ and *S*_*max*_), and IFN ($p_{F,I},\;\eta_{F,I},\;k_{lin_{F}},\;k_{B_{F}},\;A_{F},\;k_{U_{F}}$ and *ϵ*_*F*,*I*_) were fixed to those in S1 Table.

### Numerical simulations

All ODE models were solved using *ode45* in MATLAB, and delay differentiation equations (i.e. **Eqs. S1-S22**) were solved using *ddesd* in MATLAB.

### Sensitivity analysis

We performed a local sensitivity analysis for the full model (**Eqs. S1-S22**) by individually varying each parameter by ±20% from its estimated value and quantifying the effect on the model’s output. This change was recorded and used to evaluate different metrics representing the inflammatory response to SARS-CoV-2, namely maximum viral load, maximum number of dead cells, minimum uninfected tissue, maximum number of inflammatory macrophages, maximum number of CD8^+^ T cells, maximum unbound IL-6, maximum unbound IFN, the total exposure (AUC) to type I IFN, number of days the percent of damaged tissue was >80%, and time of unbound type I IFN peak. We quantified the fraction of undamaged tissue by (*S* + *R*)/*S*_*max*_. For each parameter simulation, we recorded the value of the different metrics listed (i.e. maximum viral load etc.). For each metric, we then determined the maximum increase and decrease for that metric, and assigned the grid point for that parameter value a colour based on a linear grid of possible values for that metric.

### Virtual patient generation

We next created a virtual cohort of patients to extend the sensitivity analysis in Fig 5 and further interrogate on the causes driving responses for the most sensitive parameters (particularly certain IFN, IL-6, and immune cell related parameters). To generate a cohort of 200 virtual patients, we followed frequently-used quantitative systems pharmacology techniques similar to those of Allen et al. [32] and our previous studies [94,96] wherein individual virtual patients were created by sampling a parameter set ***p*** from parameter distributions then simulating the model to verify that each individual’s trajectory was realistic. A subset of parameters ($p_{M\Phi_{I},L}$, *p*_*L*,*M*Φ_, *p*_*F*,*I*_, *p*_*M*,*I*_, *η*_*F*,*M*Φ_, *ϵ*_*F*,*I*_, and *p*_*F*,*M*_) was designated as patient-specific after considering the results of the sensitivity analysis (Fig 5 and S9 Fig) and standard deviations inferred from clinical observations (Supplementary Information Section 6.1). Patient-specific parameters were selected using the *mvnrand* function in Matlab, which samples from a multivariate normal distribution. To avoid the inclusion of unrealistic dynamics, patient parameter sets were then optimized using simulated annealing using the *simulannealbnd* function in Matlab to ensure predictions fell within physiological ranges for viral load [47], IL-6 [6,53], IFN-α [51], and G-CSF [30] (Fig 8). Posterior distributions from this generation procedure are provided in S10 Fig.

The upper *u*_*i*_ and lower *l*_*i*_ bounds for *V*, *L*_*U*_, *F*_*U*_ and *C*_*U*_ were based off these physiological ranges from Munster et al. [47] (viral loads), Herold et al. [53] (IL-6 concentrations), Trouillet-Assant et al. [51] (IFN dynamics), and Liu et al. [7] (G-CSF concentrations) as described in Supplementary Information Section S.6.1. Intervals for each patient-specific parameter set were restricted to four standard deviations from the mean or zero if the lower bound was negative. Given an initial patient specific parameter set ***p***, we used simulated annealing to minimize *J*(***p***), i.e.

$$\underset{p}{min}\;J\left(p\right)={\text{min}}_{p}\left[\sum_{i}\text{max}\left({\left(M_{i}\left(p\right)-\frac{l_{i}+u_{i}}{2}\right)}^{2}-{\left(u_{i}-\frac{l_{i}+u_{i}}{2}\right)}^{2},0\right)\right],$$ (17)

where *M*_*i*_(***p***) is the model output *i* evaluated at parameter set ***p*** corresponding to the upper and lower bound *l*_*i*_ and *u*_*i*_ (Fig 8).

To quantify disease severity for each patient, we introduced an inflammation variable (Ψ) to account for the combined changes in IL-6 (*L*_*U*_), neutrophils (*N*), and damaged tissue (*S* + *R*), each normalized by the virtual cohort’s average. In this way, Ψ measures an individual’s relative change from the cohort’s baseline, and quantifies the contributions of IL-6, neutrophils, and tissue damage on comparable scales. For a given patient *j*, the inflammation marker is given by

$$\Psi^{j}=\frac{{\text{max}}_{t}\left(L_{U}^{j}(t\right))}{\frac{1}{n}\sum_{j=1}^{n}\left({\text{max}}_{t}\left(L_{U}^{j}\left(t\right)\right)\right)}+\frac{{\text{max}}_{t}\left(N^{j}(t\right))}{\frac{1}{n}\sum_{j=1}^{n}\left({\text{max}}_{t}\left(N^{j}\left(t\right)\right)\right)}+\frac{S_{max}-{\text{min}}_{t}\left(S^{j}\left(t\right)+R^{j}\left(t\right)\right)}{\frac{1}{n}\sum_{j=1}^{n}\left(S_{max}-{\text{min}}_{t}\left(S^{j}\left(t\right)+R^{j}\left(t\right)\right)\right)},$$ (18)

where *n* is the total number of patients in the cohort, and $L_{U}^{j},\;N^{j},S^{j}$, and *R*^*j*^ are the unbound IL-6, neutrophils, and susceptible and resistant epithelial cell count, respectively. The threshold value for severe diseases (Ψ = 3) was determined given the distinct “jump” in the delay in peak concentrations (Fig 9).

### Statistical analyses

The Pearson correlation coefficient (R) was used to measure the degree of interaction between two variables, with a significance level of *α* < 0.05 indicating rejection of the hypothesis that there is no relationship between the observed variables. In addition, we used two-sample two-sided t-tests (number of patients < 40) and z-tests (number of patients ≥ 40) at the α < 0.05 significance level to test the hypothesis that there were no differences between sample means.

## Supporting information

S1 Text Supplementary information file. (PDF)

S1 Fig Effects of neutrophils on lung epithelial cells, GM-CSF on monocyte production and differentiation, the relationships between monocytes and CD4+ T cells with IL-6, and the influence of IFN on T cell expansion. **A)** Using the measurements by Knaapen et al. [123], the inhibitory effect curve E (**Eq. S25**) was fit to the cell viability of RLE cells under various concentrations of H_2_O_2_. **B)** The stimulatory effect curve E (**Eq. S24**) was fit to the dose response measurements of blood monoculture cells (3 × 10^3^ cells/dish) with various concentrations of murine recombinant GM-CSF (IU/ml) [124]. **C)** The stimulatory effect curve E (**Eq. S24**) was fit to measurements for the monocytic myeloid cell count as a function of GM-CSF [133] **D) Eq. S27** fit to time course data of IL-6 production from monocytes [125]. **E)** IL-6 stimulation of monocyte differentiation to macrophages modelled by the inhibitory effect curve E (**Eq. S24**) fit to the percentage of CD14+ cells (macrophages) as a function of the number of fibroblasts measured by Chomarat et al. [44]. **F)** Stimulatory effect curve E (**Eq. S24**) for IFN-γ stimulation on CD8+ T cells fit to measurements of the signalling in CD8+T cells for varying doses of IFN-γ [61]. Data (black) is plotted as either circles (D & E) or mean and standard deviation error bars (**A-C&F**); solid blue line: corresponding fit.
(TIF)

S2 Fig Dynamics of IL-6 on T cell expansion, epithelial cell growth, IL-6 internalization, neutrophil-induced damage, and macrophage phagocytosis. **A)** Effect curve (**Eq. S24**) for the IL-6 effect on T cell expansion fit to measurements CD4+ T cells from dilutions of IL-6 by Holsti and Raulet [126]. **B)** Exponential growth curve fit to the growth of A549 cells [127] **C)** The internalization rate of IL-6 (**Eq. S30**) fit to the fraction of internalized IL-6 [128]. D) Exponential decay fit to cell viability after H_2_O_2_ administration [129]. **E)** The macrophage clearance of apoptotic material (**Eqs. S31-S33**) was fit to the percentage of macrophages that had engulfed material over 25 hours [86]. **F)** The phagocytosis rate of extracellular virus by macrophages was obtained by fitting **Eqs. S34-S35** to the uptake of virus by macrophages measured by Rigden et al. [130]. Data (black) is plotted as either circles (**A & F**) or mean and standard deviation error bars (**B-E**); solid blue line: corresponding fit.
(TIF)

S3 Fig Monocyte expansion and type I IFN production by monocytes, alveolar macrophage replenishment after viral infection, and GM-CSF production by monocytes. **A) Eq. S37** fit to time course of proliferation of monocytes in culture [62]. **B)** Fit of **Eqs. S38-S39** to the production of IFN-α by monocytes after 24 hours with RSV as a function of the number of days of pre-culturing (1, 2, 4 or 7) [63]. **C)** Correlation between infectious virus titre and RT-PCR copy number for influenza A and B measured by Laurie et al. [134] The relative TCID_50_ compared to the RNA copies is plotted for each virus strain and the mean as a black dashed line. **D-E)** Fit of **Eqs. S40-S42** to viral loads [135] and alveolar macrophages from experimental influenza infections. **F)** The production of GM-CSF from stimulated monocytes was recorded by Lee et al. [131] Using a simplified version of the full model (**Eqs. S43-S46**), we obtained the production rates for monocytes and GM-CSF. Data (black) is plotted as either circles/stars (**B&F**) or mean and standard deviation error bars (**A, D-E**); solid blue line: corresponding fit.
(TIF)

S4 Fig Production of IFN and IL-6 by infected cells and macrophages. **A)** Concentration of IFN-β released by alveolar epithelial cells in response to stimulation with influenza virus recorded at 8, 16 and 24 hours [57]. **B-C)** IL-6 production by infected cells in response to **A)** H5NA and **B)** H7N9, measured by Ye et al. [132] Data (black) is plotted as mean and standard deviation error bars with the corresponding fit (**Eqs. S51-S54**) in solid blue. **D)** IL-6 production by macrophages (**Eq. S56**) in response to stimulation with LPS of varying dosage sizes. Shibata et al. [56] measured the production of IL-6 for different dosages of LPS and fitting the production rate to this data to obtain p_L,MΦ_, η_L,MΦ_.
(TIF)

S5 Fig Homeostatic disease-free system regulation. **A)** To confirm that parameters in the model represented realistic immunocompetent individuals in the disease-free scenario, **Eqs. S1-S22** were simulated where V_0_ = 0 and parameters were given by the homeostasis **Eqs. S57-S70**. The initial concentration of G-CSF was perturbed and compared to simulations of the model at homeostasis. Simulations at homeostasis are represented by solid lines (purple) and perturbed simulations as dashed lines (pink). **B)** The maximum residual between variables and their initial conditions at day 50 was measured to confirm that the system was stable for perturbations in all immune cells and cytokines.
(TIF)

S6 Fig Viral dynamics model fit to macaque viral data from Munster et al. [47] A reduced version of the full model (all cytokine and immune cells set to 0, Eqs 6–9) was fit to data from macaques [47] to estimate preliminary viral kinetic parameters. **A)** Virus (*V*) infects susceptible cells (*S*) making infected epithelial cells (*I*) which then die to produce dead cells (*D*) and new virus. **B)** Comparison of predicted viral dynamics compared to observations from 6 animals, with susceptible cell kinetics (left) with predictions of infected and dead cells over time (right). We estimated *β*, *p*, *d*_*I*_, *V*_0_ and *d*_*V*_ from the reduced model in **A)** fit to data from Munster et al. [47] measuring the viral load in macaques after challenge with SARS-CoV-2.
(TIF)

S7 Fig Model validation against human cytokine and immune cell measurements during SARS-CoV-2 infection. **A)** IFN dynamics of the reduced model (Fig 3 **Main Text**) overlaid with patient IFN-α2 plasma concentrations from Trouillet-Assant et al. [100] The solid line (purple) represents the unbound IFN dynamics from the reduced model (**Eqs. 27–33**). Individual patient IFN-α2 measurements are plotted as grey circles for IFN-positive patients (n = 21), patients with no IFN measurements (IFN-negative; n = 5) have not been plotted. Healthy volunteer IFN-α2 concentrations are indicated by a grey area. **B-F)** Mild (solid line) and severe (dashed line) dynamics (**Eqs. 27–33** corresponding to simulations in Fig 4 **Main Text** and S8 Fig compared to corresponding measurements in humans. B-C) Plasma IFN-α and IL-6 in COVID-19 critically ill patients (n = 26) obtained by Trouillet-Assant et al. [100] overlaid with mild and severe unbound IFN (F_U_(t)) and mild and severe unbound IL-6 (L_U_(t)) IFN-negative patients (yellow stars) had no IFN-α measurements and IFN-positive patients (grey points) had non-zero IFN-α measurements. Healthy volunteer concentrations are indicated by a grey area. **D)** IL-6 levels in patients requiring (“Yes”) and not requiring mechanical (“No”) ventilation obtained by Herold et al. [53] overlaid with mild and severe unbound IL-6 dynamics. **E)** IL-6 concentration in Moderate (“M”) and severe (“S”) COVID-19 patients obtained by Lucas et al. [6]. **F)** G-CSF plasma concentration obtained by Long et al. [30] in symptomatic “S” and asymptomatic “AS” COVID-19 patients overlaid with corresponding mild and severe model dynamics. **G-I)** Neutrophils, monocytes and CD8^+^ T cells in moderate and severe COVID-19 patients normalized by health care worker (HCW) baseline measurements obtained by Lucas et al. [6]. Violin plots are given for the measurements plotted in D-I.
(TIF)

S8 Fig Predicting mild and severe COVID-19 dynamics (all model variables). Extension of results of mild and severe disease dynamics in Fig 4 **Main Text**. Mild disease (solid lines) dynamics obtained by using baseline parameter estimates (S1 Table) while severe disease dynamics (dashed lines) were obtained by decreasing the production rate of type I IFN, p_F,I_, and increasing the production of monocytes, p_M,I_, and their differentiation to macrophages, η_F,MΦ_. **A)** Lung cells concentrations (susceptible cells S(t), resistant cells R(t), infected cells I(t), dead cells D(t) and virus V(t)). Solid black line with error bars indicates macaque data (see Fig 2 **Main Text**). **B)** Immune cell concentrations (resident macrophages M_ΦR_(t), inflammatory macrophages M_ΦI_(t), monocytes M(t), neutrophils N(t) and T cells T(t)). **C)** Bound and unbound cytokine concentrations (IL-6 unbound L_U_(t) and bound L_B_(t), GM-CSF unbound G_U_(t) and bound G_B_(t), G-CSF unbound C_U_(t) and bound C_B_(t), type I IFN unbound F_U_(t) and bound F_B_(t)).
(TIF)

S9 Fig Full analysis of parameters driving COVID-19 severity. A local sensitivity analysis was performed by varying each parameter ±20% from its originally estimated value and simulating the model. Predictions were then compared to baseline considering: Maximum viral load (max(V)), maximum concentration of dead cells (max(D)), minimum uninfected live cells (min(S+R)), maximum concentration of inflammatory macrophages (max(M_ΦI_)), maximum number of CD8^+^ T cells (max(T)), maximum concentration of IL-6 (max(L_U_)), maximum concentration of type I IFN (max(F_U_)), the total exposure to type I IFN (F_U_ exposure), the number of days damaged tissue was >80% (time (S + R)/S_max_)<0.2), and the day type I IFN reached its maximum (day max(F_U_)). The heatmaps show the fold change of each metric, where blue signifies the minimum value observed and red signifies the maximum value observed, or by the number of days, where light to dark pink signifying increasing number of days from zero. The most sensitive parameters are shown in Fig 5 in the Main Text.
(TIF)

S10 Fig Effects of neutrophil, monocyte, and macrophage knockout on mild disease courses compared with severe disease dynamics. We performed in silico knockout experiments in the mild disease scenario (Fig 4; solid black line) by considering complete monocyte knockout (i.e. no monocyte recruitment and M(0) = 0; dark pink dash-dot line), complete macrophage knockout (i.e. not inflammatory macrophage creation via antigen stimulation or monocyte differentiation; light pink dotted line) and complete neutrophil knockout (i.e. no neutrophil recruitment and N(0) = 0; pink dashed line). Blue solid lines correspond to mild diseases courses; black solid lines: severe disease. Dynamics of the in silico knockout are plotted for the **A)** viral load, **B)** uninfected cells, **C)** inflammatory macrophages, **D)** neutrophils, **E)** CD8+ T cells relative to uninfected cells and **F)** unbound IL-6. This figure is an extension of Fig 7 in the Main Text.
(TIF)

S11 Fig Parameter distributions of virtual cohort parameters. Virtual patients were generated by sampling from normal distributions for a subset of model parameters and optimizing parameters using simulated annealing to confirm realistic disease trajectories (see Fig 4). Resulting distributions for each parameter (purple histograms) are shown compared to estimated values for an average patient (dotted black vertical line). The average of the virtual cohort is also plotted (dashed purple line).
(TIF)

S12 Fig Cohort dynamics compared to observed clinical physiological ranges. Virtual patients were generated so that viral load, IFN, G-CSF and IL-6 concentration were within physiological ranges obtained in the literature. The physiological ranges (Fig 9) of **A)** human SARS-CoV-2 viral loads [37], **B)** IL-6 concentrations from patients [53] plotted against the virtual cohort dynamics (grey lines).
(TIF)

S1 Table Parameter values used in the Main Text. Parameters have been grouped into: (a-e) cell related, (f-k) cytokine related parameters, and (l) initial conditions. Relevant references are given for parameters directly estimated from the literature (“Direct estimate”). Parameters obtained through fitting to data in the literature have the appropriate figure noted and data referenced (“Fit Fig, data”). Remaining parameters were estimated from homeostasis calculation (“Homeostasis”) or qualitatively estimated (“Estimated”). Parameters whose value was taken from another parameters estimation has that parameter noted. Viral load is reported as log(virion copies/ml) and cells have been noted in 10^9^ cells/ml. Time t is in days. The final sub-table (m) is a list of the variables in the model.
(PDF)
