# Supplementary material for: COVID-19 virtual patient cohort suggests immune mechanisms driving disease outcomes
Source: PLoS Pathog. 2021 Jul 14;17(7):e1009753. doi: 10.1371/journal.ppat.1009753 (PMC8312984; doi:10.1371/journal.ppat.1009753)
Supplement: S1 Text — (PDF) [file ppat.1009753.s001.pdf]

Supplementary Information for: COVID-19 virtual patient cohort suggests immune mechanisms driving disease outcomes

Adrianne L. Jenner, Rosemary A. Aogo, Sofia Alfonso, Vivienne Crowe, Xiaoyan Deng, Amanda P. Smith, Penelope A. Morel, Courtney L. Davis, Amber M. Smith, Morgan Craig

Correspondence to: morgan.craig@umontreal.ca

In the Supplementary Information, we detail the full model equations (Section S1), the parameters and their values (Section S2), and how parameters were estimated (Section S3-S5). Parameters in the model were either obtained from the literature (Section S3), through fitting to dose response data (Section S4.1) or through fitting to time-series measurements (Section S4.2). Remaining parameters were estimated through calculating homeostasis (Section S5). Validation against macaque SARS-CoV-2 infection measurements, and further information for the sensitivity analysis and virtual cohort simulations is also provided (Section S6). A summary of all variables and parameters in the full model (**Eqs. S1-S22**) can be found in **S1 Table**.

S1. Mathematical model of the immune response to SARS-CoV-2

To model the immune response to SARS-CoV-2 infection, we constructed a system of ordinary and delay differential equations (**Fig 1, Main Text**). The model considers a population of susceptible lung cells (S) that become infected (I) by SARS-CoV-2 virus (V). Infected cells become damaged or dead (D) through the virus induced lysis or immune involvement. Upon infection, cells begin secreting type I IFN (F_U, F_B)which reduces viral infection and results in cells resistant to virus infection (R).

Alveolar macrophages ($M_{\Phi R}$) are activated by infected or dead cells and become inflammatory macrophages ($M_{\Phi I}$) and begin secreting IL-6 (L_U, L_B) and GM-CSF (G_U, G_B). Monocytes (M) are recruited by the presence of infected cells and stimulated by GM-CSF to differentiate into inflammatory macrophages. Neutrophils (N) are recruited by G-CSF (C_U, C_B) and contribute to bystander death of epithelial cells through the release of reactive oxygen species (ROS). CD8$^+$ T cells (T) are recruited after a delay from initial infection and induce apoptosis in infected cells. Bound and unbound concentrations are modelled explicitly. Equations for these interactions are given below:

$$\frac{dV}{dt} = pI - \delta_{V,M\Phi} M_{\Phi I} V - \delta_{V,N} NV - d_V V, \qquad S1$$

$$\frac{dS}{dt} = \lambda_S \left(1 - \frac{S + I + R + D}{S_{max}}\right) S - \beta SV - \frac{\rho \delta_N S N^{h_N}}{N^{h_N} + {IC_{50,N}}^{h_N}}, \qquad S2$$

$$\frac{dI}{dt} = \frac{\beta \epsilon_{F,I}}{F_B + \epsilon_{F,I}} S(t-\tau_I)V(t-\tau_I) - d_I I - \frac{\delta_N I N^{h_N}}{N^{h_N} + {IC_{50,N}}^{h_N}} - \delta_{I,M\Phi} M_{\Phi I} I - \delta_{I,T} TI, \qquad S3$$

$$\frac{dR}{dt} = \lambda_S \left(1 - \frac{S + I + R + D}{S_{max}}\right) R + \frac{\beta F_B}{F_B + \epsilon_{F,I}} S(t-\tau_I)V(t-\tau_I) - \frac{\rho \delta_N R N^{h_N}}{N^{h_N} + {IC_{50,N}}^{h_N}}, \qquad S4$$

$$\frac{dD}{dt} = d_I I + \frac{\delta_N(\rho S + \rho R + I)N^{h_N}}{N^{h_N} + {IC_{50,N}}^{h_N}} + \delta_{I,M\Phi}(M_{\Phi I})I + \delta_{I,T} TI - d_D D + \left(\delta_{M\Phi,\mathrm{D}} - \delta_{D,M\Phi}\right)(M_{\Phi R} + M_{\Phi I})D, \qquad S5$$

$$\frac{dM_{\Phi R}}{dt} = -a_{I,M\Phi} M_{\Phi R}(I + D) - \delta_{M\Phi,\mathrm{D}} D M_{\Phi R} + \left(1 - \frac{M_{\Phi R}}{M_{\Phi max}}\right) \frac{\lambda_{M_\Phi} M_{\Phi I}}{V + \epsilon_{V,M\Phi}} - d_{M\Phi_R} M_{\Phi R}, \qquad S6$$

$$\frac{dM_{\Phi I}}{dt} = a_{I,M\Phi} M_{\Phi R}(I+D) + \frac{p_{M_{\Phi I},G} {G_B}^{h_{M,M\Phi}} M}{{G_B}^{h_{M,M\Phi}} + {\epsilon_{G,M_{\Phi I}}}^{h_{M,M\Phi}}} + \frac{p_{M_{\Phi I},L} L_B M}{L_B + \epsilon_{L,M_\Phi}} - d_{M_{\Phi I}} M_{\Phi I} - \delta_{M\Phi,\mathrm{D}} D M_{\Phi I} - \left(1 - \frac{M_{\Phi R}}{M_{\Phi max}}\right) \frac{\lambda_{M_\Phi} M_{\Phi I}}{V + \epsilon_{V,M\Phi}}, \quad S7$$

$$\frac{dM}{dt} = \left(M^*_{prod} + \left(\psi^{max}_M - M^*_{prod}\right) \frac{{G_B}^{h_M}}{{G_B}^{h_M} + {\epsilon_{G,M}}^{h_M}}\right) M_R + \frac{p_{M,I} I M}{I + \epsilon_{I,M}} - \frac{p_{M_{\Phi I},G} {G_B}^{h_{M,M\Phi}} M}{{G_B}^{h_{M,M\Phi}} + {\epsilon_{G,M_{\Phi I}}}^{h_{M,M\Phi}}} - \frac{p_{M_{\Phi I},L} L_B M}{L_B + \epsilon_{L,M_\Phi}} - d_M M, \quad S8$$

$$\frac{dN}{dt} = \left(N^*_{prod} + \left(\psi^{max}_N - N^*_{prod}\right) \frac{C_{BF} - C^*_{BF}}{C_{BF} - C^*_{BF} + \epsilon_{C,N}}\right) N_R + \frac{p_{N,L} L_B}{L_B + \epsilon_{L,N}} - d_N N, \quad S9$$

$$\frac{dT}{dt} = \frac{p_{T,I} I(t - \tau_T) \epsilon_{L,T}}{L_B + \epsilon_{L,T}} + \frac{p_{T,F} F_B T}{F_B + \epsilon_{F,T}} - d_T T, \quad S10$$

$$\frac{dL_U}{dt} = \frac{p_{L,I} I}{I + \eta_{L,I}} + \frac{p_{L,M_{\Phi I}} M_{\Phi I}}{M_{\Phi I} + \eta_{L,M_{\Phi \mathrm{I}}}} + \frac{p_{L,M} M}{M + \eta_{L,M}} - k_{lin_L} L_U - k_{B_L}\big((M + N + T)A_L - L_B\big) L_U + k_{U_L} L_B, \quad S11$$

$$\frac{dL_B}{dt} = -k_{int_L} L_B + k_{B_L}\big((M + N + T)A_L - L_B\big) L_U - k_{U_L} L_B, \quad S12$$

$$\frac{dG_U}{dt} = \frac{p_{G,M_{\Phi I}} M_{\Phi I}}{M_{\Phi I} + \eta_{G,M\Phi}} + \frac{p_{G,M} M}{M + \eta_{G,M}} - k_{lin_G} G_U - k_{B_G}(M A_G - G_B) G_U + k_{U_G} G_B, \quad S13$$

$$\frac{dG_B}{dt} = -k_{int_G} G_B + k_{B_G}(M A_G - G_B) G_U - k_{U_G} G_B, \quad S14$$

$$\frac{dC_U}{dt} = \frac{p_{C,M} M}{M + \eta_{C,M}} - k_{lin_C} C_U - k_{B_C}(N A_C - C_B)(C_U)^{POW} + k_{U_C} C_B, \quad S15$$

$$\frac{dC_B}{dt} = -k_{int_C} C_B + k_{B_C}(N A_C - C_B)(C_U)^{POW} - k_{U_C} C_B, \quad S16$$

$$\frac{dF_U}{dt} = \frac{p_{F,I} I}{I + \eta_{F,I}} + \frac{p_{F,M_{\Phi I}} M_{\Phi I}}{M_{\Phi I} + \eta_{F,M_{\Phi I}}} + \frac{p_{F,M} M}{M + \eta_{F,M}} - k_{lin_F} F_U - k_{B_F}\big((T + I)A_F - F_B\big) F_U + k_{U_F} F_B, \quad S17$$

$$\frac{dF_B}{dt} = -k_{int_F} F_B + k_{B_F}\big((T + I)A_F - F_B\big) F_U - k_{U_F} F_B, \quad \mathrm{S18}$$

where

$$A_L = \frac{MM_L}{6.02214 \times 10^{23}} \left(K_{L,N} + K_{L,T} + K_{L,M}\right) \cdot \left(\frac{10^{-3}}{5000}\right), \quad S19$$

$$A_G = \frac{MM_G}{6.02214 \times 10^{23}} K_{G,M} \cdot \left(\frac{10^{-3}}{5000}\right), \quad S20$$

$$A_C = \hat{p} \frac{MM_C}{6.02214 \times 10^{23}} K_{C,N} \cdot \left(\frac{10^{1}}{5000}\right), \quad S21$$

$$A_F = \frac{MM_F}{6.02214 \times 10^{23}} \left(K_{F,T} + K_{F,I}\right) \cdot \left(\frac{10^{-3}}{5000}\right). \quad S22$$

S2. Model parameter values

A summary of each parameter value in **Eqs. S1-S22** is provided in **S1 Table** with references. In **S1 Table**, the *Estimation and citation* column indicates whether the value was estimated directly from a quantity in the literature or fit using data in the literature (with reference to the relevant figure). A summary of the variables in the model is given at the end of **S1 Table**.

S3. Parameters taken from literature

S3.1. Initial cytokine concentrations

The basal concentrations of unbound cytokines were taken from values in the literature. The plasma concentration of colony stimulating factor GM-CSF in healthy adults was measured using immunoassay by Lee et al.[65] to be 2.43 ± 0.42 pg/ml (i.e., $G_{U,0} = 2.43$ pg/ml). We fixed the initial unbound G-CSF cytokine concentration to $C_{U,0} = 0.025$ ng/ml[14]. The concentration of unbound IFN type 1 was set to be $F_{U,0} = 0.015$ pg/ml based on the average value of IFN-α in humans[67,68] determined by Simoa IFN-α assay. The median plasma IL-6 concentration was estimated to be $L_{U,0} = 1.1$ pg/ml in blood samples from healthy adults[69] by ELISA.

S3.2. Initial cell populations

The average total number of type I and type II alveolar epithelial cells and endothelial cells in the lung was estimated by Crapo et al.[3] to be 136×10^9 cells from eight people (6 males, 2 females) aged 19-40 using morphometric analysis. At functional residual capacity, pulmonary total tissue volume was reported by Armstrong et al.[60] to be 843 ± 110 ml, measured by finding the difference between the radiographic displacement volume of the thorax and the lung gas volume. Together, this gave an initial target cell concentration of $S_0 = 0.16 \times 10^9$ cells/ml. Crapo et al.[3] found the average number of alveolar macrophages to be $23 \pm 7 \times 10^6$ cells (using morphometric analysis), thus $M_{\Phi R,0} = 2.73 \times 10^5$ cells/ml. Monocytes account for 1% to 10% of circulating white blood cells, which equates to 200 to 600 monocytes per microliter of blood[61] (with a blood volume of 5 litres[70]). Therefore, we assumed that at homeostasis $M_0 = 4 \times 10^5$ cells/ml. For the total number of neutrophils in the blood, we used the previous estimate of Craig et al.[14] $N_0 = 5.26 \times 10^6$ cells/ml that was calculated from whole blood and marginated neutrophils. Lastly, the number of CD8⁺ T cells in the lung tissue was, on average, 20% of the number of CD8⁺ T cells in the blood[63] estimated from enzymatic and mechanical digestion. Using flow cytometry, Uppal et al.[62] determined there were 552 cells/μl, on average, in the blood. To account for the number of naïve CD8⁺ T

cells infiltrating the lungs[71], the initial number of CD8$^+$ T cells was estimated from the proportion of T cells in the tissue, i.e. $T_0 = 1.1 \times 10^5$ cells/ml.

S3.3. Cytokine molecular weights and receptors per cell

Cytokine binding and unbinding kinetics were modelled using the molecular weight of each cytokine and the number of corresponding receptors on the binding cell. The molecular weight for IL-6 is $MM_L = 21{,}000$ g/mol[57]; for GM-CSF is $MM_G = 14{,}000$ g/mol[58]; for G-CSF is $MM_C = 19{,}600$ g/mol[14]; and for IFN-β is $MM_F = 19{,}000$ g/mol[59]. The number of high-affinity receptors for GM-CSF on the surface of blood monocytes[53] is $K_{G,M} = 1{,}058$ sites/cell (characterized by an *in vitro* binding assay). Mature human neutrophils express ~200-1,000 G-CSF receptors per cell[54], thus we fixed $K_{C,N} = 600$ sites/cell. Most cells have 1,000-2,000 type I IFN receptors (IFNAR) receptors[55]. Assuming CD8$^+$ T cells are at the lower end of this interval gives $K_{F,T} = 1{,}000$ sites/cell. The number of IFNAR sites on HEC1B human (uterus/endometrium epithelial) cells is on average $K_{F,I} = 1{,}300$ sites/cell[56] (characterized by an *in vitro* binding assay). The number of IL-6 receptors on CD8$^+$ T cells is $K_{L,T} = 300$ sites/cell based on measurements from Taga et al.[51] from an *in vitro* binding assay for IL-6. We fixed the number of IL-6 receptors on neutrophils and monocytes to $K_{L,N} = 720$ sites/cell and $K_{L,M} = 509$ sites/cell respectively, based on the range in IL-6 receptors expressed on myeloma hematopoietic cells[50] and IL-6 receptors on mouse myelomonocytic leukemic M1 cells[52].

S3.4. Binding/unbinding rates of cytokines

Based on *in vitro* ELISA measurements by Tenhumberg et al.[49], the binding and unbinding rate of IL-6 was set to be $k_{B_L} = 0.0018$ pg/ml/day and $k_{U_L} = 22.29$/day, respectively. The unbinding kinetics for G-CSF were previously estimated by Craig et al.[14] to be $k_{U_C} = 184.87$/day. Lastly, the unbinding and binding rates for GM-CSF were taken from previous modelling work[47] and set to $k_{U_G} = 522.72$/day and $k_{B_G} = 0.0021$ per pg/ml/day respecitvely. Mager and Jusko[48] estimated the binding rate of IFN-β using a PKPD model to be $k_{B_F} = 0.0107$ per pg/ml/day. Lastly, the binding rate for GM-CSF was fixed as $k_{B_G} = 0.0021$ per pg/ml/day[47] extracted from a curve fitting analysis of the approach to steady state of surface bound and internalized G-CSF molecules.

S3.5. Clearance and internalization rate of cytokines

Most of the clearance and internalization rates of cytokines in **Eqs. S1-S22** were obtained by assuming exponential clearance and using the half-life formula:

$$k_{lin} = \frac{\ln(2)}{t_{1/2}}, \qquad S23$$

where $t_{1/2}$ is the cytokine half-life and k_{lin} the clearance rate. IL-6 has a short half-life in circulation of approximately 1 hour[43] giving a clearance rate of $k_{lin_L} = 16.6$/day. The half-life of GM-CSF in circulation ranges between 50-85 minutes[44]. We took the upper value giving $k_{lin_G} = 11.74$/day. The linear clearance rate of G-CSF was previously estimated by Craig et al.[14] as $k_{lin_C} = 0.16$/day. Terminal elimination half-lives for IFN-β range from 1-2 hours[45]. Using the lower bound, we fixed the clearance rate as $k_{lin_F} = 16.6$/day, giving a half-life of roughly 55 minutes. The internalization rates of GM-CSF and G-CSF were taken from previous pharmacokinetic modelling[14,47] and fixed to $k_{int_G} = 73.4$/day and $k_{int_C} = 462$/day. Similarly, the internalization rate of IFN was fixed as $k_{int_F} = 16.97$/day based on previous modelling of the receptor-mediated dynamics of IFN-β[45,72]. The internalization rate of IL-6, k_{lin_L}, was estimated by data fitting (Section S4.2.2).

S3.6. Neutrophil and monocyte reservoir dynamics

Craig et al.[14] previously developed a physiological model of the production dynamics of neutrophils through G-CSF regulation that accounts for the concentration of freely circulating cytokine and cytokine bound to mature neutrophils. The parameters in our model that relate to the number of neutrophils in the bone marrow reservoir, release rate, and the dynamics of G-CSF on neutrophils (**Eq. 5** & **Eq. S9**) were taken from their work, i.e. $N_R = 3.16 \times 10^7$ cells/ml, $C_{BF}^* = 1.58 \times 10^{-5}$ (unitless), $\epsilon_{C,N} = 1.8924 \times 10^{-4}$ (unitless), and $\psi_N^{max} = 4.13/\text{day}$. Similarly, the bone marrow monocyte reservoir dynamics were estimated based on previous modelling work by Cassidy et al.[13] to be $M_R = 2.27 \times 10^6$ cells/ml and $\psi_M^{max} = 11.55/\text{day}$.

S3.7. Monocyte, macrophage differentiation and activation rates

Previous mathematical modelling studies were used to estimate the monocyte and macrophage differentiation and activation rates. We assumed that the recruitment rate of monocytes would be equal to the recruitment rate of new macrophages by infected cells[10], giving $p_{M,I} = 0.22$/day. We approximated the activation rate of resident macrophages to inflammatory macrophages from the rate of dendritic cell activation[8,9], giving $a_{I,M\Phi} = 1.1 \times 10^3$ per 10^9 cells/ml/day. Lastly, the GM-CSF and IL-6 stimulated differentiation rate of monocytes to macrophages was $p_{M_{\Phi I},G} = 1.7$/day based on the estimates that it can take 12-14 hours for monocytes to migrate from the bone marrow to the site of inflammation and subsequently differentiate into progenitor cells[7].

S3.8. CD8⁺ T cell recruitment and expansion rate

The maximal time for $CD8^+$ T cell division is between 4-6 hours[11], giving a production rate of $p_{T,F} =$ 4/day. The dynamics of $CD8^+$ T cells in response to infected cells were modelled similarly to previous work by de Pillis et al.[73] and Baral et al.[12], and we set the activation rate and half-effect parameter of infected cells from these studies, i.e. $p_{T,I} = 9 \times 10^{-3}$/day and $\epsilon_{T,I} = 10^3$ cells/ml. Lastly, $\tau_T = 4.5$ days based on the delay in infected cell recruitment of $CD8^+$ T cells[6].

S3.9. Cell death rates

Neutrophils are known to have a short half-life in circulation[33] of $d_N = 1.28$/day. Kim et al.[34] estimated that primed $CD8^+$ T cells have a death rate of $d_T = 0.4$/day. Monocytes transiting from the bone marrow to the blood have a circulating half-life of 22 hours[32], using the half-life formula (**Eq. S23**) this gives $d_M =$ 0.756/day. The time from initiation of cell apoptosis to completion can occur as quickly as 2-3 hours[29] giving a dead cell decay rate of $d_D = 8$/day. At homeostasis, mature macrophages are a quiescent population with a half-life between 4-6 weeks[30], or sometimes greater than 80 days[74]. Thus, we assumed that the death of resident alveolar macrophages is negligible ($d_{M\Phi R} = 0$/day) given the time frame of acute SARS-CoV-2 infections considered in this study (3 weeks). Inflammatory macrophages were assumed to have a death rate of $d_{M_{\Phi I}} = 0.3$/day, which was estimated through inflammatory macrophages in response to oncolytic virotherapy[31]. Macrophages also undergo apoptosis from phagocytosing too much material (exhaustion). We assumed it takes ~20 dead cells to be phagocytosed to induce macrophage death[26,27] and set $\delta_{M\Phi,D} = 6.06$ per 10^9 cells/day. The rate of $CD8^+$ T cell-induced apoptosis of infected cells was previously estimated by Lee et al.[25] giving $\delta_{I,T} = 238$ per 10^9 cells/day. The phagocytosis rate of infected cells by macrophages $\delta_{I,M\Phi} = 121.195$ per 10^9 cells/day was estimated from neutrophil phagocytosis[24].

S4. Parameters estimated by data fitting

S4.1. Pharmacodynamics of stimulatory and inhibitory effects for cells and cytokines

We used standard pharmacodynamic relationships to model the various immunological effects of cells and cytokines. Here, the half-maximal response (generally expressed as an EC_{50} or IC_{50}) of the cytokine or cell population is the concentration at which half of the maximal (stimulatory of inhibitory) effect is achieved[75]. Effect curves, E, (stimulatory or inhibitory respectively) are given by[76]

$$E = E_0 + E_{max}\frac{W^h}{W^h + EC_{50}^h}, \quad S24$$

$$E = E_0 + E_{max}\left(1 - \frac{W^h}{W^h + {IC_{50}}^h}\right), \quad S25$$

where E denotes the measured response (e.g., cell viability), W is the concentration of cytokine or cells under consideration, E_0 is the basal effect (the response when the dose of the compound is zero), E_{max} is the maximum effect (stimulatory of inhibitory) of the compound, $h > 0$ is the Hill coefficient that measures the sensitivity of the response to the dose range of the compound (i.e. the slope of the dose-response curve). **Eqs. S24-S25** are also known as Emax and Imax functions[75], see also **Eq. 1 Main Text**.

S4.1.1. Type I IFN inhibition of viral infection and replication

Sheahan et al.[15] measured the mean inhibition of MERS-CoV replication *in vitro* of IFN-β in Calu3 cells and found the EC_{50} to be 175 IU/ml. The specific activity of recombinant human IFN-β is approximately 2.8×10^8 IU/mg[15]. We converted the EC_{50} of 175 IU/ml to pg/ml to give the half-effect of IFN-β on viral production and infection capacity as $\epsilon_{F,I} = 625$ pg/ml[15]. Since this is a measurement of unbound IFN, we scaled this by the initial proportion of bound to unbound IFN ($F_{B,0}/F_{U,0}$) to get $\epsilon_{F,I} = 4.65 \times 10^{-4}$ pg/ml. As MERS-CoV and SARS-CoV-2 are both human coronaviruses (hCoVs) and have similar viral kinetics[77], we assumed that the mean inhibition of MERS-CoV replication can be used to approximate SARS-CoV-2 replication inhibition IFNβ.

S4.1.2. Neutrophil-induced damage of alveolar epithelial cells

To estimate the rate of neutrophil induced damage due to the release of reactive oxygen species (ROS) (**Eqs. S2-S4**), we used *in vitro* cell viability measurements of rat alveolar epithelial cells (RLE) after incubation for 2 hours with hydrogen peroxide (H_2O_2) at varying concentrations[21]. Fitting **Eq. S25** to this data, we obtained $IC_{50} = 197.63\mu M$ and $h = 3.02$ (**S1A Fig**). To convert this IC_{50} from a concentration of H_2O_2 to the concentration of neutrophils (as in **Eqs. S2-S4**), we used the approximate amount of H_2O_2 produced by a single neutrophil in response to stimulation by *N*-Formylmethionyl-leucyl-phenylalanine and phorbol myristate acetate[78]. Taking the average response from these stimuli and converting to units $\mu M/\text{cell}$, neutrophils produce $0.0042\mu M/\text{cell}$ of H_2O_2. As the maximum production of H_2O_2 by neutrophils is achieved relatively fast (15-30 minutes after stimulation[78]) we estimated the equivalent IC_{50} as a neutrophil concentration to be 4.71×10^4 cells (i.e. $197.62/0.0042$). As this represents a concentration of stimulated neutrophils we then increased this by the number of neutrophils at homeostasis[79] to give the half-effect concentration for neutrophil bystander damage of $IC_{50,N} = 4.71 \times 10^7$ cells/ml. This estimate was in line with estimates obtained from similar experiments conducted by Weiss et al[80] and Snyers et al.[50].

S4.1.3. Effect of GM-CSF on monocyte production and differentiation

GM-CSF can act in a paracrine fashion to recruit circulating monocytes, enhance their functions in host defense[81,82] and influence their differentiation into monocytic or granulocytic lineages[17]. Reducing **Eqs. S8** to consider only the effect of production and differentiation gives

$$\frac{dM}{dt} = \frac{p_{M,G}{G_B}^{h_M}}{{G_B}^{h_M} + {\epsilon_{G,M}}^{h_M}} - \frac{p_{M_{\Phi I},G}{G_B}^{h_{M,M\Phi}}M}{{G_B}^{h_{M,M\Phi}} + {\epsilon_{G,M_{\Phi I}}}^{h_{M,M\Phi}}}. \qquad S26$$

We modelled the effect of GM-CSF on monocyte production by estimating its potency from the dose-response of cultured blood monoculture cells with various concentrations of murine recombinant GM-CSF *in vitro* for 21 days[18]. Fitting **Eq. S24** gave $EC_{50} = 85.8$ IU/ml and $h = 1.67$ (**S1B Fig**). Using the specific activity of recombinant GM-CSF (i.e. 15×10^5 IU/µg [18]), this becomes $\epsilon_{G,M} = 57.2$ pg/ml. GM-CSF also promotes myeloid differentiation of cultured bone marrow cells into granulocytic and monocytic lineages towards terminal differentiation into monocytes, macrophages, and dendritic cells[17]. Sun et al.[17] investigated whether the dose of GM-CSF regulates the development of myeloid cells and measured the monocytic myeloid cell count as a function of GM-CSF concentrations. Fitting **Eq. S24** to this data, we obtained $EC_{50} = 2.66$ ng/ml and $h = 2.03$ (**S1C Fig**). Converting this to the units of GM-CSF in our model gives 2.7×10^3 pg/ml as the unbound GM-CSF half-effect concentration, which scaled by 10^{-5} (the order of initial bound GM-CSF to initial monocytes $G_{B,0}/M_0$) gives a bound GM-CSF half effect concentration of $\epsilon_{G,M\Phi} = 0.027$ pg/ml.

S4.1.4. IL-6 production by monocytes and effect on monocyte differentiation

Peripheral blood monocytes can be induced to secrete an array of cytokines, including IL-6, by stimuli such as lipopolysaccharide (LPS)[37]. The production of unbound IL-6 was modelled as a function of the monocyte concentration (**Eq. S11**):

$$\frac{dL_U}{dt} = \frac{p_{L,M}M}{M + \eta_{L,M}}. \qquad S27$$

Alderson et al.[37] measured the concentration of IL-6 (IU/ml) produced by monocytes stimulated with 10µl of LPS over 24 hours in 1ml of culture medium (**S1D Fig**). We fit **Eq. S27** to this data by assuming the number of monocytes was fixed to $M = 2 \times 10^5$ cells and that there was no monocyte proliferation over the course of the experiment. This gave an estimate of $p_{L,M} = 7.26 \times 10^4$ pg/ml/day (converted using 4.5 pg/ml as the concentration required for half-maximal stimulation of B9 proliferation by IL-6[83]) and $\eta_{L,M} = 9 \times 10^4$ cells. Scaling this half-effect by the initial concentration of monocytes gives $\eta_{L,M} = 4.98 \times 10^7$ cells/ml. We then scaled the production rate of IL-6 by 10^3 as the maximum IL-6 concentration achieved *in vivo* during SARS-CoV-2 infection was 10^3 less than that in the two *in vitro* experiments. We confirmed this production rate using the experiments of Morris et al.[84], which measure the production of IL-6 by blood mononuclear cells by co-culturing with airway smooth muscle cells (ASM) cells and LPS stimulation.

Production of macrophages by monocytes from stimulation with IL-6 was modelled as

$$\frac{dM}{dt} = -\frac{p_{M_{\Phi I},L}L_B M}{L_B + \epsilon_{L,M_\Phi}}, \qquad S28$$

$$\frac{dM_{\Phi I}}{dt} = \frac{p_{M_{\Phi I},L}L_B M}{L_B + \epsilon_{L,M_\Phi}}, \qquad S29$$

where $p_{M\Phi I,I}$ is the production rate and $\epsilon_{L,M\Phi}$ is the half effect from the bound IL-6 (**Eqs. S7-S8**). To estimate the production of macrophages based on the concentration of IL-6, we used measurements for the production of macrophages by fibroblasts. Fibroblasts release IL-6, which then up-regulates the expression of functional M-CSF receptors on monocytes[16] and allows monocytes to consume autocrine M-CSF and thus switch differentiation to macrophages rather than DCs. Chomarat et al.[16] cultured monocytes in GM-CSF and IL-4 with graded numbers of normal skin fibroblasts. At day 5, cells were analyzed for macrophage markers CD1a and CD14. Fitting **Eq. S24** to these results gave an $EC_{50} = 61.6$ cells and $h = 1.96$ (**S1E Fig**). Using then concentration of IL-6 produced by 250,000 fibroblasts (4.5pg/ml) and assuming that there is a linear relationship between the number of fibroblasts and the concentration of IL-6, we converted this to an unbound IL-6 concentration, i.e. $EC_{50} = 1.1 \times 10^3$ pg/ml. Scaling this by 10^{-5} (the order of initial bound to unbound IL-6 in the model) gives the bound IL-6 concentration $\epsilon_{L,M\Phi} = 0.011$ pg/ml.

S4.1.5. Effect of IFN on CD8+ T cells

To estimate the half-effect IFN concentration for CD8+ T cell regulation, $\epsilon_{F,T}$, we used dose-response measurements for CD8+ T regulation by IFN-γ. IFN-γ is known to regulate CD8+ T cell differentiation through co-stimulation of the signal transducer and activator of transcription 1 (STAT1) pathway[19]. Krummel et al.[19] measured the effect on IFN-γ signaling in CD8+ T cells *in vitro* by analyzing the p(Y701) STAT1 as a function of increasing concentrations. Fitting **Eq. S24** to this data for EC_{50} and h resulting in a h value of approximately 1, so we fixed $h = 1$ to improve identifitability and fit EC_{50} which gave $EC_{50} =$ 0.4 ng/ml. Assuming the half-effect concentration for IFN-γ regulation of the STAT1 pathway can be used to estimate the half-effect concentration of IFN gives $\epsilon_{F,T} = 0.004$ pg/ml (scaled by 10^{-5} to obtain the unbound concentration; **S1F Fig**).

S4.1.6. Effect of IL-6 on CD4+ T cell expansion

IL-6 stimulates IL-2 production and the proliferation of CD8+ and CD4+ T cells[20]. Holsti and Raulet[20] measured the counts per minute (CPM) of CD4+ cell proliferation from IL-6 and IL-1 induction. Converting their data from sample dilution to μg/ml and fitting **Eq. S24** gave $h = 2$ and $EC_{50} = 2.26 \times 10^{-4}$ (reciprocal dilution of IL-6) (**S2A Fig**). In Holsti and Raulet, the medium contained 2.1 μg/ml of IL-6. Converting the dilution to a concentration in our units and scaling this by 10^{-5} (the order of initial bound IL-6 to unbound IL-6) gives $\epsilon_{L,T} = 4.7 \times 10^{-3}$ pg/ml.

S4.2. Estimating parameters from temporal data

S4.2.1. Proliferation rate of epithelial cells

We used measurements from Lawal *et al.*[2] of the number of A549 cells *in vitro* grown over 4 days using Vi-CELL XR Cell Viability Analyser to fit an exponential growth curve and determined the proliferation rate of epithelial cells to be $\lambda_S = 0.744$/day (**S2B Fig**).

S4.2.2. IL-6 internalization rate

Bound IL-6 is internalized at a rate $k_{int_L} L_B$ (**Eq. S12**), which gives the fraction of internalized IL-6

$$f(t) = 1 - e^{-k_{int_L} t}. \qquad S30$$

Nesbitt and Fuller[46] measured the fraction of IL-6 internalized by hepatocytes *in vitro* by incubating IL-6 with the cells at 4°C and then removing unbound IL-6 by washing and then incubating in prewarmed 37°C binding medium for the various time periods. Fitting k_{int_L} to the fraction of IL-6 internalized by hepatocytes over 30 minutes[46] gave $k_{int_L} = 61.8$/day (**S2C Fig**).

S4.2.3. Neutrophil-induced cell death rate

To estimate the rate of epithelial cell death induced by the release of H_2O_2 by neutrophils (δ_N), we used *in vitro* measurements (using flow cytometry) of the total alveolar macrophages apoptosis after H_2O_2 exposure from 0-12 hours[23] and fit an exponential decay rate, which gave $\delta_N = 1.68$/day (**S2D Fig**).

S4.2.4. Rate of phagocytosis of dead cells by macrophages

The rate macrophages phagocytose dead material is described by

$$\frac{dM_{\Phi E}}{dt} = -d_{D,M\Phi} D M_{\Phi E}, \tag{S31}$$

$$\frac{dM_{\Phi F}}{dt} = d_{D,M\Phi} D M_{\Phi E}, \tag{S32}$$

$$\frac{dD}{dt} = -d_{D,M\Phi} D (M_{\Phi E} - M_{\Phi F}), \tag{S33}$$

where empty macrophages ($M_{\Phi E}$) phagocytose dead cells (D) at a rate $d_{D,M\Phi}$ and become loaded macrophages ($M_{\Phi F}$). Loaded macrophages also phagocytose dead cells at a rate $d_{D,M\Phi}$. Assuming the initial concentration of macrophages and dead cells is $M_{\Phi E}(0) = 36 \times 10^7$ cells/ml, $M_{\Phi F}(0) = 0$ cells/ml, and $D(0) = 5 \times 36 \times 10^7$ cells/ml (based on our full model's initial conditions and the experiment where the percentage of macrophages that had engulfed material *in vitro* over 25 hours was measured[26]), we fit the rate macrophages phagocytose dead material over 25 hours and obtained $d_{D,M\Phi} = 8.03$ per cell/day (**S2E Fig**).

S4.2.5. Clearance of extracellular virus by macrophages

To determine the clearance rate of extracellular virus by macrophages, $\delta_{V,M\Phi}$, we used measurements of foot-and-mouth disease virus (FMDV) uptake in macrophages over 2 hours *in vitro*[22] with the simple model

$$\frac{dV}{dt} = -\delta_{V,M\Phi} V M_{\Phi I}, \tag{S34}$$

$$\frac{dV_M}{dt} = \delta_{V,M\Phi} V M_{\Phi I}, \tag{S35}$$

where V is free virus, and V_M is the amount of phagocytosed virus. Considering $M_{\Phi I} = 36 \times 10^7$ cells/ml (i.e. our initial measurements in the lung) was constant gave an estimate of $\delta_{V,M\Phi} = 768$/day (**S2F Fig**).

S4.2.6. Production of type I IFN by monocytes

We modelled the production of type I IFN by monocytes (**Eq. S17**) by

$$\frac{dF_U}{dt} = \frac{p_{F,M} M}{M + \eta_{F,M}}. \tag{S36}$$

To fit the production of IFN from monocytes, we considered a simple production function for monocytes in the absence of any cytokine or inflammatory signalling

$$\frac{dM}{dt} = \frac{p_{\widehat{M}} M^h}{M^h + EC_{50,\widehat{M}}^h}\left(1 - \frac{M}{\widehat{M}_{max}}\right), \tag{S37}$$

where $p_{\widehat{M}}$ is the rate of monocyte production per day, $EC_{50,\widehat{M}}$ is the production half-effect, h is the Hill coefficient, and $\widehat{M}_{max}$ is the carrying capacity of the monocyte population. Ohta et al.[41] measured the number of monocytes after incubation for 12 days with 0.1 nM of calcitriol. Fitting **Eq. S37** to their data, we obtained $EC_{50,\widehat{M}} = 5.4 \times 10^4$ cells, $h = 13.8$, and $\hat{p} = 9.4 \times 10^4$ cells/day (**S3A Fig**).

Krilov et al.[42] measured IFN-α production from monocytes that had been cultured for either 1, 2, 4 or 7 days before the introduction of respiratory syncytial virus (RSV). IFN-α was measured 24 hours after RSV was introduced at t_{RSV}. Combining **Eqs. S36-S37**, gives the production of IFN (F_U) from RSV stimulation of monocytes

$$\frac{d\hat{M}}{dt} = \frac{p\hat{M}^h}{M^h + EC^h_{50,\hat{M}}}\left(1 - \frac{\hat{M}}{M_{max}}\right), \tag{S38}$$

$$\frac{dF_U}{dt} = \frac{p_{F,M}M^h}{M^h + \eta^h_{F,M}} H(t - t_{RSV}), \tag{S39}$$

assuming IFN production occurs only after RVS introduction at t_{RSV}=1, 2, 4, or 7 days (modelled using the Heaviside function). Fixing $\eta_{F,M} = EC_{50,\hat{M}} = 0.54$ pg/ml, and setting the production rate of monocytes to be equivalent to Ohta's experiments (**S3A Fig**), we fit the concentration of IFN 24 hours after RSV is introduced ($F_U(t_{RSV} + 24)$) and obtained $p_{F,M} = 997.1978$ IU/ml/day (**S3B Fig**). Converting the production rate using IFN's specific activity of 0.028 IU/pg gives $p_{F,M} = 3.561$ pg/ml/day

S4.2.7. Resident macrophage production rate during declining infection

Tissue-resident (or alveolar) macrophages return to homeostasis after viral infections have been successfully cleared[85], which we accounted for using logistic production (**Eqs. S6-S7**):

$$\frac{dV}{dt} = -d_V V, \tag{S40}$$

$$\frac{dM_{\Phi R}}{dt} = \frac{\lambda_{M\Phi}M_{\Phi I}}{V + \epsilon_{V,M\Phi}}\left(1 - \frac{M_{\Phi R}}{M_{\Phi max}}\right), \tag{S41}$$

$$\frac{dM_{\Phi I}}{dt} = -d_{M\Phi}M_{\Phi I} - \frac{\lambda_{M\Phi}M_{\Phi I}}{V + \epsilon_{V,M\Phi}}\left(1 - \frac{M_{\Phi R}}{M_{\Phi max}}\right). \tag{S42}$$

Here $\lambda_{M\Phi}$ and $\epsilon_{V,M\Phi}$ were fit and all other parameters were fixed to their estimated values (**S1 Table**). We assumed that the resident macrophage production was independent of virus type, and instead depended primarily on viral load. As such, we used data from influenza A to estimate resident macrophage kinetics.

We infected mice with 75 $TCID_{50}$ influenza A/Puerto Rico/34/8 (PR8) and measured viral loads[86] and alveolar macrophages ($F480^{hi}CD11c^{hi}CD11B^-$, see **S3D-E Fig**). Fitting **Eqs. S40-S42** to this data (**S3D-E Fig**) resulted in estimates of $\lambda_{M\Phi} = 0.082$ $TCID_{50}$/day, $\epsilon_{V,M\Phi} = 63.1$ $TCID_{50}$, $M_{\Phi,max} = 5.02$ cells, and $d_V = 1.43$/day. To convert from $TCID_{50}$ to a viral copies (RNA copy number) per volume (ml) for the units in our model, we used correlations between these (**S3C Fig**) for influenza A matrix[87]. We assumed 3.5 $TCID_{50}$ was equivalent to 2.19×10^5 virus copies[88] and took the ratio between the $TCID_{50}$/100 μl and 10^6 copies/100 μl to be approximately 0.37 (**S3C Fig**). Thus, we set $\lambda_{M\Phi} = 5.94 \times 10^3$ copies/ml/day and $\epsilon_{V,M\Phi} = 905.22$ copies/ml in our simulations. We validated these estimates against data from Landsman and Jung[4] (not shown).

S4.2.8. Production of GM-CSF by monocytes

Lee et al.[39] measured the concentration of GM-CSF produced by adherent monocytes incubated with LPS over 72 hours (**S3F Fig**). To fit to this data, we developed a simplified submodel given by

$$\frac{dM}{dt} = \frac{p_{M,G}G_B^{h_M}}{G_B^{h_M} + \epsilon_{G,M}} - d_M M, \tag{S43}$$

$$\frac{dG_U}{dt} = \frac{p_{G,M}M}{M + \eta_{G,M}} - k_{lin_G}G_U - k_{B_G}(MA_G - G_B)G_U + k_{U_G}G_{B,}, \quad S44$$

$$\frac{dG_B}{dt} = -k_{int_G}G_B + k_{B_G}(MA_G - G_B)G_U - k_{U_G}G_B, \quad S45$$

where the production of monocytes (M) by bound GM-CSF (G_B) was modelled by a Hill function. Parameters were calibrated to homeostasis and we found the production rate of monocytes by GM-CSF to be $p_{M,G} = 7.29 \times 10^3$ cells/ml and the production of GM-CSF by monocytes to be $p_{G,M} = 7.7 \times 10^5$ pg/ml/day through fitting to the GM-CSF measurements of Lee et al.[39] (**S3F Fig**).

S4.2.9. Production of IFN by infected cells

To determine the production rate of type I IFN by infected cells, we considered all immune populations to be zero in the full model (**Eqs. S1-S22**), giving

$$\frac{dV}{dt} = pI - d_V V, \quad S46$$

$$\frac{dS}{dt} = \lambda_S\left(1 - \frac{S + I}{S_{max}}\right)S - \beta SV, \quad S47$$

$$\frac{dI}{dt} = \frac{\beta}{1 + F_B/\epsilon_{F,I}}S(t - \tau_I)V(t - \tau_I) - d_I I, \quad S48$$

$$\frac{dF_U}{dt} = \psi^*_{prod,F} + \frac{p_{F,I}I}{I + \eta_{F,I}} - k_{lin_F}F_U - k_{B_F}(IA_F - F_B)F_U + k_{U_F}F_B, \quad S49$$

$$\frac{dF_B}{dt} = -k_{int_F}F_B + k_{B_F}(IA_F - F_B)F_U - k_{U_F}F_B. \quad S50$$

Here IFN is only produced by infected cells and so there is an additional homeostatic production of IFN, $\psi^*_{prod,F}$, to account for general macrophage and monocyte production. $\psi^*_{prod,F}$ is obtained from calculating homeostasis for F_U and F_B, i.e. $dF_U/dt = dF_B/dt = 0$. Resistant cells (R) were not considered in this model as the data was only measured over 1 day. By fixing all parameters to their previously established values (**S1 Table**), and fitting $p_{F,I}$ and $\eta_{F,I}$, we obtained $p_{F,I} = 2.823 \times 10^4$ pg/ml/day and $\eta_{F,I} = 0.00112$ pg/ml (**S4A Fig**). Since the concentration of IFN-α in patients infected with SARS-CoV-2 is lower than IFN-β, we reduced the production rate to $p_{F,I} = 2.823$ pg/ml/day so that model dynamics lay within the ranges of IFN-α exhibited by patients with SARS-CoV-2 infection (**S7A-B Fig** and Laing et al.[79]).

S4.2.10. Production of IL-6 by infected cells

To determine the rate of production of IL-6 by infected cells, Ye *et al*[35] measured the *in vitro* replication kinetics of H5N1 and H7N9 viruses in A549 cells. Cells were infected by either virus at an MOI of 2 and grown to confluence in sterile T75-tissue culture flasks (approximate cell number of 8.4×10^6)[89] and the concentration of IL-6 released from A549 cells in response to infection with both viruses was measured. We reduced the full model (**Eqs. S1-S22**) to only consider virus infection and IL-6 production by infected cells

$$\frac{dV}{dt} = pI - d_V V, \quad S51$$

$$\frac{dS}{dt} = -\beta SV, \quad S52$$

$$\frac{dI}{dt} = \beta SV - d_I I, \qquad S53$$

$$\frac{dL_U}{dt} = \frac{p_{L,I} I}{I + \eta_{L,I}}. \qquad S54$$

and fit to this data to obtain $p_{L,I} = 11.887$ pg/ml and $\eta_{L,I} = 0.7232 \times 10^9$ cells/ml (**S4B-C Fig**).

S4.2.11. Production of IL-6 by alveolar macrophages

We modelled the production of IL-6 by alveolar macrophages by

$$\frac{dL_U}{dt} = \frac{p_{L,M_{\Phi I}} M_{\Phi \mathrm{I}}}{M_{\Phi I} + \eta_{L,M\Phi}} \qquad S55$$

to compare to observations from Shibata et al.[36] who measured the production of IL-6 by alveolar macrophages stimulated by different concentrations of LPS *in vitro*. Assuming no proliferation of macrophages from LPS introduction but that LPS scales the production of IL-6, we modified the above equation to be

$$\frac{dL_U}{dt} = \frac{p_{L,M_{\Phi I}} M_{\Phi \mathrm{I}}}{M_{\Phi I} + \eta_{L,M\Phi}} LPS, \qquad S56$$

and fit the Shibata et al. data and obtained $p_{L,M_{\Phi I}} = 0.078$ ng/ml/hour and $\eta_{L,M\Phi} = 4.47 \times 10^5$ cells/ml (**S4D Fig**).

S5. Parameters calculated from homeostasis

Remaining parameters in the model were estimated to ensure the model maintained homeostasis in the absence of infection, i.e. we required the system to return to equilibrium state after small perturbations in initial conditions for the immune cells and cytokines. Homeostasis equations are defined below (**Eqs. S57-S70**), along with the corresponding parameter they define. These were determined by solving $d/dt = 0$. At homeostasis we assume there to be no virus and resistant cells ($V = R = 0$). Here X^* represents homeostatic values.

$$F_B(0) = F_B^* = \frac{k_{B_F} T^* A_F F_U^*}{k_{int_F} + k_{B_F} F_U^* + k_{U_F}}, \qquad S57$$

$$G_B(0) = G_B^* = \frac{k_{B_G} M^* A_G G_U^*}{k_{int_G} + k_{B_G} G_U^* + k_{U_G}}, \qquad S58$$

$$C_B(0) = C_B^* = \frac{k_{B_C} {C_U^*}^{POW_C} A_C N^*}{k_{int_C} + k_{B_C} {C_U^*}^{POW_c} + k_{U_C}}, \qquad S59$$

$$C_{BF}(0) = C_{BF}^* = \frac{C_B^*}{A_C N^*}, \qquad S60$$

$$L_B(0) = L_B^* = \frac{k_{B_L}(T^* + N^* + M^*) A_L L_U^*}{k_{int_L} + k_{B_L} L_U^* + k_{U_L}}, \qquad S61$$

$$M_{\Phi I}(0) = M_{\Phi I}^* = \frac{\left(\frac{p_{M_{\Phi \mathrm{I}},G} {G_B^*}^{h_{M,M\Phi}} M^*}{{G_B^*}^{h_{M,M\Phi}} + \epsilon_{G,M\Phi}^{h_{M,M\Phi}}} + \frac{p_{M_{\Phi I},L} L_B^* M^*}{L_B^* + \epsilon_{L,M\Phi}} \right)}{\left(1 - \frac{M_{\Phi R}^*}{M_{\Phi max}}\right) \frac{\lambda_{M\Phi}}{\epsilon_{V,M\Phi}} + d_{M_{\Phi \mathrm{I}}}}, \qquad S62$$

$$\eta_{C,M} = \frac{p_{C,M}M^* - M^*\left(k_{lin_C}C_U^* + k_{B_C}(N^*A_C - C_B^*)C_U^{*POW_C} - k_{U_C}C_B^*\right)}{k_{lin_C}C_U^* + k_{B_C}(N^*A_C - C_B^*)C_U^{*POW_C} - k_{U_C}C_B^*}, \quad S63$$

$$p_{L,M\Phi} = \frac{M_{\Phi I}^* + \eta_{L,M\Phi}}{M_{\Phi I}^*}\left(-\frac{p_{L,M}M^*}{M^* + \eta_{L,M}} + k_{lin_L}L_U^* + k_{B_L}\left((N^* + T^* + M^*)A_L - L_B^*\right)L_U^* - k_{U_L}L_B^*\right), \quad S64$$

$$p_{G,M\Phi_I} = \frac{k_{lin_G}G_U^* + k_{B_G}(M^*A_G - G_B^*)G_U^* - k_{U_G G_B^*}}{\frac{M_{\Phi I}^*}{M_{\Phi I}^* + \eta_{G,M\Phi}} + \frac{M^*}{M^* + \eta_{G,M}}}, \quad S65$$

$$p_{M,G} = \left(\frac{G_B^{*h_M} + \epsilon_{G,M}^{h_M}}{G_B^{*h_M}}\right)\left(\frac{p_{M_{\Phi I},G}G_B^{*h_{M,M\Phi}}M^*}{G_B^{*h_{M,M\Phi}} + \epsilon_{G,M\Phi}^{h_{M,M\Phi}}} + \frac{p_{M_{\Phi I},L}L_B^*M^*}{L_B^* + \epsilon_{L,M\Phi}} + d_M M^*\right), \quad S66$$

$$\eta_{F,M\Phi} = \frac{p_{F,M\Phi}M_{\Phi I}^* + \left(\frac{p_{F,M}M^*}{M^* + \eta_{F,M}} - k_{lin_F}F_U^* - k_{B_F}(T^*A_F - F_B^*)F_U^* + k_{U_F}F_B^*\right)M_{\Phi I}^*}{-\frac{p_{F,M}M^*}{M^* + \eta_{F,M}} + k_{lin_F}F_U^* + k_{B_F}(T^*A_F - F_B^*)F_U^* - k_{U_F}F_B^*}, \quad S67$$

$$T_{prod}^* = d_T T^* - \frac{p_{T,L}L_B^*T^*}{L_B^* + \epsilon_{L,T}} - \frac{p_{T,F}F_B^*T^*}{F_B^* + \epsilon_{F,T}}, \quad S68$$

$$N_{prod}^* = \left(d_N N^* - \frac{p_{N_L}L_B^*}{L_B^* + \epsilon_{L,N}}\right)\frac{1}{NR}, \quad S69$$

$$M_{prod}^* = \frac{\frac{1}{MR}\left(\frac{p_{M_{\Phi I},G}G_B^{*h_{M,M\Phi}}M^*}{G_B^{*h_{M,M\Phi}} + \epsilon_{G,M\Phi}^{h_{M,M\Phi}}} + \frac{p_{M_{\Phi I},L}L_B^*M^*}{L_B^* + \epsilon_{L,M}} + d_M M^*\right) - \psi_M^{max}\frac{G_B^{*h_M}}{G_B^{*h_M} + \epsilon_{G,M}^{h_M}}}{1 - \frac{G_B^{*h_M}}{G_B^{*h_M} + \epsilon_{G,M}^{h_M}}}, \quad S70$$

The model was then simulated to confirm the parameter values determined resulted in a stable system at homeostasis (**S5** Fig).

S6. Model prediction, validation and sensitivity analysis

S6.1. Model validation against viral load in macaques

To validate the model's ability to replicate viral dynamics and the estimates we obtained for the viral parameters based on the human viral loads (**Fig 2**), we fit the submodel **Eqs. 6-9** to viral load measurements in macaques (**S6 Fig**). Munster *et al.* [90] measured SARS-CoV-2 viral loads in eight adult rhesus macaques inoculated with 4×10^5 $TCID_{50}$/ml (3×10^8 genome/ml) SARS-CoV-2. We obtained estimates of $\beta = 0.29\ \text{day}^{-1}(\log_{10}(\text{cop/ml}))^{-1}$, $p = 741\ \text{day}^{-1}\log(\text{cop/ml})\ (10^9\text{cells})^{-1}$, $d_I = 0.14\ \text{day}^{-1}, V_0 = 12\ \log_{10}(\text{cop/ml})$ and $d_V = 18.94\ \text{day}^{-1}$. These estimates were used as seeds for fitting the human viral load measurements to **Eqs. 6-9**.

S6.2. Model validation against human COVID-19 disease responses

To validate that the predicted dynamics from the reduced IFN model (**Eqs. 31-37** and **Fig 3**, **Main Text**) qualitatively matched IFN dynamics in humans, we plotted the model simulated against IFN-α2

concentrations in COVID-19 patients (n=26) determined by single-molecule array (Simoa) by Trouillet-Assant et al.[68] (**S7A Fig**). Measurements were reported as days from symptom onset. Of these patients, 21 returned positive IFN-$\alpha 2$ measurements (IFN-known) and 5 returned no IFN-$\alpha 2$ measurements (IFN-unknown) and these patients had poorer outcomes[68].

We next sought to further validate predictions of the cytokine dynamics of the full model (**Fig 4**) by comparing IFN, IL-6, and G-CSF dynamics to previously published observations of these kinetics in humans (**S7B-F Fig**). For this, the measurements described above for IFN-$\alpha 2$ plasma concentration from Trouillet-Assant et al.[68] (**S7B Fig**) were used to validate the mild and severe IFN dynamics in our model. Trouillet-Assant et al. also measured corresponding IL-6 plasma concentrations from critically ill patients (n=26) using a multiplexed assay with the Ella platform in IFN-negative and IFN-positive patients. Since IFN-negative patients were noted to exhibit poor disease outcomes, we used the distinction in IL-6 measurements of IFN-negative and IFN-positive to validate the distinction between mild and severe disease simulations of IL-6 in our model (**S7C Fig**). To further validate the IL-6 dynamics of our model, we used the IL-6 plasma concentration in patients requiring and not requiring mechanical ventilation obtained using Elecsys IL-6 immunoassay by Herold et al.[91] (**S7D Fig**). We assumed patients requiring mechanical ventilation were exhibiting severe disease and those not requiring mechanical ventilation were exhibiting mild disease. Using moderate (n=80) or severe (n=33) COVID-19 patients and health care workers (HCW; n=108) donor samples, Lucas *et al.*[92] quantified the concentration of IL-6 by ELISA. We also used these measurements to validate the IL-6 concentration (**S7E Fig**).G-CSF plasma concentrations in symptomatic (n=37) and asymptomatic (n=37) patients were obtained using assays collected in the acute phase during hospitalization of COVID-19 patients by Long et al.[93] (**S7F Fig**). These measurements were used to validate the mild and severe dynamics of IL-6 and G-CSF in our full model.

To validate predictions of the immune cell dynamics in the full model (**Fig 4**), we used Lucas *et al.*'s reported concentrations for neutrophils, monocytes and CD8$^+$ T cell in moderate and severe COVID-19 patients and healthy HCW[92]. Using moderate (n=80) or severe (n=33) COVID-19 patients and HCW (n=108) donor samples, Lucas *et al.*[92] quantified the number of leukocytes using flow cytometry. Normalising the moderate and severe COVID-19 patients by the average HCW measurement, we validated the change in disease dynamics of our full model (**S7G-I Fig**). Full model simulations are given in **S8 Fig**.

S6.3. Model sensitivity to changes in parameters and immune cell knockdown

To better understand the robustness of the model's predictions, we performed a local sensitivity analysis for all parameters (**Eqs. S1-S22**) by individually varying each parameter by $\pm 20\%$ from its estimated value, and quantifying the effect on the model's output. The change in output was recorded and used to evaluate different point metrics representing the inflammatory response to SARS-CoV-2:

- maximum viral load: $\max_t(V(t))$,
- maximum number of dead cells: $\max_t(D(t))$,
- minimum uninfected tissue: $\min_t\big(S(t) + R(t)\big)$,
- maximum number of inflammatory macrophages: $\max_t\big(M_{\Phi I}(t)\big)$,
- maximum number of CD8$^+$ T cells: $\max_t\big(T(t)\big)$,
- maximum unbound IL-6: $\max_t\big(L_U(t)\big)$,
- maximum unbound IFN: $\max_t\big(F_U(t)\big)$,
- total exposure (area under the curve) to type I IFN,
- duration of tissue damage under 80%: $t_u - t_l$, where

- t_u is the first time point for which $S(t_u) + R(t_u) < 0.8 \times S_{max}$,
- t_l is the first time point where $S(t_u) + R(t_u) \geq 0.8 \times S_{max}$, and $t_l > t_u$,

- peak of unbound type I IFN: time t_p, when $F_U(t_p) = \max_t(F_U(t))$.

We then determined the maximum increase and decrease for a particular metric (see the table in **Fig 5**). **S9 Fig** reports the results of the sensitivity analysis for all model parameters (extension of **Fig 5** in the main text).

This local sensitivity analysis showed the model is robust to perturbations for a large majority of parameters. The most significant changes in model output were due to changes in IFN-, IL-6- and virus related parameters, which led to the selection of subsets of these sensitive parameters for the generation of our virtual cohort to interrogate on the causes driving responses for the most sensitive parameters.

To further analyze the robustness of the model to major immunological changes, we simulated the effects of complete removal (knockout) of either neutrophil, monocyte, or macrophages using the mild disease parameters (**Fig 7 Main Text**), given that systemic dysregulation is already characteristic of severe disease (**S10 Fig**).

S6.4. Generating virtual patients

Initial parameter sets for each virtual patient were drawn from normal distributions with means fixed to the corresponding parameter value in **S1 Table** and standard deviations derived from appropriate standard deviation or confidence interval measurements in the literature. Specifically, the standard deviation for

- the half-effect concentration of IFN on viral infectivity ($\epsilon_{F,I}$) was informed by the 95% confidence interval from fitting the Emax curve to MERS-CoV-expression nanoluciferase (nLUC) reported by Sheahan et al.[15], from the IFN-α 95% confidence interval on day 0 from Trouillet-Assant et al.[68] (**S7B-C Fig**),
- IFN production by infected cells ($p_{F,I}$ and $\eta_{F,I}$) and IFN production by macrophages ($\eta_{F,M\Phi}$) were drawn from IL-6 concentration in no mechanical ventilation patients (mild) and mechanical ventilation patients (severe) from Herold et al.[91],
- the production of IL-6 by macrophages and macrophages by IL-6 ($p_{L,M\Phi}$, and $p_{M\Phi,L}$) from Liu et al. [94] (**S7D Fig**), and
- the production of monocytes by infected cells ($p_{M,I}$), and from 95% confidence interval generated from estimating the parameter for production of IFN by monocytes[41] ($p_{F,M}$; **S3 Fig**).

From normal distributions with standard deviation described above and mean as the original parameter values ($\hat{p}$), we then generated normal distributions covering 99.7% of values lying with 3 standard deviations of the mean, i.e., $[\mu - 3\sigma, \mu + 3\sigma]$[95].

After drawing an initial patient parameter set for each patient, we next used simulated annealing to determine a parameter set that resulted in patient dynamics within physiological ranges[96] for $[l_i, u_i]$ by minimising **Eq. 15**, where l_i and u_i are the upper and lower bounds extracted from measurements for viral load, type I IFN, G-CSF, and IL-6 (**Fig 7 Main Text**). Parameters in the simulated annealing optimization were bounded above by $\mu + 5\sigma$ and below by $\max(0, \mu - 5\sigma)$. The resulting parameter set from this optimization was then considered to represent a realistic patient and they were accepted into the cohort. Posterior distributions for the 200 virtual patients are provided in **S11 Fig**.

To compare how average parameter values in the cohort deviate from the mean of the initial normal distribution, we plotted the average of virtual cohort and compared it to the cohort's distribution (**S11 Fig**). For the most part, the average of the cohort was similar to that of the underlying distribution used for sampling, with a few exceptions. As the average of the underlying parameter distribution was taken from the fitted values from our initial model calibration (which we took to represent an average mild response), it is perhaps not surprising that the average of the virtual cohort (which encompasses both mild and severe patients) deviates from the point estimate representing a mild/average parameter value. The viral, IFN, IL-

6, and G-CSF dynamics of the cohort are seen in **S12 Fig**, with the physiological ranges used for optimization.

Supplementary References

1. Goyal, A., Cardozo-Ojeda, E. F. & Schiffer, J. T. Potency and timing of antiviral therapy as determinants of duration of SARS-CoV-2 shedding and intensity of inflammatory response. *Sci. Adv.* **6**, eabc7112 (2020).
2. Lawal, O. *et al.* Volatile organic compound signature from co-culture of lung epithelial cell line with: Pseudomonas aeruginosa. *Analyst* **143**, 3148–3155 (2018).
3. Crapo, J. D., Barry, B. E., Gehr, P., Bachofen, M. & Weibel, E. R. Cell number and cell characteristics of the normal human lung. *Am. Rev. Respir. Dis.* **126**, 332–337 (1982).
4. Landsman, L. & Jung, S. Lung Macrophages Serve as Obligatory Intermediate between Blood Monocytes and Alveolar Macrophages. *J. Immunol.* **179**, 3488–3494 (2007).
5. Ng, M. L., Tan, S. H., See, E. E., Ooi, E. E. & Ling, A. E. Proliferative growth of SARS coronavirus in Vero E6 cells. *J. Gen. Virol.* **84**, 3291–3303 (2003).
6. Norman, P. S. *Immunobiology: The immune system in health and disease*. *Journal of Allergy and Clinical Immunology* vol. 96 (Garland Pub., 1995).
7. Kratofil, R. M., Kubes, P. & Deniset, J. F. Monocyte conversion during inflammation and injury. *Arterioscler. Thromb. Vasc. Biol.* **37**, 35–42 (2017).
8. Jenner, A. L., Frascoli, F., Yun, C. O. & Kim, P. S. Optimising hydrogel release profiles for viro-immunotherapy using oncolytic adenovirus expressing IL-12 and GM-CSF with immature dendritic cells. *Appl. Sci.* **10**, 2872 (2020).
9. Lee, J., Adler, F. R. & Kim, P. S. A Mathematical Model for the Macrophage Response to Respiratory Viral Infection in Normal and Asthmatic Conditions. *Bull. Math. Biol.* **79**, 1979–1998 (2017).
10. Pawelek, K. A., Dor, D., Salmeron, C. & Handel, A. Within-host models of high and low pathogenic influenza virus infections: The role of macrophages. *PLoS One* **11**, 2016 (2016).
11. Zhang, N. & Bevan, M. J. CD8+ T Cells: Foot Soldiers of the Immune System. *Immunity* **35**, 161–168 (2011).
12. Baral, S., Antia, R. & Dixit, N. M. A dynamical motif comprising the interactions between antigens and CD8 T cells may underlie the outcomes of viral infections. *Proc. Natl. Acad. Sci. U. S. A.* **116**, 17393–17398 (2019).
13. Cassidy, T., Humphries, A. R., Craig, M. & Mackey, M. C. Characterizing Chemotherapy-Induced Neutropenia and Monocytopenia Through Mathematical Modelling. *Bull. Math. Biol.* **82**, 2020.04.02.022046 (2020).
14. Craig, M., Humphries, A. R. & Mackey, M. C. A Mathematical Model of Granulopoiesis Incorporating the Negative Feedback Dynamics and Kinetics of G-CSF/Neutrophil Binding and Internalization. *Bull. Math. Biol.* **78**, 2304–2357 (2016).
15. Sheahan, T. P. *et al.* Comparative therapeutic efficacy of remdesivir and combination lopinavir, ritonavir, and interferon beta against MERS-CoV. *Nat. Commun.* **11**, 222 (2020).
16. Chomarat, P., Banchereau, J., Davoust, J. & Palucka, A. K. IL-6 switches the differentiation of monocytes from dendritic cells to macrophages. *Nat. Immunol.* **1**, 510–514 (2000).
17. Sun, L. *et al.* GM-CSF Quantity Has a Selective Effect on Granulocytic vs. Monocytic Myeloid Development and Function. *Front. Immunol.* **9**, 1922 (2018).
18. Chen, B. D. M., Clark, C. R. & Chou -h., T. Granulocyte/macrophage colony-stimulating factor stimulates monocyte and tissue macrophage proliferation and enhances their responsiveness to macrophage colony-stimulating factor. *Blood* **71**, 997–1002 (1988).
19. Krummel, M. F. *et al.* Paracrine costimulation of IFN-γ signaling by integrins modulates CD8 T cell differentiation. *Proc. Natl. Acad. Sci. U. S. A.* **115**, 11585–11590 (2018).
20. Holsti, M. A. & Raulet, D. H. IL-6 and IL-1 synergize to stimulate IL-2 production and

proliferation of peripheral T cells. *J. Immunol.* **143**, 2514–2519 (1989).
21. Knaapen, A. M. *et al.* Neutrophils cause oxidative DNA damage in alveolar epithelial cells. *Free Radic. Biol. Med.* **27**, 234–240 (1999).
22. Rigden, R. C., Carrasco, C. P., Summerfield, A. & McCullough, K. C. Macrophage phagocytosis of foot-and-mouth disease virus may create infectious carriers. *Immunology* **106**, 537–548 (2002).
23. Yoshioka, Y., Kitao, T., Kishino, T., Yamamuro, A. & Maeda, S. Nitric Oxide Protects Macrophages from Hydrogen Peroxide-Induced Apoptosis by Inducing the Formation of Catalase. *J. Immunol.* **176**, 4675–4681 (2006).
24. Smith, P. K., Wang, S. Z., Dowling, K. D. & Forsyth, K. D. Leucocyte populations in respiratory syncytial virus-induced bronchiolitis. *J. Paediatr. Child Health* **37**, 146–151 (2001).
25. Lee, H. Y. *et al.* Simulation and Prediction of the Adaptive Immune Response to Influenza A Virus Infection. *J. Virol.* **83**, 7151–7165 (2009).
26. Klöditz, K. & Fadeel, B. Three cell deaths and a funeral: macrophage clearance of cells undergoing distinct modes of cell death. *Cell Death Discov.* **5**, 1–9 (2019).
27. Zent, C. S. & Elliott, M. R. Maxed out macs: physiologic cell clearance as a function of macrophage phagocytic capacity. *FEBS J.* **284**, 1021–1039 (2017).
28. Vargas, E. A. H. & Velasco-Hernandez, J. X. In-host Modelling of COVID-19 Kinetics in Humans. *medRxiv* 2020.03.26.20044487 (2020) doi:10.1101/2020.03.26.20044487.
29. Elmore, S. Apoptosis: A Review of Programmed Cell Death. *Toxicol. Pathol.* **35**, 495–516 (2007).
30. Ginhoux, F. & Guilliams, M. Tissue-Resident Macrophage Ontogeny and Homeostasis. *Immunity* **44**, 439–449 (2016).
31. Eftimie, R. & Eftimie, G. Tumour-associated macrophages and oncolytic virotherapies: a mathematical investigation into a complex dynamics. *Lett. Biomath.* **5**, S6–S35 (2018).
32. Patel, A. A. *et al.* The fate and lifespan of human monocyte subsets in steady state and systemic inflammation. *J. Exp. Med.* **214**, 1913–1923 (2017).
33. Craig, M., Humphries, A. R. & Mackey, M. C. An upper bound for the half-removal time of neutrophils from circulation. *Blood* **128**, 1989–1991 (2016).
34. Kim, P. S., Lee, P. P. & Levy, D. Modeling regulation mechanisms in the immune system. *J. Theor. Biol.* **246**, 33–69 (2007).
35. Ye, S., Lowther, S. & Stambas, J. Inhibition of Reactive Oxygen Species Production Ameliorates Inflammation Induced by Influenza A Viruses via Upregulation of SOCS1 and SOCS3. *J. Virol.* **89**, 2672–2683 (2015).
36. Shibata, Y. *et al.* GM-CSF regulates alveolar macrophage differentiation and innate immunity in the lung through PU.1. *Immunity* **15**, 557–567 (2001).
37. Alderson, M. R., Tough, T. W., Ziegler, S. F. & Grabstein, K. H. Interleukin 7 induces cytokine secretion and tumoricidal activity by human peripheral blood monocytes. *J. Exp. Med.* **173**, 923–930 (1991).
38. Hallsworth, M. P., Soh, C. P. C., Lane, H. J., Arm, E. P. & Lee, M. H. Selective enhancement of GM-CSF,TNF-α,lL-1β andIL-8 production by monocytes and macrophages of asthmatic subjects. *Eur. Respir. J.* **7**, 1096–1102 (1994).
39. Lee, M. T., Kaushansky, K., Ralph, P. & Ladner, M. B. Differential expression of M-CSF, G-CSF, and GM-CSF by human monocytes. *J. Leukoc. Biol.* **47**, 275–282 (1990).
40. Ioannidis, I., Ye, F., McNally, B., Willette, M. & Flano, E. Toll-Like Receptor Expression and Induction of Type I and Type III Interferons in Primary Airway Epithelial Cells. *J. Virol.* **87**, 3261–3270 (2013).
41. Ohta, M., Okabe, T., Ozawa, K., Urabe, A. & Takaku, F. 1α,25-Dihydroxy vitamin D3 (calcitriol) stimulates proliferation of human circulating monocytes in vitro. *FEBS Lett.* **185**, 9–13 (1985).
42. Krilov, L. R., Hendry, R. M., Godfrey, E. & McIntosh, K. Respiratory virus infection of peripheral blood monocytes: Correlation with ageing of cells and interferon production in vitro. *J. Gen. Virol.* **68**, 1749–1753 (1987).
43. Mehra, R. *et al.* Soluble interleukin 6 receptor: A novel marker of moderate to severe sleep-related

breathing disorder. *Arch. Intern. Med.* **166**, 1725–1731 (2006).
44. Stagg, J., Wu, J. H., Bouganim, N. & Galipeau, J. Granulocyte-macrophage colony-stimulating factor and interleukin-2 fusion cDNA for cancer gene immunotherapy. *Cancer Res.* **64**, 8795–8799 (2004).
45. Arnaud, P. Different interferons: Pharmacology, pharmacokinetics, proposed mechanisms, safety and side effects. *Rev. Med. Interne* **23**, 449S-458S (2002).
46. Nesbitt, J. E. & Fuller, G. M. Dynamics of interleukin-6 internalization and degradation in rat hepatocytes. *J. Biol. Chem.* **267**, 5739–5742 (1992).
47. Nicola, N. A., Peterson, L., Hilton, D. J. & Metcalf, D. Cellular processing of murine colony-stimulating factor (Multi-CSF, GM-CSF, G-CSF) receptors by normal hemopoietic cells and cell lines. *Growth Factors* **1**, 41–49 (1988).
48. Mager, D. E. & Jusko, W. J. Receptor-mediated pharmacokinetic/pharmacodynamic model of interferon-β 1a in humans. *Pharm. Res.* **19**, 1537–1543 (2002).
49. Tenhumberg, S. *et al.* Structure-guided optimization of the interleukin-6 trans-signaling antagonist sgp130. *J. Biol. Chem.* **283**, 27200–27207 (2008).
50. Snyers, L., Fontaine, V. & Content, J. Modulation of Interleukin-6 Receptors in Human Cells. *Ann. N. Y. Acad. Sci.* **557**, 388–395 (1989).
51. Taga, T., Kawanishi, Y., Hardy, R. R., Hirano, T. & Kishimoto, T. Receptors for b cell stimulatory factor 2: Quantitation, specificity, distribution, and regulation of their expression. *J. Exp. Med.* **166**, 967–981 (1987).
52. Yamaguchi, M. *et al.* Down-regulation of interleukin 6 receptors of mouse myelomonocytic leukemic cells by leukemia inhibitory factor. *J. Biol. Chem.* **267**, 22035–22042 (1992).
53. Chiba, S. *et al.* Characterization and molecular features of the cell surface receptor for human granulocyte-macrophage colony-stimulating factor. *Leukemia* **4**, 29–36 (1990).
54. Barreda, D. R., Hanington, P. C. & Belosevic, M. Regulation of myeloid development and function by colony stimulating factors. *Dev. Comp. Immunol.* **28**, 509–554 (2004).
55. Branca, A. A. Interferon receptors. *Vitr. Cell. Dev. Biol. - Anim.* **24**, 155–165 (1988).
56. Constantinescu, S. N. *et al.* Role of interferon α/β receptor chain 1 in the structure and transmembrane signaling of the interferon α/β receptor complex. *Proc. Natl. Acad. Sci. U. S. A.* **91**, 9602–9606 (1994).
57. Recombinant human interleukin-6 (with HSA). vol. 2020.
58. Razelle, K. Granulocyte-macrophage colony-stimulating factor. *Holland-Frei Cancer Medicine* vol. 6 (2003).
59. IFN-beta recombinant protein :: Interferon-beta 1a Recombinant Protein. vol. 2020.
60. Armstrong, J. D., Gluck, E. H., Crapo, R. O., Jones, H. A. & Hughes, J. M. B. Lung tissue volume estimated by simultaneous radiographic and helium dilution methods. *Thorax* **37**, 676–679 (1982).
61. CHAFFEY, N. Alberts, B., Johnson, A., Lewis, J., Raff, M., Roberts, K. and Walter, P. Molecular biology of the cell. 4th edn. *Ann. Bot.* **91**, 401–401 (2003).
62. Uppal, S. S., Verma, S. & Dhot, P. S. Normal Values of CD4 and CD8 Lymphocyte Subsets in Healthy Indian Adults and the Effects of Sex, Age, Ethnicity, and Smoking. *Cytom. Part B - Clin. Cytom.* **52**, 32–36 (2003).
63. Gordon, C. L. *et al.* Tissue reservoirs of antiviral T cell immunity in persistent human CMV infection. *J. Exp. Med.* **214**, 651–667 (2017).
64. Maes, M. *et al.* Elevated serum interleukin-6 (IL-6) and IL-6 receptor concentrations in posttraumatic stress disorder following accidental man-made traumatic events. *Biol. Psychiatry* **45**, 833–839 (1999).
65. Lee, J., Kim, Y., Lim, J., Kim, M. & Han, K. G-CSF and GM-CSF concentrations and receptor expression in peripheral blood leukemic cells from patients with chronic myelogenous leukemia. *Ann. Clin. Lab. Sci.* **38**, 331–337 (2008).
66. Suzuki, K. *et al.* Circulating sytokines and hormones with immunosuppressive but neutrophil-priming potentials rise after endurance exercise in humans. *Eur. J. Appl. Physiol.* **81**, 281–287

(2000).
67. Rodero, M. P. *et al.* Detection of interferon alpha protein reveals differential levels and cellular sources in disease. *J. Exp. Med.* **214**, 1547–1555 (2017).
68. Trouillet-Assant, S. *et al.* Type I IFN immunoprofiling in COVID-19 patients. *J. Allergy Clin. Immunol.* **146**, 206-208.e2 (2020).
69. Kasperska-Zajac, A., Sztylc, J., Machura, E. & Jop, G. Plasma IL-6 concentration correlates with clinical disease activity and serum C-reactive protein concentration in chronic urticaria patients. *Clin. Exp. Allergy* **41**, 1386–1391 (2011).
70. Sharma, R. & Sharma, S. Physiology, Blood Volume. in *StatPearls [Internet]* (StatPearls Publishing, 2018).
71. Cose, S., Brammer, C., Khanna, K. M., Masopust, D. & Lefrançois, L. Evidence that a significant number of naive T cells enter non-lymphoid organs as part of a normal migratory pathway. *Eur. J. Immunol.* **36**, 1423–1433 (2006).
72. Mager, D. & Jusko, W. Development of translational pharmacokinetic--pharmacodynamic models. *Clin. Pharmacol. Ther.* **83**, 909–912 (2008).
73. de Pillis, L. *et al.* Mathematical model creation for cancer chemo-immunotherapy. *Comput. Math. Methods Med.* **10**, 165–184 (2009).
74. Tura, S., Cavo, M. & Zinzani, P. L. *Hematology. Pathophysiology, Diagnosis and Treatment.* (2018).
75. Jones, G. Fitting and handling dose response data. *J. Comput. Aided. Mol. Des.* **29**, 1–11 (2015).
76. Macdougall, J. Analysis of dose--response studies—E max model. in *Dose finding in drug development* 127–145 (2006).
77. Zhu, Z. *et al.* From SARS and MERS to COVID-19: a brief summary and comparison of severe acute respiratory infections caused by three highly pathogenic human coronaviruses. *Respir. Res.* **21**, 1–14 (2020).
78. Smith, R. J. & Bowman, B. J. Generation of hydrogen peroxide by human neutrophils: Effects of soluble stimuli and requirements for divalent cations. *Int. J. Immunopharmacol.* **4**, 321 (1982).
79. Laing, A. G. *et al.* A dynamic COVID-19 immune signature includes associations with poor prognosis. *Nat. Med.* **26**, 1623–1635 (2020).
80. Weiss, S. J., Young, J., LoBuglio, A. F., Slivka, A. & Nimeh, N. F. Role of hydrogen peroxide in neutrophil-mediated destruction of cultured endothelial cells. *J. Clin. Invest.* **68**, 714–721 (1981).
81. Shi, Y. *et al.* Granulocyte-macrophage colony-stimulating factor (GM-CSF) and T-cell responses: what we do and don't know. *Cell Res.* **16**, 126–133 (2006).
82. Rösler, B. & Herold, S. Lung epithelial GM-CSF improves host defense function and epithelial repair in influenza virus pneumonia—a new therapeutic strategy? *Mol. Cell. Pediatr.* **3**, 29 (2016).
83. Pignatti, P. *et al.* High circulating levels of biologically inactive IL-6/SIL-6 receptor complexes in systemic juvenile idiopathic arthritis: Evidence for serum factors interfering with the binding to gp130. *Clin. Exp. Immunol.* **131**, 355–363 (2003).
84. Morris, G. E. *et al.* Agonists of toll-like receptors 2 and 4 activate airway smooth muscle via mononuclear leukocytes. *Am. J. Respir. Crit. Care Med.* **171**, 814–822 (2005).
85. Denney, L. & Ho, L. P. The role of respiratory epithelium in host defence against influenza virus infection. *Biomed. J.* **41**, 218–233 (2018).
86. Smith, A. P., Moquin, D. J., Bernhauerova, V. & Smith, A. M. Influenza virus infection model with density dependence supports biphasic viral decay. *Front. Microbiol.* **9**, 1554 (2018).
87. Laurie, K. L. *et al.* Interval between Infections and Viral Hierarchy Are Determinants of Viral Interference Following Influenza Virus Infection in a Ferret Model. *J. Infect. Dis.* **212**, 1701–1710 (2015).
88. Cao, P. *et al.* Innate Immunity and the Inter-exposure Interval Determine the Dynamics of Secondary Influenza Virus Infection and Explain Observed Viral Hierarchies. *PLoS Comput. Biol.* **11**, (2015).
89. Lam, W., Yeung, A. C., Chu, I. M. & Chan, P. K. S. Profiles of cytokine and chemokine gene

expression in human pulmonary epithelial cells induced by human and avian influenza viruses. *Virol. J.* **7**, 344 (2010).
90. Munster, V. J. *et al.* Respiratory disease in rhesus macaques inoculated with SARS-CoV-2. *Nature* **585**, 268–272 (2020).
91. Herold, T. *et al.* Elevated levels of IL-6 and CRP predict the need for mechanical ventilation in COVID-19. *J. Allergy Clin. Immunol.* **146**, 128-136.e4 (2020).
92. Lucas, C. *et al.* Longitudinal analyses reveal immunological misfiring in severe COVID-19. *Nature* **584**, 463–469 (2020).
93. Long, Q. X. *et al.* Clinical and immunological assessment of asymptomatic SARS-CoV-2 infections. *Nat. Med.* **26**, 1200–1204 (2020).
94. Liu, J. *et al.* Longitudinal characteristics of lymphocyte responses and cytokine profiles in the peripheral blood of SARS-CoV-2 infected patients. *EBioMedicine* **55**, 102763 (2020).
95. Cassidy, T. & Craig, M. Determinants of combination GM-CSF immunotherapy and oncolytic virotherapy success identified through in silico treatment personalization. *PLoS Comput. Biol.* **15**, (2019).
96. Allen, R. J., Rieger, T. R. & Musante, C. J. Efficient Generation and Selection of Virtual Populations in Quantitative Systems Pharmacology Models. *CPT Pharmacometrics Syst. Pharmacol.* **5**, 140–146 (2016).
