# Supplementary figures and images for: COVID-19 virtual patient cohort suggests immune mechanisms driving disease outcomes
Source: PLoS Pathog. 2021 Jul 14;17(7):e1009753. doi: 10.1371/journal.ppat.1009753 (PMC8312984; doi:10.1371/journal.ppat.1009753)

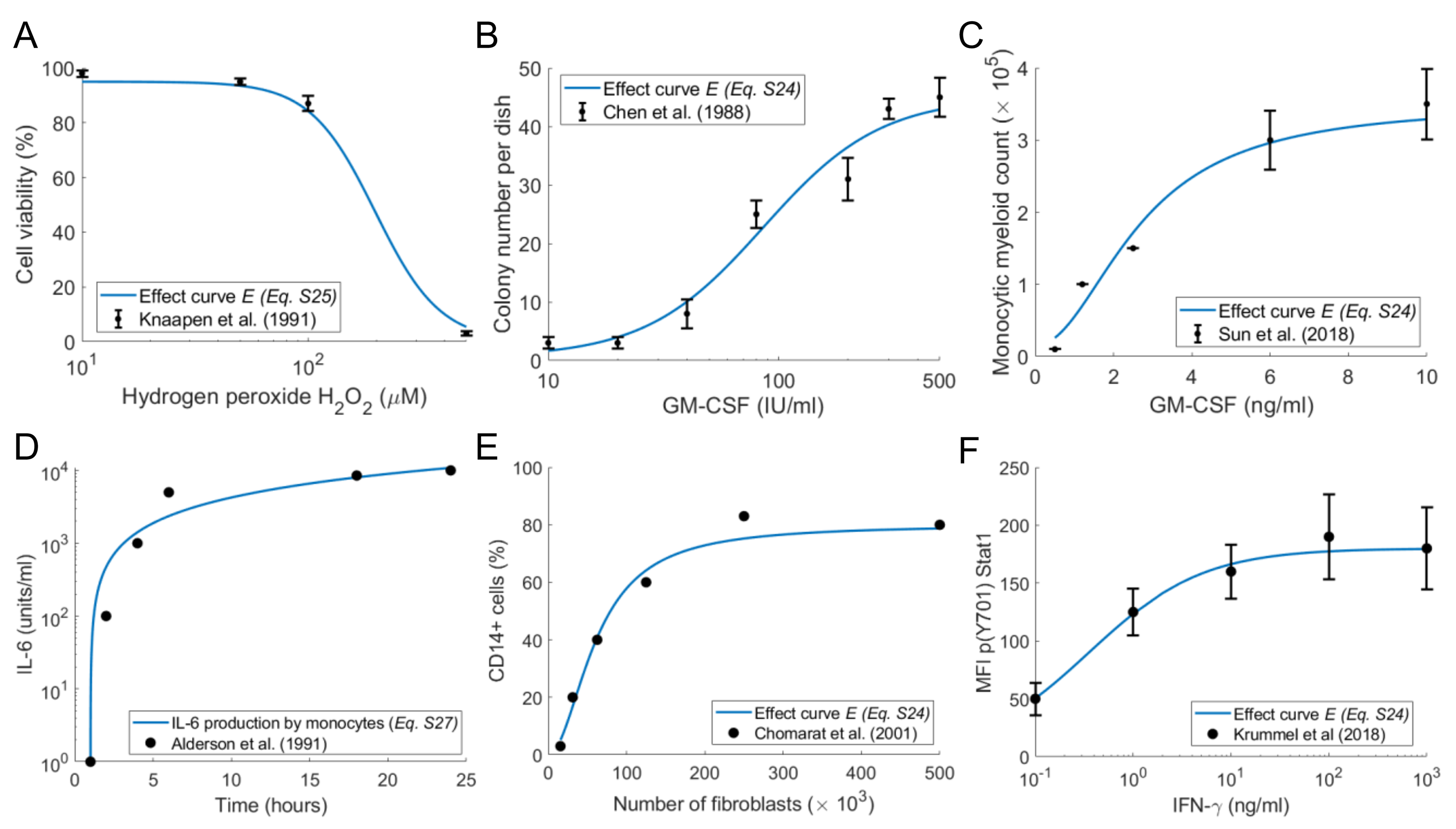

Supplement: S1 Fig — A) Using the measurements by Knaapen et al. [123], the inhibitory effect curve E (Eq. S25) was fit to the cell viability of RLE cells under various concentrations of H2O2. B) The stimulatory effect curve E (Eq. S24) was fit to the dose response measurements of blood monoculture cells (3 × 10^3^ cells/dish) with various concentrations of murine recombinant GM-CSF (IU/ml) [124]. C) The stimulatory effect curve E (Eq. S24) was fit to measurements for the monocytic myeloid cell count as a function of GM-CSF [133] D) Eq. S27 fit to time course data of IL-6 production from monocytes [125]. E) IL-6 stimulation of monocyte differentiation to macrophages modelled by the inhibitory effect curve E (Eq. S24) fit to the percentage of CD14+ cells (macrophages) as a function of the number of fibroblasts measured by Chomarat et al. [44]. F) Stimulatory effect curve E (Eq. S24) for IFN-γ stimulation on CD8+ T cells fit to measurements of the signalling in CD8+T cells for varying doses of IFN-γ [61]. Data (black) is plotted as either circles (D & E) or mean and standard deviation error bars (A-C&F); solid blue line: corresponding fit. (TIF) [file ppat.1009753.s002.tif]

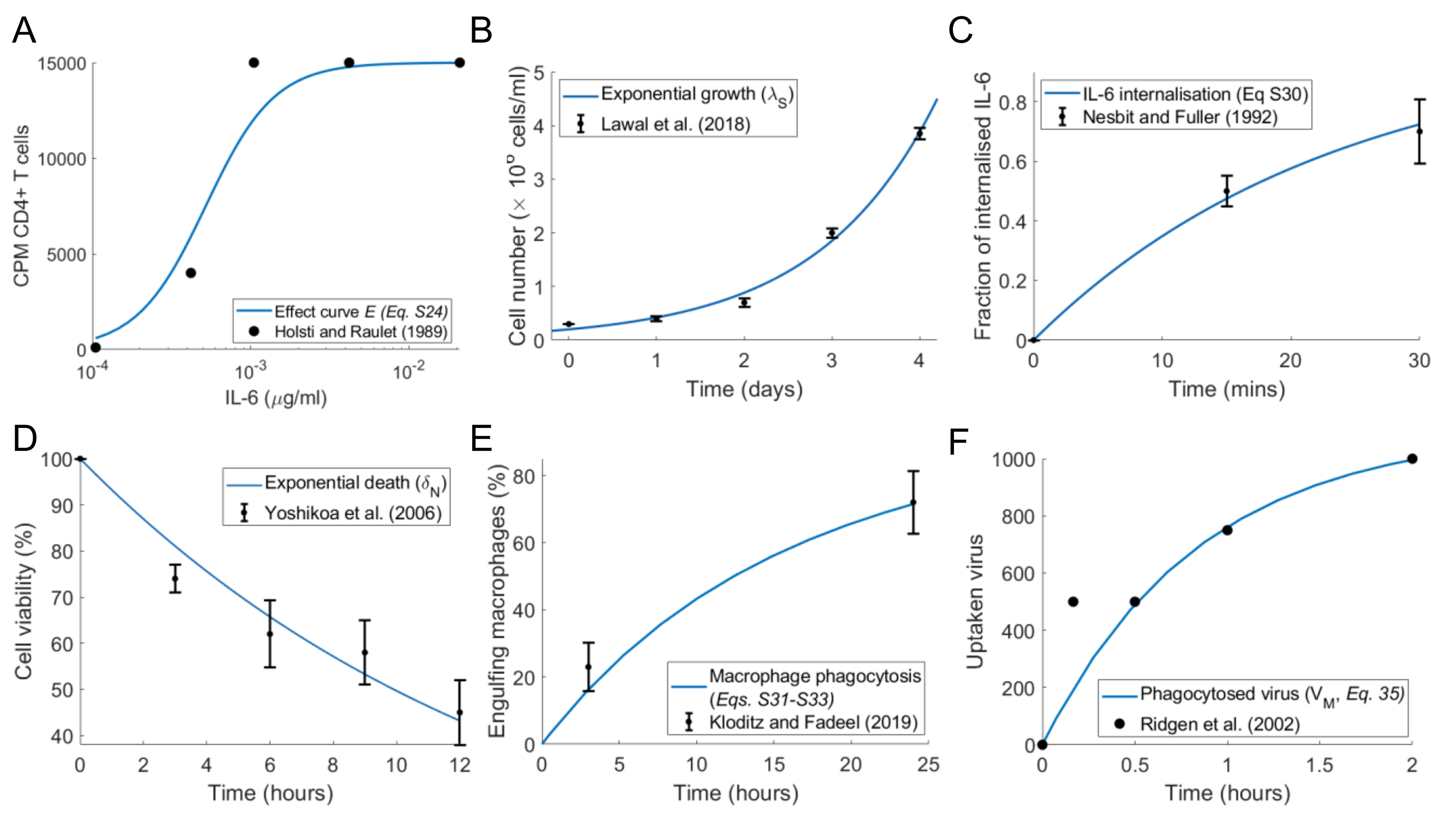

Supplement: S2 Fig — A) Effect curve (Eq. S24) for the IL-6 effect on T cell expansion fit to measurements CD4+ T cells from dilutions of IL-6 by Holsti and Raulet [126]. B) Exponential growth curve fit to the growth of A549 cells [127] C) The internalization rate of IL-6 (Eq. S30) fit to the fraction of internalized IL-6 [128]. D) Exponential decay fit to cell viability after H2O2 administration [129]. E) The macrophage clearance of apoptotic material (Eqs. S31-S33) was fit to the percentage of macrophages that had engulfed material over 25 hours [86]. F) The phagocytosis rate of extracellular virus by macrophages was obtained by fitting Eqs. S34-S35 to the uptake of virus by macrophages measured by Rigden et al. [130]. Data (black) is plotted as either circles (A & F) or mean and standard deviation error bars (B-E); solid blue line: corresponding fit. (TIF) [file ppat.1009753.s003.tif]

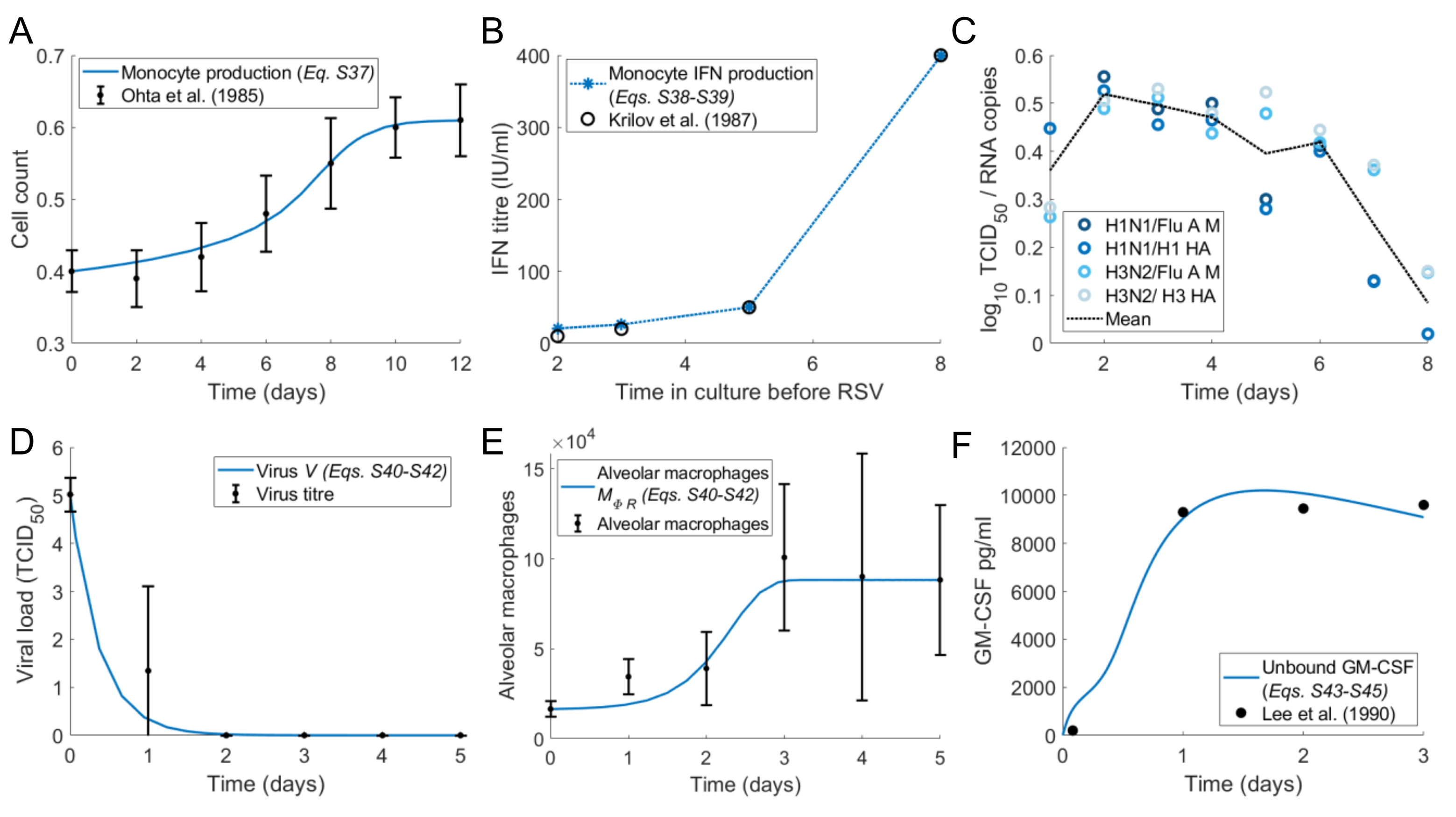

Supplement: S3 Fig — A) Eq. S37 fit to time course of proliferation of monocytes in culture [62]. B) Fit of Eqs. S38-S39 to the production of IFN-α by monocytes after 24 hours with RSV as a function of the number of days of pre-culturing (1, 2, 4 or 7) [63]. C) Correlation between infectious virus titre and RT-PCR copy number for influenza A and B measured by Laurie et al. [134] The relative TCID50 compared to the RNA copies is plotted for each virus strain and the mean as a black dashed line. D-E) Fit of Eqs. S40-S42 to viral loads [135] and alveolar macrophages from experimental influenza infections. F) The production of GM-CSF from stimulated monocytes was recorded by Lee et al. [131] Using a simplified version of the full model (Eqs. S43-S46), we obtained the production rates for monocytes and GM-CSF. Data (black) is plotted as either circles/stars (B&F) or mean and standard deviation error bars (A, D-E); solid blue line: corresponding fit. (TIF) [file ppat.1009753.s004.tif]

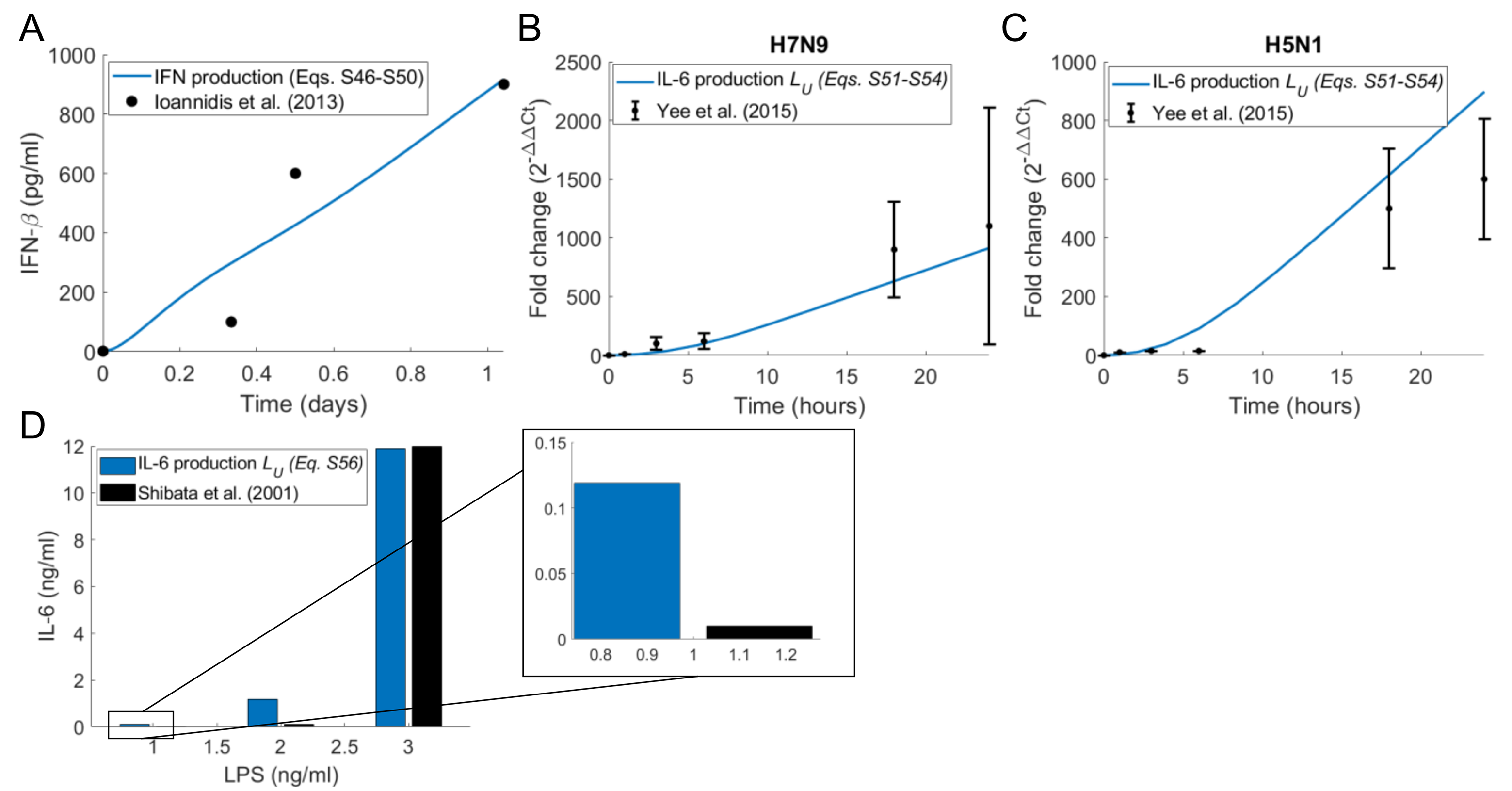

Supplement: S4 Fig — A) Concentration of IFN-β released by alveolar epithelial cells in response to stimulation with influenza virus recorded at 8, 16 and 24 hours [57]. B-C) IL-6 production by infected cells in response to A) H5NA and B) H7N9, measured by Ye et al. [132] Data (black) is plotted as mean and standard deviation error bars with the corresponding fit (Eqs. S51-S54) in solid blue. D) IL-6 production by macrophages (Eq. S56) in response to stimulation with LPS of varying dosage sizes. Shibata et al. [56] measured the production of IL-6 for different dosages of LPS and fitting the production rate to this data to obtain pL,MΦ, ηL,MΦ. (TIF) [file ppat.1009753.s005.tif]

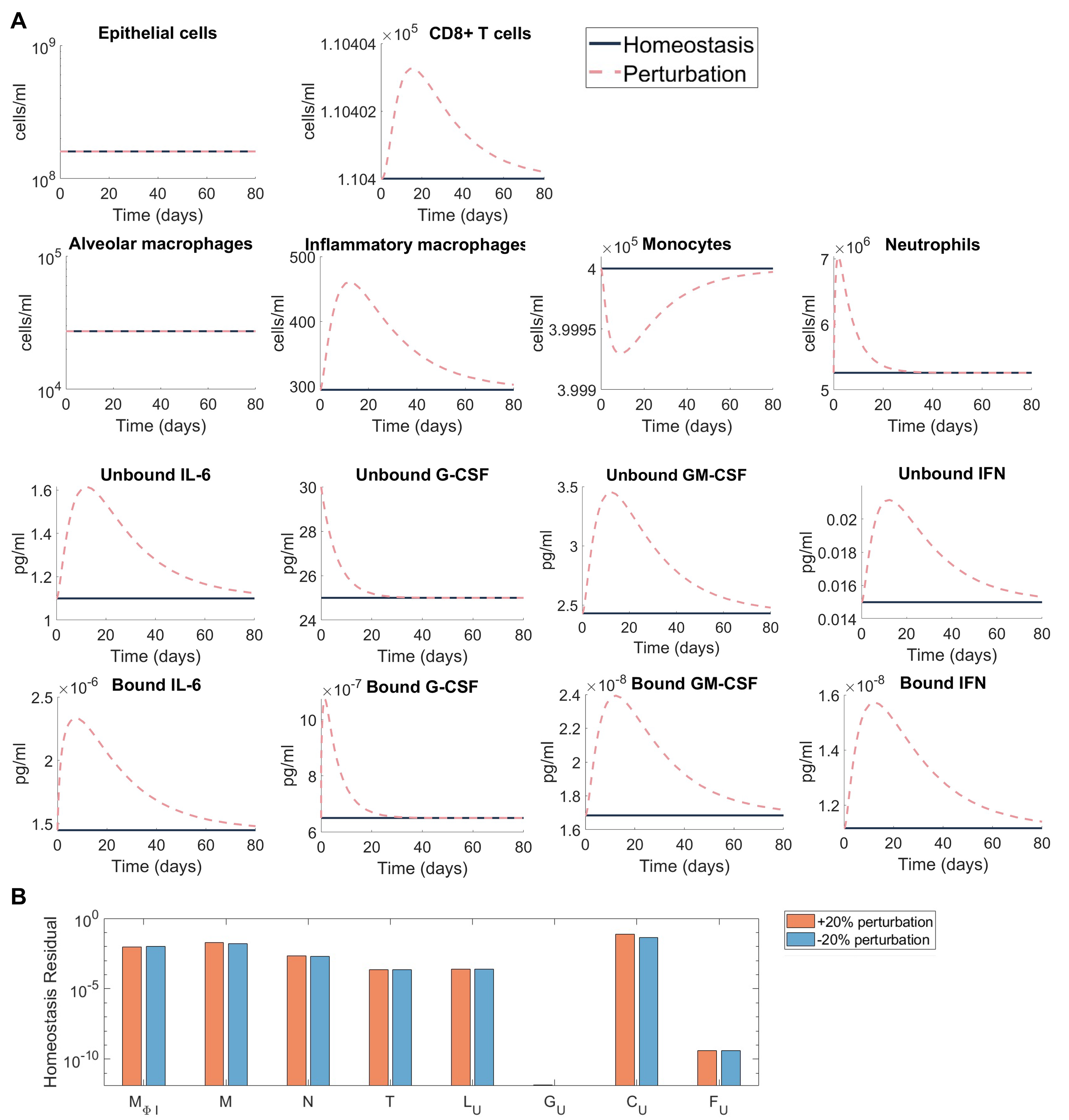

Supplement: S5 Fig — A) To confirm that parameters in the model represented realistic immunocompetent individuals in the disease-free scenario, Eqs. S1-S22 were simulated where V0 = 0 and parameters were given by the homeostasis Eqs. S57-S70. The initial concentration of G-CSF was perturbed and compared to simulations of the model at homeostasis. Simulations at homeostasis are represented by solid lines (purple) and perturbed simulations as dashed lines (pink). B) The maximum residual between variables and their initial conditions at day 50 was measured to confirm that the system was stable for perturbations in all immune cells and cytokines. (TIF) [file ppat.1009753.s006.tif]

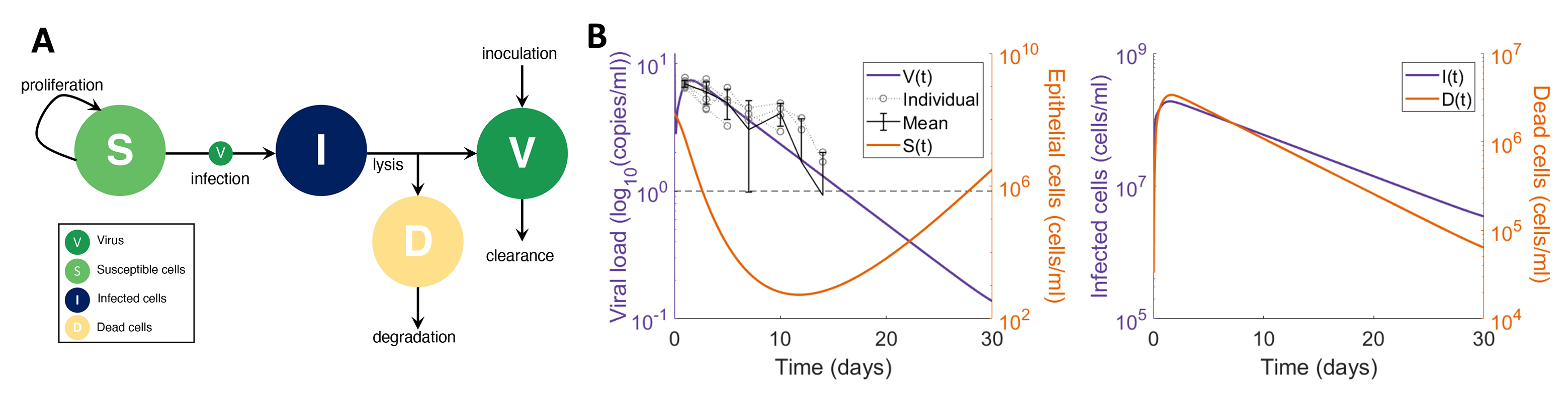

Supplement: S6 Fig — [47] A reduced version of the full model (all cytokine and immune cells set to 0, Eqs 6–9) was fit to data from macaques [47] to estimate preliminary viral kinetic parameters. A) Virus (V) infects susceptible cells (S) making infected epithelial cells (I) which then die to produce dead cells (D) and new virus. B) Comparison of predicted viral dynamics compared to observations from 6 animals, with susceptible cell kinetics (left) with predictions of infected and dead cells over time (right). We estimated β, p, dI, V0 and dV from the reduced model in A) fit to data from Munster et al. [47] measuring the viral load in macaques after challenge with SARS-CoV-2. (TIF) [file ppat.1009753.s007.tif]

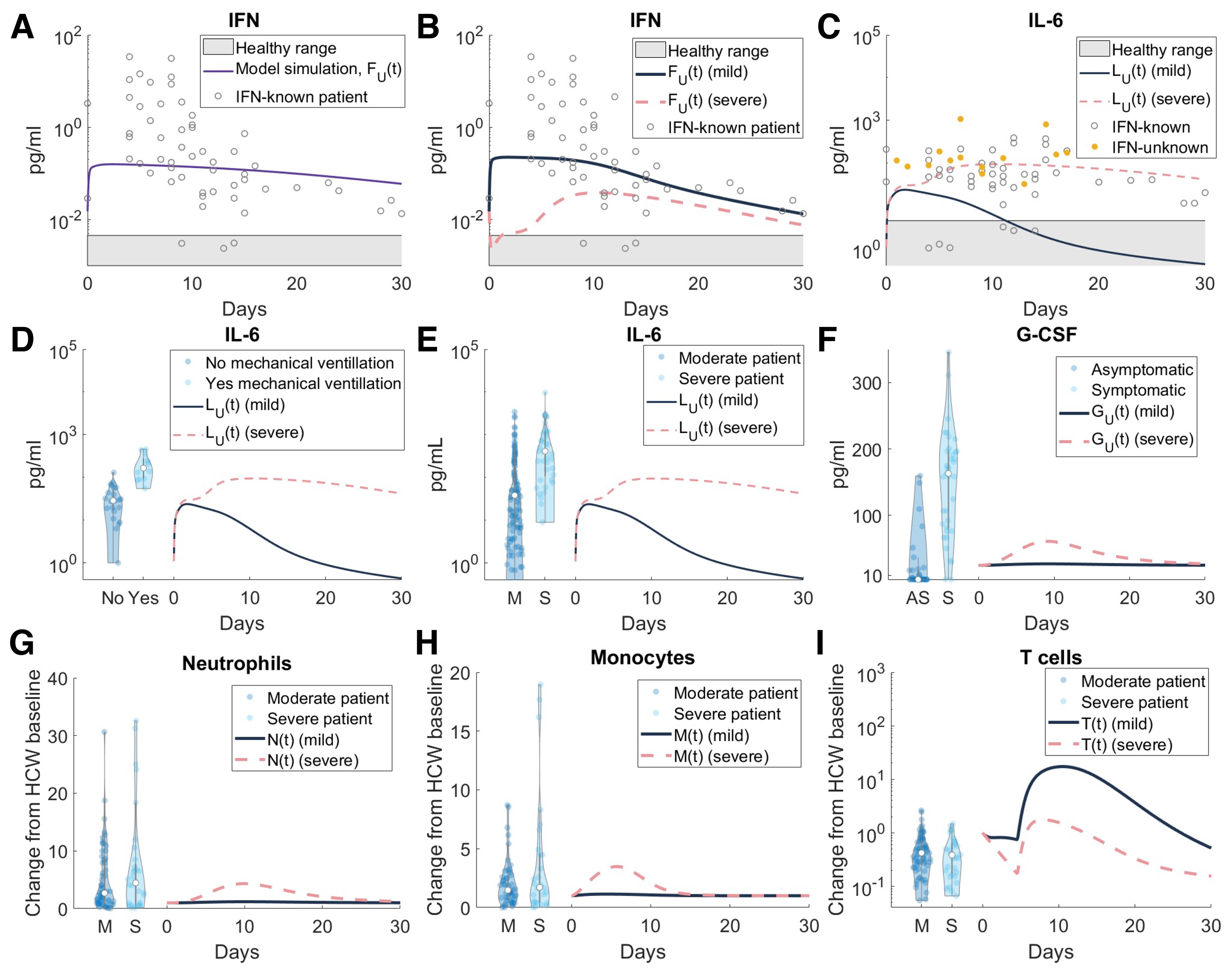

Supplement: S7 Fig — A) IFN dynamics of the reduced model (Fig 3 Main Text) overlaid with patient IFN-α2 plasma concentrations from Trouillet-Assant et al. [100] The solid line (purple) represents the unbound IFN dynamics from the reduced model (Eqs. 27–33). Individual patient IFN-α2 measurements are plotted as grey circles for IFN-positive patients (n = 21), patients with no IFN measurements (IFN-negative; n = 5) have not been plotted. Healthy volunteer IFN-α2 concentrations are indicated by a grey area. B-F) Mild (solid line) and severe (dashed line) dynamics (Eqs. 27–33 corresponding to simulations in Fig 4 Main Text and S8 Fig compared to corresponding measurements in humans. B-C) Plasma IFN-α and IL-6 in COVID-19 critically ill patients (n = 26) obtained by Trouillet-Assant et al. [100] overlaid with mild and severe unbound IFN (FU(t)) and mild and severe unbound IL-6 (LU(t)) IFN-negative patients (yellow stars) had no IFN-α measurements and IFN-positive patients (grey points) had non-zero IFN-α measurements. Healthy volunteer concentrations are indicated by a grey area. D) IL-6 levels in patients requiring (“Yes”) and not requiring mechanical (“No”) ventilation obtained by Herold et al. [53] overlaid with mild and severe unbound IL-6 dynamics. E) IL-6 concentration in Moderate (“M”) and severe (“S”) COVID-19 patients obtained by Lucas et al. [6]. F) G-CSF plasma concentration obtained by Long et al. [30] in symptomatic “S” and asymptomatic “AS” COVID-19 patients overlaid with corresponding mild and severe model dynamics. G-I) Neutrophils, monocytes and CD8+ T cells in moderate and severe COVID-19 patients normalized by health care worker (HCW) baseline measurements obtained by Lucas et al. [6]. Violin plots are given for the measurements plotted in D-I. (TIF) [file ppat.1009753.s008.tif]

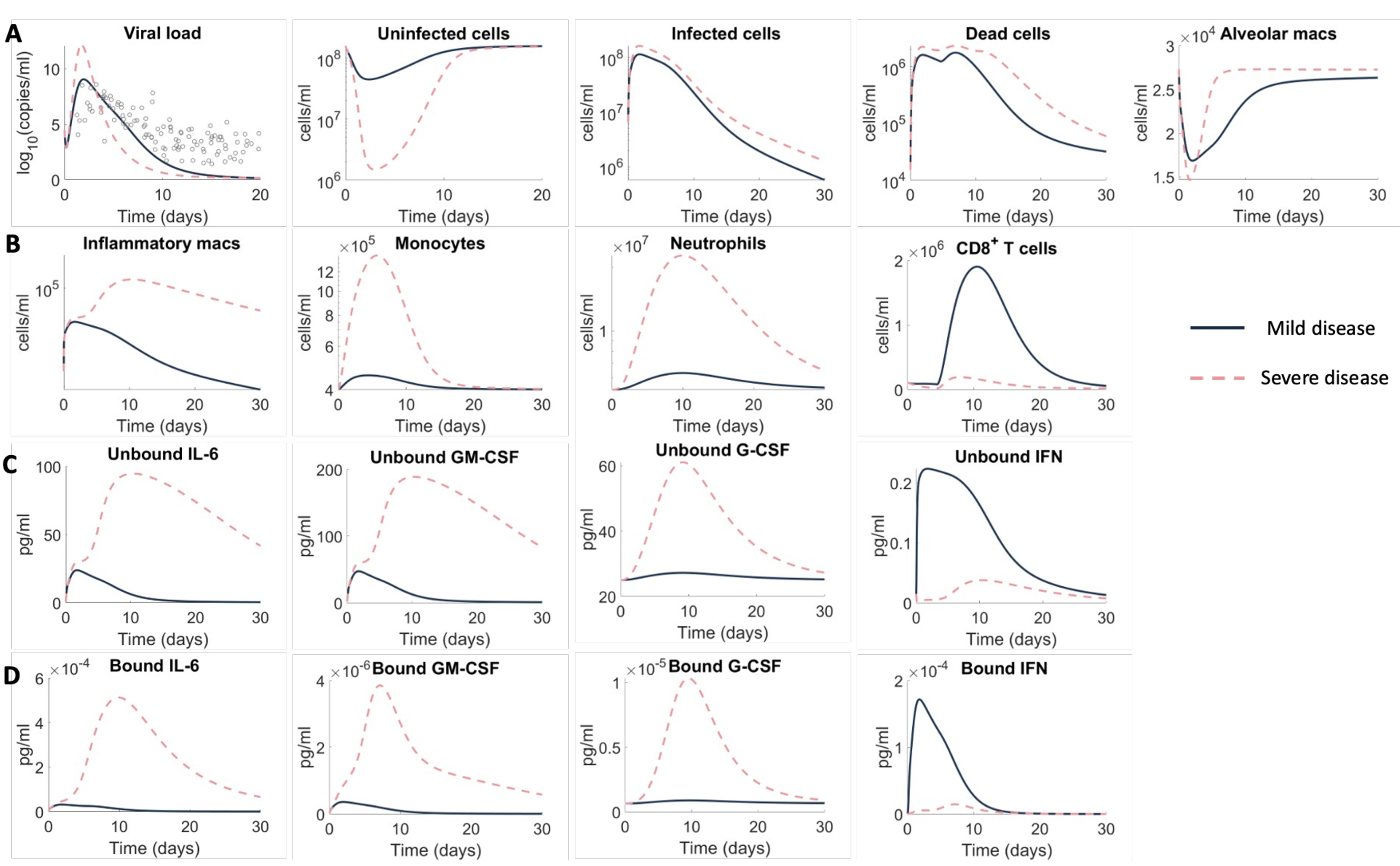

Supplement: S8 Fig — Extension of results of mild and severe disease dynamics in Fig 4 Main Text. Mild disease (solid lines) dynamics obtained by using baseline parameter estimates (S1 Table) while severe disease dynamics (dashed lines) were obtained by decreasing the production rate of type I IFN, pF,I, and increasing the production of monocytes, pM,I, and their differentiation to macrophages, ηF,MΦ. A) Lung cells concentrations (susceptible cells S(t), resistant cells R(t), infected cells I(t), dead cells D(t) and virus V(t)). Solid black line with error bars indicates macaque data (see Fig 2 Main Text). B) Immune cell concentrations (resident macrophages MΦR(t), inflammatory macrophages MΦI(t), monocytes M(t), neutrophils N(t) and T cells T(t)). C) Bound and unbound cytokine concentrations (IL-6 unbound LU(t) and bound LB(t), GM-CSF unbound GU(t) and bound GB(t), G-CSF unbound CU(t) and bound CB(t), type I IFN unbound FU(t) and bound FB(t)). (TIF) [file ppat.1009753.s009.tif]

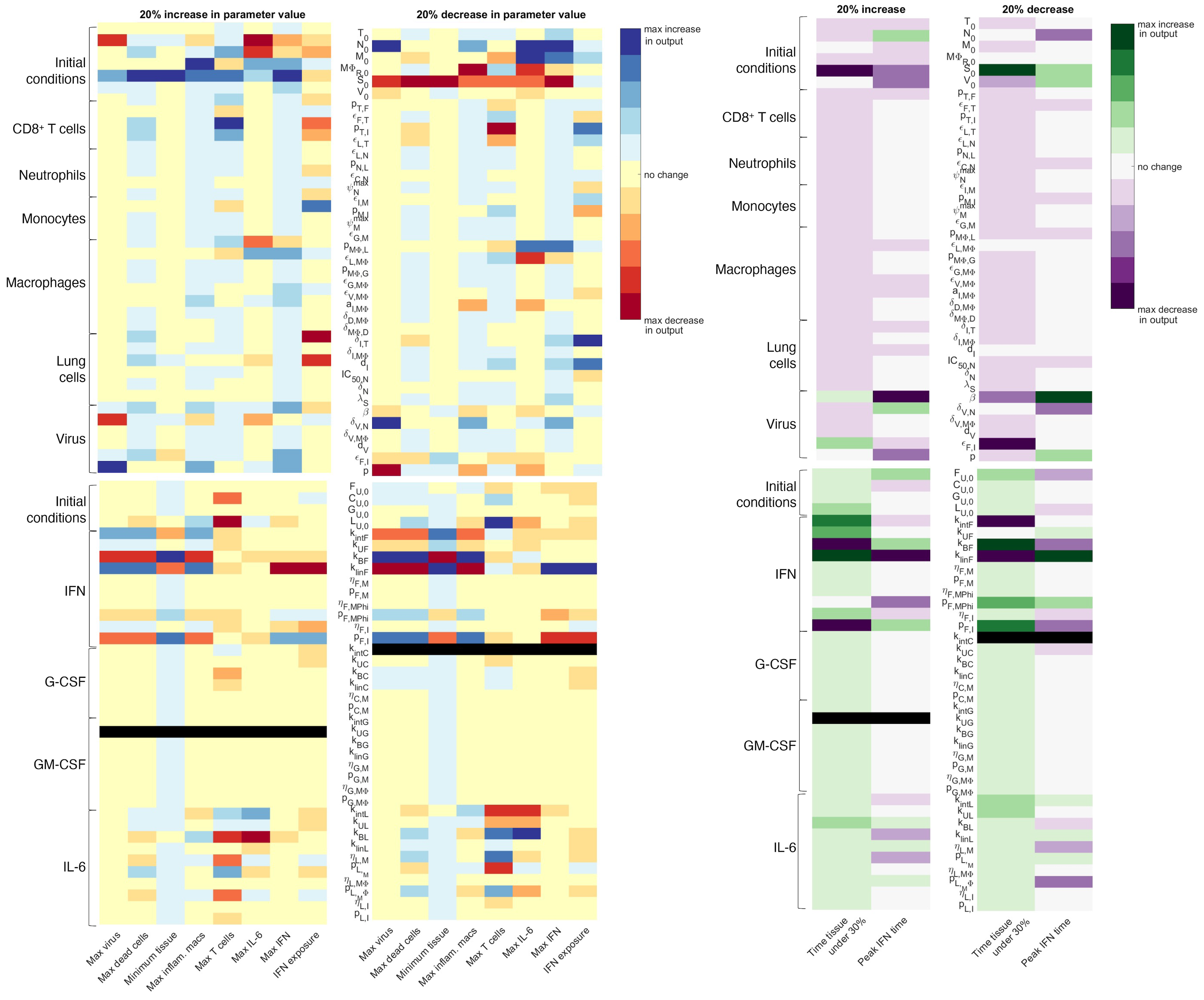

Supplement: S9 Fig — A local sensitivity analysis was performed by varying each parameter ±20% from its originally estimated value and simulating the model. Predictions were then compared to baseline considering: Maximum viral load (max(V)), maximum concentration of dead cells (max(D)), minimum uninfected live cells (min(S+R)), maximum concentration of inflammatory macrophages (max(MΦI)), maximum number of CD8+ T cells (max(T)), maximum concentration of IL-6 (max(LU)), maximum concentration of type I IFN (max(FU)), the total exposure to type I IFN (FU exposure), the number of days damaged tissue was >80% (time (S + R)/Smax)<0.2), and the day type I IFN reached its maximum (day max(FU)). The heatmaps show the fold change of each metric, where blue signifies the minimum value observed and red signifies the maximum value observed, or by the number of days, where light to dark pink signifying increasing number of days from zero. The most sensitive parameters are shown in Fig 5 in the Main Text. (TIF) [file ppat.1009753.s010.tif]

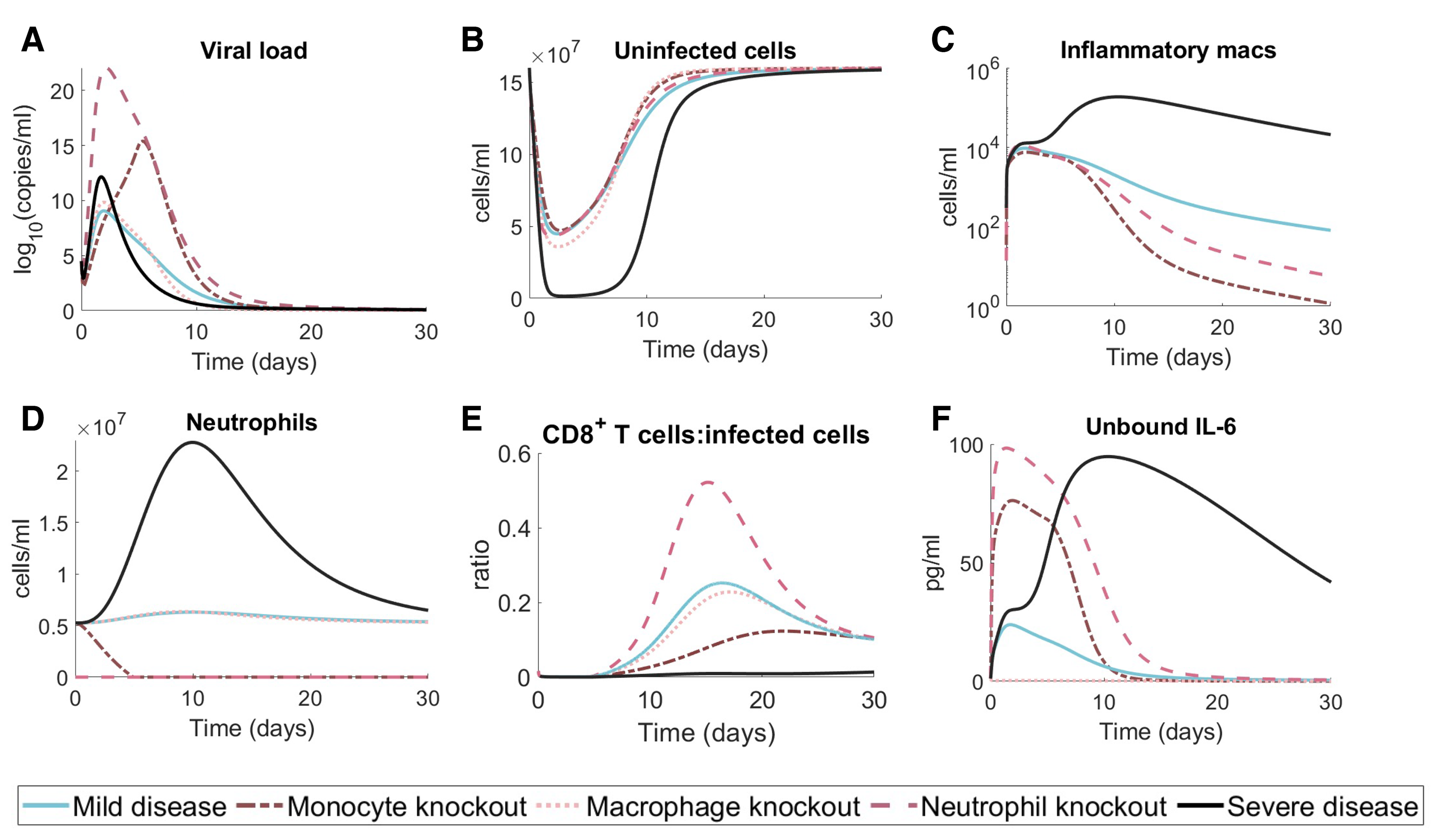

Supplement: S10 Fig — We performed in silico knockout experiments in the mild disease scenario (Fig 4; solid black line) by considering complete monocyte knockout (i.e. no monocyte recruitment and M(0) = 0; dark pink dash-dot line), complete macrophage knockout (i.e. not inflammatory macrophage creation via antigen stimulation or monocyte differentiation; light pink dotted line) and complete neutrophil knockout (i.e. no neutrophil recruitment and N(0) = 0; pink dashed line). Blue solid lines correspond to mild diseases courses; black solid lines: severe disease. Dynamics of the in silico knockout are plotted for the A) viral load, B) uninfected cells, C) inflammatory macrophages, D) neutrophils, E) CD8+ T cells relative to uninfected cells and F) unbound IL-6. This figure is an extension of Fig 7 in the Main Text. (TIF) [file ppat.1009753.s011.tif]

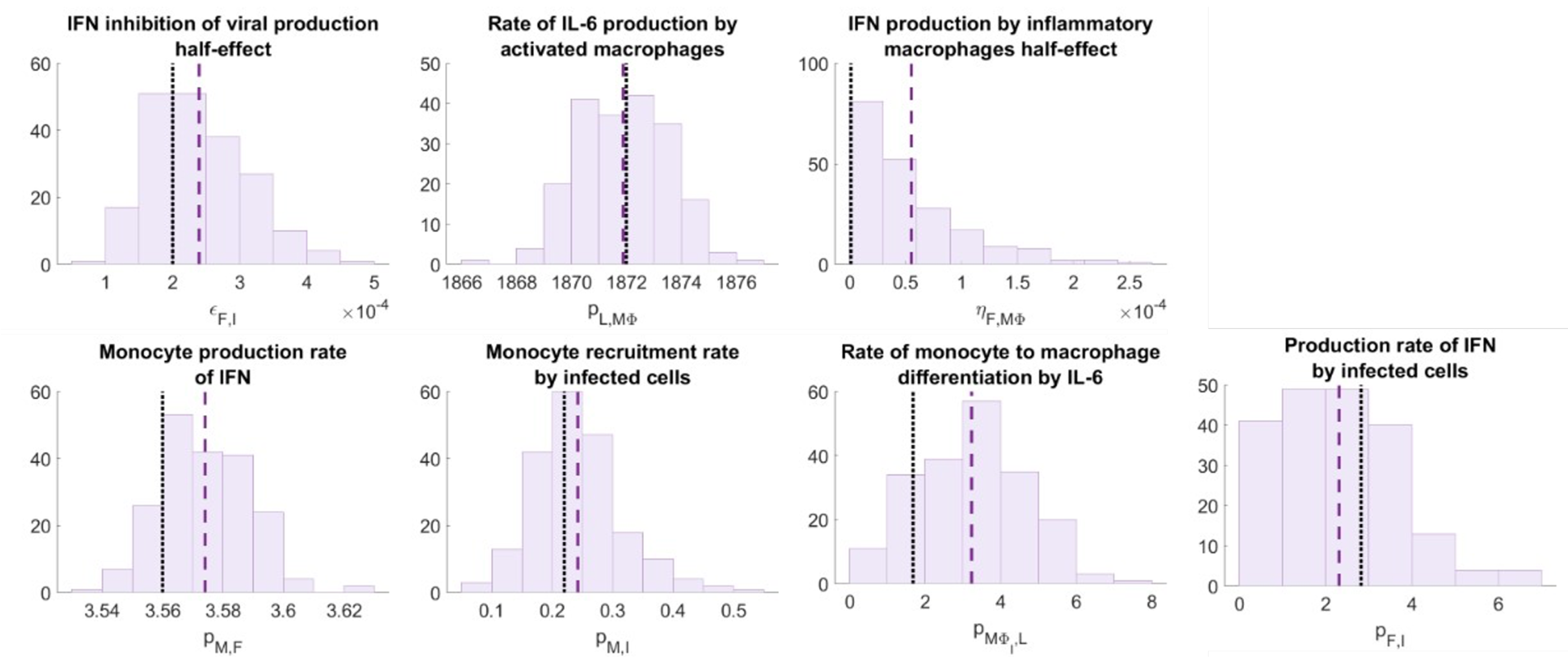

Supplement: S11 Fig — Virtual patients were generated by sampling from normal distributions for a subset of model parameters and optimizing parameters using simulated annealing to confirm realistic disease trajectories (see Fig 4). Resulting distributions for each parameter (purple histograms) are shown compared to estimated values for an average patient (dotted black vertical line). The average of the virtual cohort is also plotted (dashed purple line). (TIF) [file ppat.1009753.s012.tif]

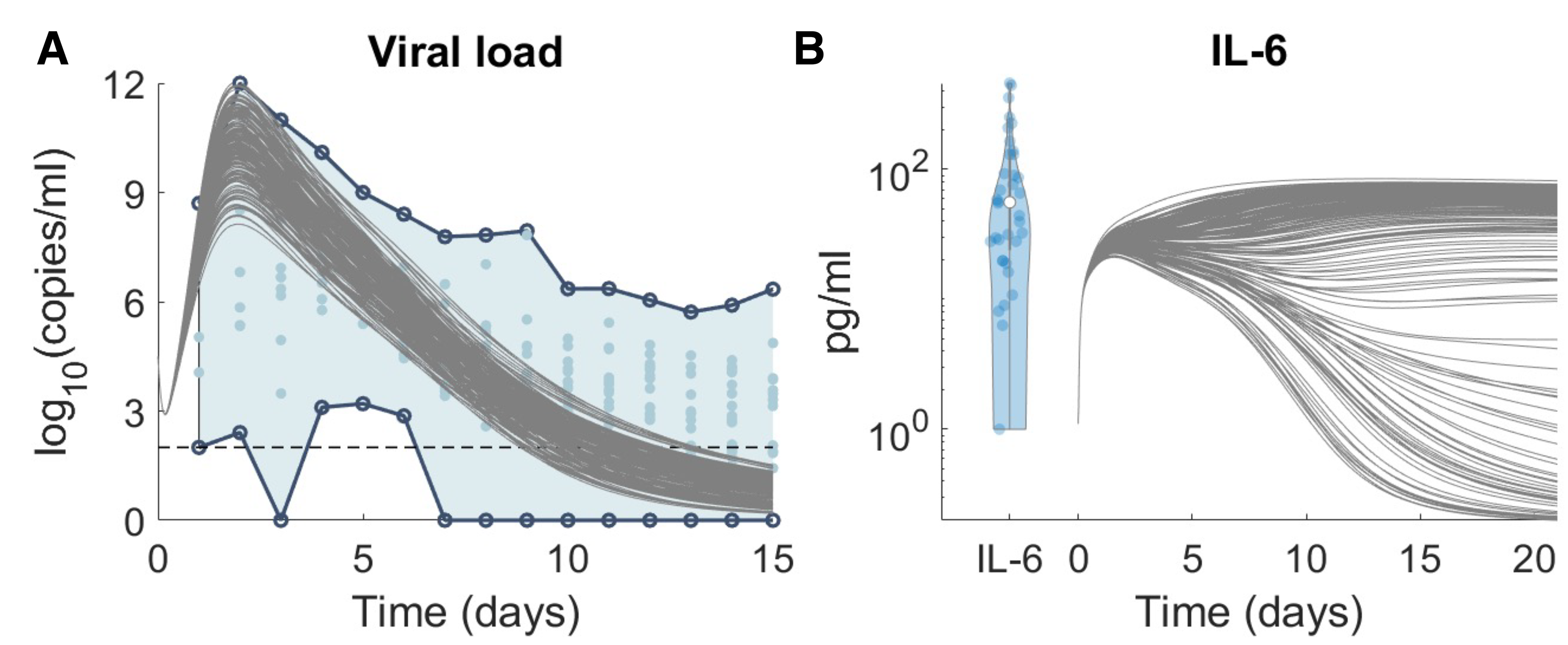

Supplement: S12 Fig — Virtual patients were generated so that viral load, IFN, G-CSF and IL-6 concentration were within physiological ranges obtained in the literature. The physiological ranges (Fig 9) of A) human SARS-CoV-2 viral loads [37], B) IL-6 concentrations from patients [53] plotted against the virtual cohort dynamics (grey lines). (TIF) [file ppat.1009753.s013.tif]
