# Supplementary material for: COVID-19 virtual patient cohort suggests immune mechanisms driving disease outcomes
Source: PLoS Pathog. 2021 Jul 14;17(7):e1009753. doi: 10.1371/journal.ppat.1009753 (PMC8312984; doi:10.1371/journal.ppat.1009753)
Supplement: S1 Table — Parameters have been grouped into: (a-e) cell related, (f-k) cytokine related parameters, and (l) initial conditions. Relevant references are given for parameters directly estimated from the literature (“Direct estimate”). Parameters obtained through fitting to data in the literature have the appropriate figure noted and data referenced (“Fit Fig, data”). Remaining parameters were estimated from homeostasis calculation (“Homeostasis”) or qualitatively estimated (“Estimated”). Parameters whose value was taken from another parameters estimation has that parameter noted. Viral load is reported as log(virion copies/ml) and cells have been noted in 109 cells/ml. Time t is in days. The final sub-table (m) is a list of the variables in the model. (PDF) [file ppat.1009753.s014.pdf]

a) Viral kinetic parameters

Param	Units	Description	Value	Estimation and citation
p	1/day ×log(cop/ml) /10^9cells	Lytic viral production rate	2.59	Direct estimate [1]
λ_S	1/day	Proliferation of epithelial cells	0.74	Fit S2 Fig, data [2]
S_{max}	10^9cells	Epithelial cells carrying capacity	S_0	Direct estimate [3]
$\lambda_{M\Phi}$	log(cop/ml)/day	Production of alveolar macrophages	5943	Fit S3 Fig, data [4]
$M_{\Phi max}$	10^9cells/ml	Alveolar macrophage carrying capacity	$M_{\Phi R,0}$	Fit S3 Fig
β	1/day× 1/ log(cop/ml)	SARS-CoV-2 virus infection rate	0.29	Fit Fig 2, data [1]
τ_I	day	Eclipse time	0.17	Direct estimate [5]
d_I	1/day	Death rate of infected cells	0.1	Fit Fig 2, data [1]
τ_T	1/day	Delay in $CD8^+$ T cell arrival	4.5	Direct estimate [6]

b) Cell production, recruitment, and activation rates

Param	Units	Description	Value	Estimation and citation
$p_{M_{\Phi I},G}$	1/day	Monocyte to macrophage differentiation by GM-CSF	1.7	Direct estimate [7]
$p_{M_{\Phi I},L}$	1/day	Monocyte to macrophage differentiation by IL-6	1.7	Direct estimate [7]
$a_{I,M\Phi}$	ml/(10^9cells)× (1/day)	Activation of macs by infected and dead cells	1.1×10^3	Direct estimate [8,9]
$p_{M,I}$	1/day	Monocyte recruitment rate by infected cells	0.22	Direct estimate [10]
$p_{T,F}$	1/day	$CD8^+$ T cell production rate by IFN	4	Direct estimate [11]
$p_{N,L}$	1/day	Neutrophils recruitment rate by IL-6	0.21	Homeostasis
$p_{T,L}$	1/day	$CD8^+$ T cells recruitment rate by IL-6	4	Direct estimate [11]
$p_{T,I}$	1/day	$CD8^+$ T cell proliferation rate	1	Direct estimate [12]
M^*_{prod}	1/day	Homeostasis reservoir release rate	0.13	Homeostasis
ψ_M^{max}	1/day	Maximal reservoir release rate	11.55	Direct estimate [13]
N^*_{prod}	1/day	Homeostasis reservoir release rate	0.21	Homeostasis
ψ_N^{max}	1/day	Maximal reservoir release rate	4.13	Direct estimate [14]
C^*_{BF}	Dimensionless	Homeostasis neutrophil receptor bound fraction	1.6×10^{-5}	Direct estimate [14]

c) Cell-related half-effect (ϵ), IC50 (IC_{50}), and Hill coefficient (h) parameters

Param	Units	Description	Value	Estimation and citation
$\epsilon_{F,I}$	pg/ml	IFN inhibition of viral production	4.7×10^{-4}	Direct estimate [15]
ϵ_{L,M_Φ}	pg/ml	IL-6 monocytes to macrophages	0.011	Fit S1 Fig, data [16]
$\epsilon_{G,M_{\Phi I}}$	pg/ml	GM-CSF monocyte to macrophages	0.027	Fit S1 Fig, data [17]
$\epsilon_{G,M}$	pg/ml	GM-CSF recruitment of monocytes	57.2	Fit S1 Fig, data [18]
$\epsilon_{F,T}$	pg/ml	IFN production of $CD8^+$ T cells	0.004	Fit S1 Fig, data [19]

$\epsilon_{C,N}$	unitless	G-CSF recruitment of neutrophils	1.89×10^{-4}	Fit S1 Fig, data [14]
$\epsilon_{L,N}$	pg/ml	IL-6 recruitment of neutrophils	57.2	Estimated: $\epsilon_{G,M}$
$\epsilon_{I,M}$	10^9cells/ml	Infected cell monocyte recruitment	0.11	Estimated
$\epsilon_{L,T}$	pg/ml	IL-6 production of $CD8^+$ T cells	3×10^{-4}	Fit S1 Fig, data [20]
$\epsilon_{V,M\Phi}$	log(cop/ml)	Viral load for mac replenishing	2.96	Fit S3 Fig
$\epsilon_{T,I}$	10^9 cells/ml	Antigen driven proliferation	10^{-6}	Direct estimate [12]
h_M	Dimensionless	GM-CSF monocyte recruitment	1.67	Fit S1 Fig, data [18]
$h_{M,M\Phi}$	Dimensionless	GM-CSF monocyte to macrophages	2.03	Fit S1 Fig, data [17]
h_N	Dimensionless	Neutrophil induced damage	3.02	Fit S1 Fig, data [21]
$IC_{50,N}$	10^9 cells/ml	Neutrophil induced damage	0.047	Fit S1 Fig, data [21]

d) Cell/virus-induced death rates

Param	Units	Description	Value	Estimation and citation
$\delta_{V,M\Phi}$	ml/(10^9cells)× 1/day	Rate of viral clearance by macrophages	768	Fit S2 Fig, data [22]
$\delta_{V,N}$	ml/(10^9cells)× 1/day	Rate of viral clearance by neutrophils	2304	Estimated
δ_N	1/day	Rate of neutrophil inflicted damage	1.68	Fit S2 Fig, data [23]
ρ	Dimensionless	Bystander death modulation constant	0.5	Estimated
$\delta_{I,M\Phi}$	ml/(10^9cells)× (1/day)	Rate macrophages phagocytose infected cells	121	Direct estimate [24]
$\delta_{I,T}$	ml/(10^9cells)× (1/day)	Rate $CD8^+$ T cells induce apoptosis in infected cells	238	Direct estimate [25]
$\delta_{M\Phi,D}$	ml/(10^9cells)× (1/day)	Rate macrophages die from phagocytosis	6.06	Direct estimate [26,27]
$\delta_{D,M\Phi}$	ml/(10^9cells)× (1/day)	Rate macrophages phagocytose dead cells	8.03	Fit S2 Fig, data [26]

e) Cell death and virus decay rates

Param	Units	Description	Value	Estimation and citation
d_V	1/day	Viral decay rate	1.81	Fit Fig 2, data [28]
d_D	1/day	Degradation rate of apoptosed cells	8	Direct estimate [29]
$d_{M_{\Phi R}}$	1/day	Alveolar macrophage death rate	0	Direct estimate [30]
$d_{M_{\Phi I}}$	1/day	Inflammatory macrophage death rate	0.3	Direct estimate [31]
d_M	1/day	Monocyte death rate	0.76	Direct estimate [32]
d_N	1/day	Neutrophil death rate	1.28	Direct estimate [33]
d_T	1/day	$CD8^+$ T cell death rate	0.4	Direct estimate [34]

f) Cytokine production rates

Param	Units	Description	Value	Estimation and citation
$p_{L,I}$	pg/ml/day	IL-6 production by infected cells	11.89	Fit S4 Fig, data [35]
$p_{L,M_{\Phi I}}$	pg/ml/day	IL-6 production by inflammatory macrophages	1872	Fit S5 Fig, data [36]
$p_{L,M}$	pg/ml/day	IL-6 production by monocytes	72.56	Fit S1 Fig, data [37]

$p_{G,M_{\Phi I}}$	pg/ml/day	GM-CSF production by inflammatory macrophages	2626	Direct estimate [38]
$p_{C,M}$	ng/ml/day	G-CSF production by monocytes	26.26	Estimated: $p_{G,M_{\Phi I}}$
$p_{G,M}$	pg/ml/day	GM-CSF production by monocytes cells	3070	Fit S3 Fig, data [39]
$p_{F,I}$	pg/ml/day	IFN production by infected cells	2.82	Fit S3 Fig, data [40]
$p_{F,M_{\Phi I}}$	pg/ml/day	IFN production by inflammatory macrophages	1.3	Estimated
$p_{F,M}$	pg/ml/day	IFN production by monocytes	3.56	Fit S3 Fig, data [41,42]

g) Cytokine production half-effect (η) and Hill coefficient (h) parameters

**Param**	**Units**	**Description**	**Value**	**Estimation and citation**
$\eta_{L,I}$	10^9 cells/ml	IL-6 production by infected cells	0.7	Fit S4 Fig, data [35]
$\eta_{L,M}$	10^9 cells/ml	IL-6 by monocytes	0.0045	Fit S1 Fig, data [37]
$\eta_{L,M_{\Phi I}}$	10^9 cells/ml	IL-6 by inflammatory macrophages	3.6×10^{-5}	Fit S4 Fig, data [36]
$\eta_{G,M\Phi}$	10^9 cells/ml	GM-CSF by macrophages	3.6×10^{-5}	Estimated: $\eta_{L,M\Phi}$
$\eta_{G,M}$	10^9 cells/ml	GM-CSF by monocytes	0.15	Homeostasis
$\eta_{C,M}$	10^9 cells/ml	G-CSF by monocytes	3.05	Homeostasis
$\eta_{F,I}$	10^9 cells/ml	IFN by infected cells	0.011	Fit S4 Fig, data [40]
$\eta_{F,M_{\Phi I}}$	10^9 cells/ml	IFN by inflammatory macrophages	1.3×10^{-6}	Homeostasis
$\eta_{F,M}$	10^9cells/ml	IFN by monocytes	0.54	Fit S3 Fig, data [41,42]

h) Cytokine linear (renal) clearance and internalization rates

**Param**	**Units**	**Description**	**Value**	**Estimation and citation**
k_{lin_L}	1/day	Rate of IL-6 renal clearance	16.6	Direct estimate [43]
k_{lin_G}	1/day	Rate of GM-CSF renal clearance	11.7	Direct estimate [44]
k_{lin_C}	1/day	Rate of G-CSF renal clearance	0.16	Direct estimate [14]
k_{lin_F}	1/day	Rate of IFN renal clearance	18	Direct estimate [45]
k_{int_L}	1/day	Internalization rate of IL-6	61.8	Fit S2 Fig, data [46]
k_{int_G}	1/day	Internalization rate of GM-CSF	73.4	Direct estimate [47]
k_{int_C}	1/day	Internalization rate of G-CSF	462	Direct estimate [14]
k_{int_F}	1/day	Internalization rate of IFN	17	Direct estimate [48]

i) Cytokine binding/unbinding rates and stoichiometric constant

**Param**	**Units**	**Description**	**Value**	**Estimation and citation**
k_{B_L}	ml/pg/day	IL-6 binding rate	0.0018	Direct estimate [49]
k_{B_G}	ml/pg/day	GM-CSF binding rate	0.0021	Direct estimate [47]
k_{B_C}	ml/ng/day	G-CSF binding rate	2.24	Direct estimate [14]
k_{B_F}	ml/pg/day	IFN binding rate	0.011	Direct estimate [48]
k_{U_L}	1/day	IL-6 unbinding rate	22.3	Direct estimate [49]
k_{U_G}	1/day	GM-CSF unbinding rate	522	Direct estimate [47]
k_{U_C}	1/day	G-CSF unbinding rate	184	Direct estimate [14]

k_{U_F}	1/day	IFN unbinding rate	6.07	Direct estimate [48]
POW	Dimensionless	Stoichiometric constant (G-CSF)	1.4608	Direct estimate [14]
		Stoichiometric constant (IL-6, GM-CSF, IFN)	1	
$\hat{p}$	Dimensionless	Stoichiometry relating constant (G-CSF)	2	Direct estimate [14]
		Stoichiometry relating constant (IL-6, GM-CSF, IFN)	1	

j) Number of cellular receptors and cytokine molecular weights

**Param**	**Units**	**Description**	**Value**	**Estimation and citation**
$K_{L,N}$	sites/cell	No. IL-6 receptors on neutrophils	720	Direct estimate [50]
$K_{L,T}$	sites/cell	No. IL-6 receptors on T cells	300	Direct estimate [51]
$K_{L,M}$	sites/cell	No. of IL-6 receptors on monocytes	509	Direct estimate [52]
$K_{G,M}$	sites/cell	No. GM-CSF receptors on monocyte	1058	Direct estimate [53]
$K_{C,N}$	sites/cell	No. of G-CSF receptors on neutrophil	600	Direct estimate [54]
$K_{F,T}$	sites/cell	No. of IFN receptors on T cells	1000	Direct estimate [55]
$K_{F,I}$	sites/cell	No. of IFN receptors on infected cells	1300	Direct estimate [56]
MM_L	g/mol	Molecular weight of IL-6	21000	Direct estimate [57]
MM_G	g/mol	Molecular weight of GM-CSF	14000	Direct estimate [58]
MM_C	g/mol	Molecular weight of G-CSF	19600	Direct estimate [14]
MM_F	g/mol	Molecular weight of IFN-β	19000	Direct estimate [59]

k) Initial conditions

**Param**	**Units**	**Description**	**Value**	**Estimation and citation**
V_0	log(copies/ml)	Initial viral load	4.5	Direct estimate [1]
S_0	10^9 cells/ml	Initial susceptible cells	0.16	Direct estimate [3,60]
I_0	10^9 cells/ml	Initial infected cells	0	Estimated
R_0	10^9 cells/ml	Initial resistant cells	0	Estimated
$M_{\Phi R,0}$	10^9 cells/ml	Initial resident macrophages	2.7×10^{-5}	Direct estimate [3]
$M_{\Phi I,0}$	10^9 cells/ml	Initial inflammatory macrophages	2.9×10^{-7}	Homeostasis
M_0	10^9 cells/ml	Initial monocytes	0.0004	Direct estimate [61]
M_R	10^9 cells/ml	Initial reservoir monocytes	0.0023	Direct estimate [13]
N_0	10^9 cells/ml	Initial neutrophils	0.0053	Direct estimate [14]
N_R	10^9 cells/ml	Initial reservoir neutrophils	0.0316	Direct estimate [14]
T_0	10^9 cells/ml	Initial $CD8^+$ T cells	1.1×10^{-4}	Direct estimate [62,63]
$L_{U,0}$	pg/ml	Initial unbound IL-6	1.1	Direct estimate [64]
$L_{B,0}$	pg/ml	Initial bound IL-6	1.4×10^{-6}	Homeostasis
$G_{U,0}$	pg/ml	Initial unbound GM-CSF	2.43	Direct estimate [65]
$G_{B,0}$	pg/ml	Initial bound GM-CSF	1.6×10^{-8}	Homeostasis
$C_{U,0}$	ng/ml	Initial unbound G-CSF	0.025	Direct estimate [14]
$C_{B,0}$	ng/ml	Initial bound G-CSF	6.5×10^{-10}	Direct estimate [14]
$F_{U,0}$	pg/ml	Initial unbound IFN	0.015	Direct estimate [66]
$F_{B,0}$	pg/ml	Initial bound IFN	1.1×10^{-8}	Homeostasis

m) List of variables in ***Eqs.*** **S1-S22**

**Variable**	**Units**	**Description**
V	cop/ml	Viral load
S	10^9 cells/ml	Susceptible cells
I	10^9 cells/ml	Infected cells
R	10^9 cells/ml	Resistant cells
$M_{\Phi R}$	10^9 cells/ml	Alveolar (resident) macrophages
$M_{\Phi I}$	10^9 cells/ml	Inflammatory macrophages
M	10^9 cells/ml	Monocytes
M_R	10^9 cells/ml	Bone marrow reservoir monocytes
N	10^9 cells/ml	Neutrophils
N_R	10^9 cells/ml	Bone marrow reservoir neutrophils
T	10^9 cells/ml	$CD8^+$ T cells
L_U	pg/ml	Unbound IL-6
L_B	pg/ml	Bound IL-6
G_U	pg/ml	Unbound GM-CSF
G_B	pg/ml	Bound GM-CSF
C_U	ng/ml	Unbound G-CSF
C_B	ng/ml	Bound G-CSF
C_{BF}	unitless	Neutrophil G-CSF receptor bound fraction
F_U	pg/ml	Unbound IFN
F_B	pg/ml	Bound IFN
